# High-throughput encapsulated nanodroplet screening for accelerated co-crystal discovery†

**DOI:** 10.1039/d4sc07556k

**Published:** 2025-04-22

**Authors:** Jessica P. Metherall, Philip A. Corner, James F. McCabe, Michael R. Probert, Michael J. Hall

**Affiliations:** a Chemistry, School of Natural and Environmental Sciences, Newcastle University Newcastle upon Tyne UK michael.hall@newcastle.ac.uk; b Early Product Development & Manufacturing, Pharmaceutical Sciences, BioPharmaceuticals R&D, AstraZeneca Macclesfield UK

## Abstract

Co-crystals are composed of two or more chemically inequivalent molecular species, excluding solvents, generally in a stoichiometric ratio. Co-crystals are particularly important in pharmaceutical development, where a suitable co-crystal can significantly improve the physiochemical and pharmacokinetic properties of an active pharmaceutical ingredient. However, co-crystal discovery remains both practically challenging and resource intensive, requiring the extensive searching of complex experimental space. Herein, we demonstrate a high-throughput (HTP) nanoscale co-crystallisation method for the rapid screening of large areas of co-crystallisation space with minimal sample requirements, based on Encapsulated Nanodroplet Crystallisation (ENaCt). HTP co-crystallisation screening by ENaCt allowed rapid access to all 18 possible binary co-crystal combinations of 3 small molecules and 6 co-formers (A/B), through the use of 3456 individual experiments exploring solvent, encapsulating oil and stoichiometry, including 10 novel binary co-crystal structures elucidated by single crystal X-ray diffraction (SCXRD). Higher-order co-crystal (HOC) discovery, accessing co-crystals containing three or more molecules, is one of the most challenging co-crystal research areas, due to the highly complex experimental landscape that must be navigated. Herein, we further exemplify the power of ENaCt co-crystallisation by application to HOC discovery. HTP ENaCt co-crystallisation screening of three component (A/B/C) and four component (A/B/C/D) combinations gave ready access to both ternary and quaternary HOCs, each containing three or four different molecular species respectively. In total, 13 056 individual ENaCt experiments are presented resulting in 54 co-crystal structures by SCXRD, including 17 novel binary co-crystals, 8 novel ternary co-crystals and 4 novel quaternary co-crystals. ENaCt co-crystallisation is thus demonstrated to be a highly impactful and efficient tool in the search for small molecule co-crystals, through the employment of parallelised HTP nanoscale experimental workflows.

## Abstract 2

A high-throughput nanoscale co-crystallisation approach is presented, that combines automation and small scale to enable the exploration of co-crystallisation conditions, resulting in 30 new binary, ternary and quaternary co-crystals.

## Graphical abstract

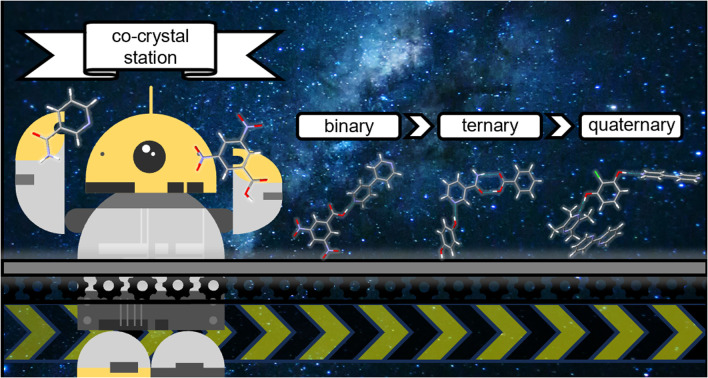

## Introduction

A co-crystal can be defined as a crystalline solid containing two or more chemically inequivalent molecules, generally, present in a stoichiometric ratio, and excluding simple salts or solvates.^1^ Co-crystals have altered physiochemical properties in comparison to a simple mixture of crystals of the individual components, and this effect has been exploited in many research areas, including supramolecular and functional materials,^2^ pigments,^3^ agrochemicals,^4^ energetic materials^5^ and particularly pharmaceuticals.^6^ In the case of pharmaceuticals, the differing physical properties of a co-crystal containing an active pharmaceutical ingredient (API) are commonly used during formulation to improve stability and bioavailability,^7^ and API co-crystal forms can be a key element of associated intellectual property.^8^

Due to the importance of small molecule co-crystals, considerable effort has been devoted to their discovery using both classical and modern crystallisation methods, including neat and liquid-assisted grinding,^9^ melt microdroplets,^10^ laser irradiation of mixed powders,^11^ sonocrystallisation,^12^ and heteronuclear seeding.^13^ These experimental efforts are typically supported with empirical and experimentally driven crystal engineering methods,^14^ particularly the use of supramolecular synthons,^15^ and increasingly paired with computational methods for co-crystal predication and design.^16^

However, successful crystallisation of multi-component systems still remains experimentally challenging, due to the large number of experimental variables that must be explored. The rigorous exploration of such a large experimental landscape for a molecule of interest is thus best attempted through high-throughput (HTP) approaches.^17^ Despite considerable advances in the area,^18^ state-of-the-art HTP co-crystal screening for small molecules still requires access to gram quantities of substrate, with milligrams of material used per experiment. In addition, experimental read-outs are typically reliant on Raman spectroscopy and/or powder X-ray diffraction to identify the presence of new crystal forms, with follow-up crystal growth and single crystal X-ray diffraction (SCXRD) needed to validate hits. Thus, particularly with sample limited material, only a partial exploration of the available experimental space is performed. Recently, we have developed Encapsulated Nanodroplet Crystallisation (ENaCt) as an HTP crystallisation platform for small molecules.^19^ ENaCt employs liquid handling robotics to set-up nanolitre scale crystallisation experiments in 96-well plate format, in which solutions of test molecules in organic solvents are encapsulated in inert oils to mediate the rate of sample concentration, through both evaporation and/or diffusion into the oil. This facilitates large scale, parallel, crystallisation screening using a few micrograms of material per experiment, allowing hundreds of experiments to be undertaken with only milligrams of substrate. Over a number of days, slow growth of suitable single crystals occurs, and crystallisation outcomes can be assessed by SCXRD, resulting in full structural characterisation of crystal forms obtained. ENaCt has proven extremely successful in exploring crystallisation space for single-component systems, allowing for the study of a wide range of small molecule classes as well as hydrogen-bonded organic frameworks (HOFs),^20^ but has not been applied previously to multi-component crystal systems.

Our aim was therefore to develop HTP ENaCt protocols suitable for extensive exploration of the co-crystallisation space for small molecules. We anticipated that ENaCt would enable 1000s of co-crystallisation experiments to be performed in parallel, with minimal sample requirements, generating co-crystals suitable for SCXRD analysis. This approach would provide a step-change in co-crystal screening allowing rapid access to new, structurally characterised, co-crystals.

Herein, we report our development of new approaches for co-crystallisation *via* ENaCt, and their application to co-crystal discovery. Following a demonstration of exhaustive co-crystal screening for a series of binary co-crystals, allowing access to all eighteen of the possible binary combinations of three molecules with six different co-formers, we have expanded our approach to encompass the more challenging problem of accessing higher-order co-crystals (HOCs), with the subsequent discovery of eight new ternary and four new quaternary HOCs.

## Results and discussion

### Binary co-crystals

We commenced our study by focussing on the development of HTP ENaCt methods for the discovery of binary co-crystals, as these systems are of most interest in industrial applications for the formulation of APIs.

**Fig. 1:**
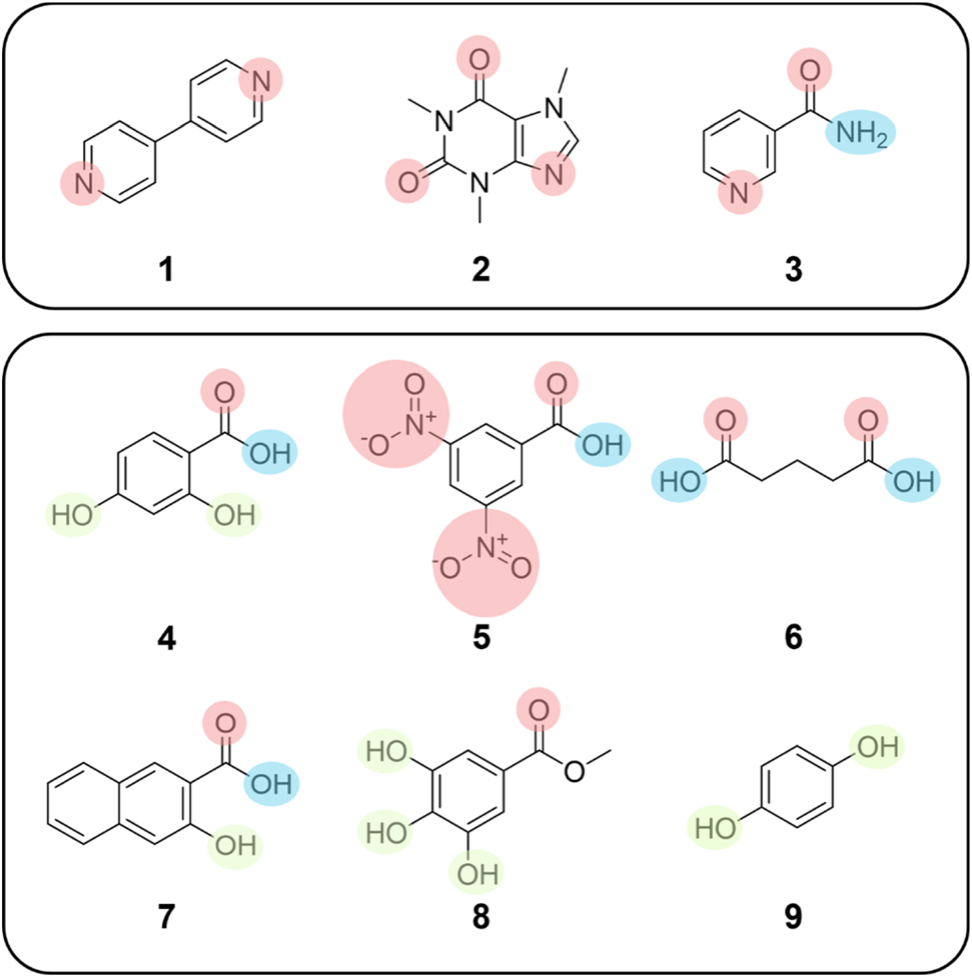
Substrates and co-formers chosen for binary co-crystal screening, showing H-bond acceptor sites (red), H-bond donor sites (blue), and H-bond donor/acceptor sites (green).

For our initial co-crystallisation experiments we selected 4,4′-bipyridine 1, caffeine 2 and nicotinamide 3 as test substrates. These molecules were chosen as a test set as their co-crystallisation has been studied by multiple groups, thus the experimental landscape is well known and we hypothesised that any ‘missing’ combinations with simple co-formers are likely to have been previously attempted by classical methods, albeit not successfully.^9*b*,21^ All three molecules are also aromatic, of similar molecular size, and contain one or more basic sp^2^ hybridised nitrogen atoms capable of acting as H-bond acceptors. A set of six commonly used co-formers was also chosen, 2,4-dihydroxybenzoic acid 4, 3,5-dinitrobenzoic acid 5, glutaric acid 6, 3-hydroxy-2-naphthoic acid 7, methyl gallate 8, and quinol 9, all of which contained complementary functional groups capable of acting as H-bond donors, whilst also representing a range of different structural motifs (Fig. 1). Of the eighteen possible binary combinations of substrate and co-former, twelve were known in the literature (ESI, S4.5†). All eighteen combinations of substrates and co-formers were examined using the CSD Molecular Complementarity Tool (MCT), to assess the likelihood of co-crystal formation through analysis of five key molecular descriptors.^22^ For substrate/co-former combinations known to form binary co-crystals, as well six previously unknown co-crystals, MCT gave uniformly high hit rates, suggesting that all combinations should be accessible.

Following preliminary solubility testing, the three substrates and six co-formers were independently crystallised as single component systems over 14 days using a standardised ENaCt screening protocol (288 experiments/3× 96-well plates, 12 solvents, 4 encapsulating oils (ESI, S3.2†)). This was to assist in both co-crystallisation solvent selection, and co-crystal identification during subsequent co-crystallisation screening experiments through comparative unit cell analysis. Crystals suitable for SCXRD were obtained for all six co-formers and two substrates, 4,4′-bipyridine 1 and nicotinamide 3, in each case matching known crystal forms (ESI, S4.3†).

Based on these results, we selected four solvents (MeOH, DMF, MeNO_2_ and 1,4-dioxane) to be used in subsequent co-crystal screening, chosen as both substrates and co-formers showed good solubility therein and represented a range of common solvent properties (ESI, S3.1†). In each case, stock solutions of both substrate and co-former were prepared near to the solubility limit for each compound/solvent combination.

ENaCt co-crystallisation experiments employed 200 nL of each of the four encapsulation oils dispensed across a 96-well glass plate, and 150 nL of each test solution, containing both substrate and co-former in a single solvent. Following set-up, plates were sealed with a glass cover slip and left for 14 days. Substrate/co-former solutions were examined in three different volume ratios (2 : 1, 1 : 1 and 1 : 2).

Note that inclusion of crystallisation experiments in which the ratio of substrate to co-formers is varied is important, as a pair of molecules can form multiple co-crystal forms with different substrate/co-former stoichiometries, and the stoichiometry of the co-crystal obtained does not necessarily match with the stoichiometry of the solution from which it was formed.^23^

ENaCt screening of all eighteen substrate/co-former combinations, with four different solvents, four different encapsulating oils (as well as no-oil controls) and three different substrate/co-former ratios, resulted in a total of 3456 individual crystallisation experiments including replicates, each employing a few micrograms of material, encompassing 1080 different crystallisation conditions. Through the use of HTP liquid handling robotics, targeted crystallisation screening experiments can be set up in a short space of time, taking less than an hour to prepare for each substrate/co-former system.

**Fig. 2:**
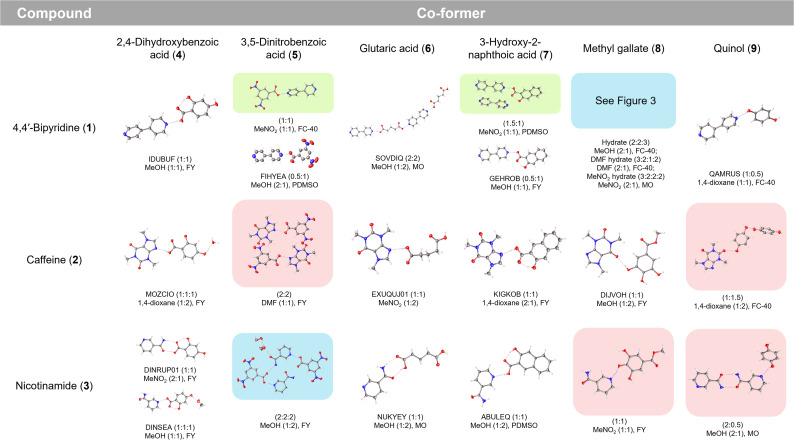
Binary co-crystals accessed *via* ENaCt screening, including previously known co-crystals (white), new co-crystals (red), new co-crystal solvates, hydrates and solvate/hydrates (blue) and new co-crystals showing altered stoichiometry (green). CSD refcode (for known co-crystals), crystal composition ((A/B) : (solvent) : (water)), ENaCt crystallisation conditions (solvent, v/v ratio, oil) shown (MO = mineral oil, PDMSO = poly(dimethylsiloxane), FY = Fomblin YR-1800, and FC-40: Fluorinert FC-40).

After 14 days, all experiments within the 96-well plates were inspected by cross-polarised optical microscopy to search for successful crystallisation outcomes. Crystals were extracted from the 96-well plates and examined by SCXRD. A number of single-component crystals of either substrate or co-former were identified, which were differentiated from target co-crystals by both crystal morphology and unit-cell data (ESI, S4.4.1†). For every one of the eighteen binary combinations of substrate and co-former, suitable co-crystals were obtained, and complete structural analysis was undertaken by SCXRD. Alongside the 13 known co-crystals obtained, we report the discovery of 10 new binary co-crystals, including examples of all 6 of the previously unreported substrate/co-former combinations, as well as new co-crystal solvates, hydrates, solvate/hydrates and stoichiometries (Fig. 2).

**Fig. 3:**
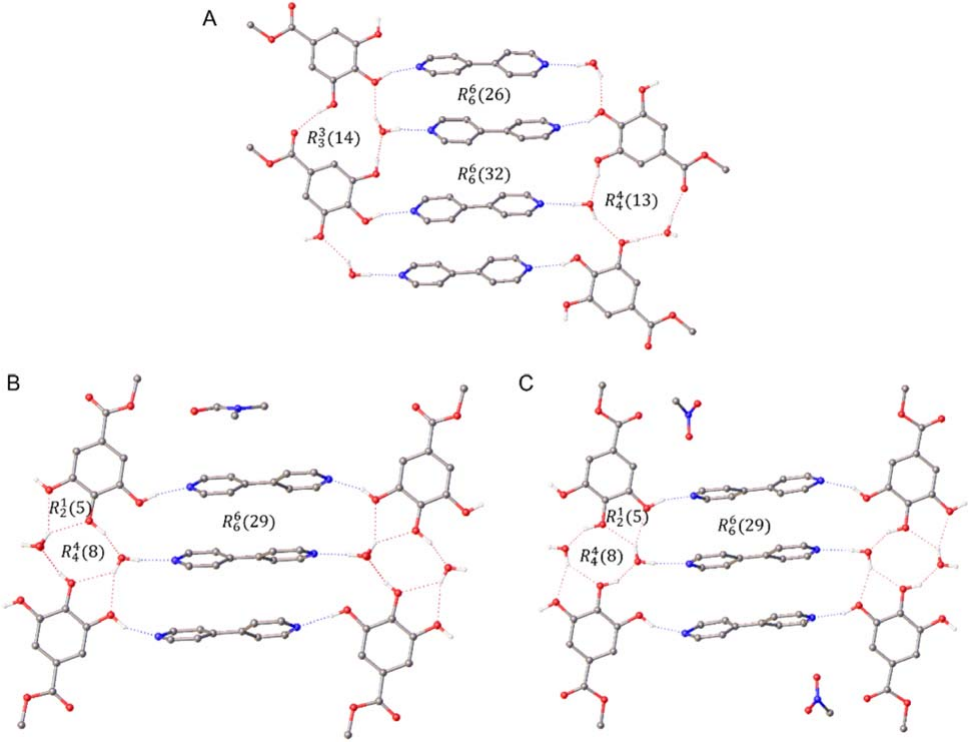
SCXRD structures showing H-bonding networks in the 4,4′-bipyridine : methyl gallate co-crystal series. (A) 2 : 2 trihydrate, (B) 3 : 2 DMF solvate dihydrate and (C) 3 : 2 MeNO_2_ disolvate dihydrate.

Of the new binary co-crystals obtained, the previously unreported combination of 4,4′-bipyridine 1 : methyl gallate 8 gave three related co-crystals, a 2 : 2 trihydrate, a 3 : 2 DMF solvate dihydrate and a 3 : 2 MeNO_2_ disolvate dihydrate (ESI, S4.4.1†). The structure of the 4,4′-bipyridine 1 : methyl gallate 8 2 : 2 trihydrate co-crystal is composed of 4,4′-bipyridine molecules held together by face-to-face π-interactions, where the nitrogen atoms form H-bonds to water and methyl gallate 8, themselves forming a linear H-bonded network co-planar to the crystallographic (−120) plane (Fig. 3A). Whilst the 3 : 2 DMF solvate dihydrate and the 3 : 2 MeNO_2_ disolvate dihydrate show a related packing arrangement, with a trimeric π-stacked 4,4′-bipyridine unit H-bonded to a linear H-bond networked chain of water and methyl gallate molecules (Fig. 3B and C).

Two new 4,4′-bipyridine 1 co-crystals were also observed with both 3,5-dinitrobenzoic acid 5 and with 3-hydroxy-2-naphthoic acid 7, in which the respective molecular stoichiometries (1 : 1 and 1.5 : 1, substrate : co-former) differed from those previously reported.

ENaCt co-crystallisation screening with caffeine 2 gave new co-crystals for both previously unreported small molecule/co-former pairings with 3,5-dinitrobenzoic acid 5 and quinol 9, which were formed with 2 : 2 and 1.5 : 1 stoichiometries respectively.

Finally, the previously unreported combinations of nicotinamide 3 with methyl gallate 8, quinol 9 and 3,5-dinitrobenzoic acid 5 were all obtained, formed with 1 : 1, 2 : 0.5 and 2 : 2 (MeOH disolvate) stoichiometries respectively.

Due to our success in accessing the target of binary co-crystals for all the possible 18 substrate/co-former combinations, *via* rapid, small scale, parallelised ENaCt co-crystallisation experiments, we next turned our attention to the much more challenging area of multi-component co-crystals which contain two or more co-formers.

### Higher order co-crystals – ternary co-crystals

Although the primary focus of pharmaceutical co-crystal research revolves around the discovery of binary co-crystals, there is emerging interest in accessing multi-component or higher-order co-crystals (HOCs), in which three or more different small molecules are combined in a crystal, in stoichiometric ratios. Relatively few ternary and quaternary co-crystal are known, with around 150 ternary co-crystals and around 50 quaternary co-crystals reported prior to this work.^24,25^ The comparatively small number of reported HOCs is due to added difficulties of exploring an increasingly complex experimental solid-state landscape, involving three or more molecular components alongside other experimental variables. Additionally, whilst crystal structure prediction (CSP) tools have become increasingly widespread for the rational design of binary co-crystals,^16*a*,18*b*,26^ such a computational toolkit for the construction of HOCs is not well-established. Current HOC design strategies therefore mainly rely on the use of empirical crystal engineering approaches, based on supramolecular synthons,^15*a*,27^ including synthon hierarchy,^28^ shape-size mimicry^29^ in combination with a long-range synthon Aufbau module (LSAM) strategy,^30^ structural inequivalence,^31^ and combinatorial synthesis.^32^

We envisaged that the combination of high-throughput ENaCt co-crystal screening with suitable crystal engineering strategies would enable us to efficiently access new ternary HOCs. To this end we designed a HOC ENaCt co-crystal screen in which the co-crystallisation of three different molecular components would be examined, with new ternary co-crystals to be accessed *via* a shape-size mimicry replacement strategy.

For ternary co-crystal screening, solutions of each of the molecules of interest were prepared in each of four different solvents (MeOH, DMF, MeNO_2_ and 1,4-dioxane). ENaCt experiments were undertaken using 200 nL of each of the four oils, and 140–150 nL of a solution containing all three components, in seven different v/v ratios (2 : 1 : 1, 1 : 2 : 1, 1 : 1 : 2, 2 : 2 : 1, 2 : 1 : 2, 1 : 2 : 2 (140 nL), and 1 : 1 : 1 (150 nL); ESI, S3.2.3†).

**Fig. 4:**
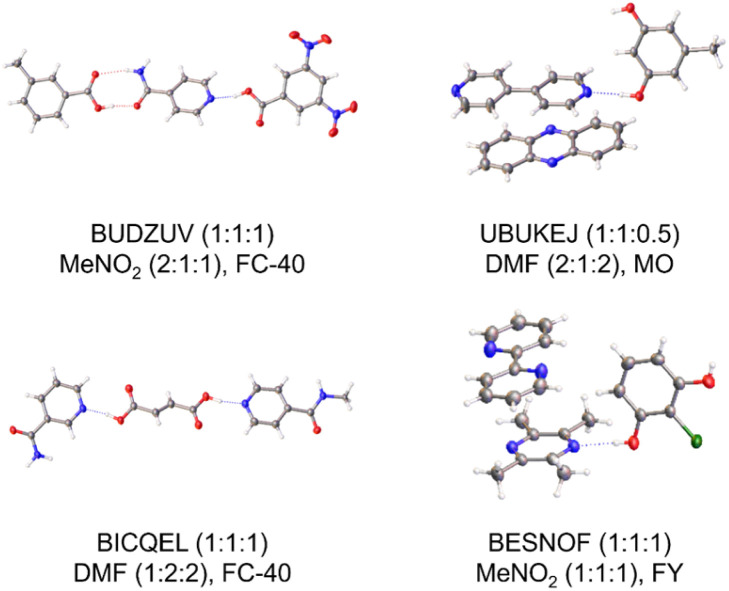
Previously known ternary co-crystals obtained *via* HOC ENaCt screening. CSD refcode, crystal composition (A/B/C), and ENaCt crystallisation conditions (solvent, v/v/v ratio, oil) shown (MO = mineral oil, PDMSO = poly(dimethylsiloxane), FY = Fomblin YR-1800, and FC-40: Fluorinert FC-40).

To validate our methodology, 4 known ternary co-crystals were targeted. Following 288 ENaCt experiments covering 140 crystallisation conditions per target system, for a total of 1152 crystallisations, all four of the desired ternary co-crystals were accessed, and structures obtained by SCXRD. This included toluic acid 10 : isonicotinamide 11 : 3,5-dinitrobenzoic acid 5,^24*b*^ 4,4′-bipyridine 1 : orcinol 12 : phenazine 13,^29^ nicotinamide 3 : fumaric acid 14 : isoniazid 15,^33^ and tetramethylpyrazine 16 : 2,2′-bipyridine 17 : 2-chlororesorcinol 18 (Fig. 4).^34^

**Fig. 5:**
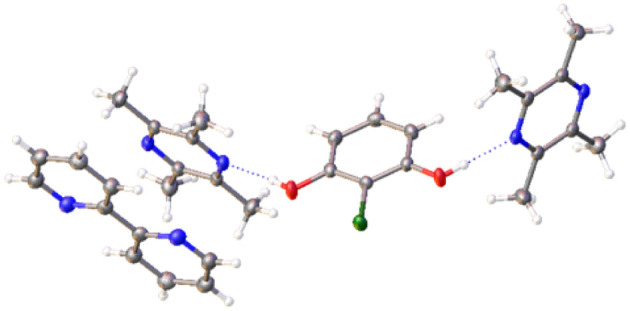
Serendipitous discovery of a new ternary co-crystal tetramethylpyrazine : 2,2′-bipyridine : 2-chlororesorcinol (1 : 0.5 : 1) *via* HOC ENaCt screening.

As a by-product of our search for known ternary co-crystals, a number of other systems were identified by SCXRD, including single component crystals as well as binary and ternary co-crystals (ESI, S4.4.1†). Of particular note was the serendipitous discovery of a new (1 : 0.5 : 1) ternary co-crystal of tetramethylpyrazine 16 : 2,2′-bipyridine 17 : 2-chlororesorcinol 18, showing an alternative stoichiometry to that previously known (Fig. 5).

Additionally, during the search for a 4,4′-bipyridine 1 : orcinol 12 : phenazine 13 ternary system, serendipitous discovery of two binary co-crystals were obtained, a known 1.5 : 1 4,4′-bipyridine 1 : orcinol 12 co-crystal and a new 1 : 1 : 0.5 orcinol 12 : phenazine 13 : 1,4-dioxane co-crystal solvate (ESI, S4.4.2†).

**Fig. 6:**
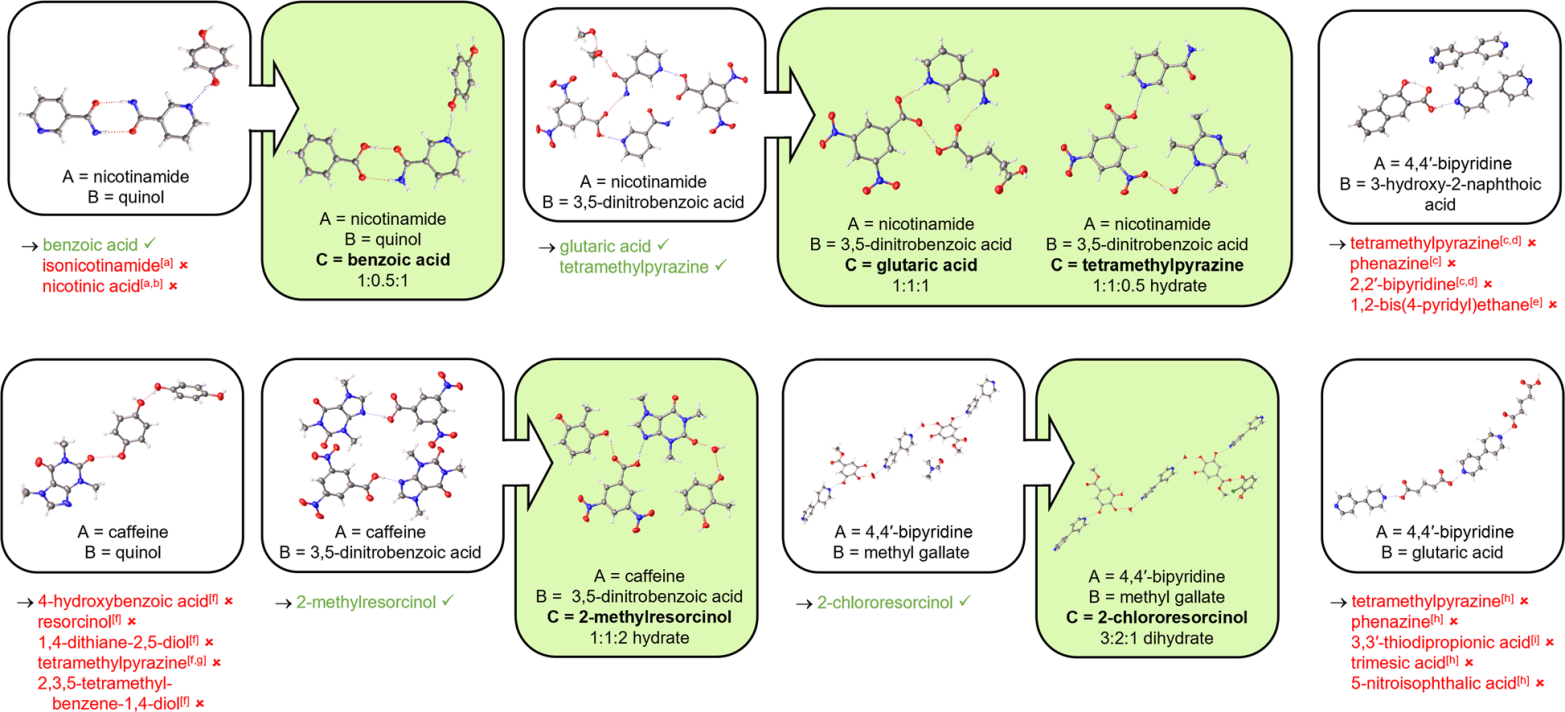
HOC ENaCt screening outcomes for ternary co-crystal discovery *via* molecular replacement with shape-size mimics from the corresponding binary co-crystals, including parent binary systems (white) and new ternary co-crystals obtained (green). Crystal composition ratio (A/B/C) shown. Co-formers screened are given below each parent binary system, with successful (green) and unsuccessful (red) co-formers highlighted. Additional binary co-crystals obtained are indicated: ^*a*^nicotinamide : quinol (2 : 0.5) polymorph I, ^*b*^nicotinamide : quinol (2 : 0.5) polymorph II, ^*c*^4,4′-bipyridine : 3-hydroxy-2-naphthoic acid (0.5 : 1), ^*d*^4,4′-bipyridine : 3-hydroxy-2-naphthoic acid (1.5 : 1), ^*e*^3-hydroxy-2-naphthoic acid : 1,2-bis(4-pyridyl)ethane (1 : 0.5), ^*f*^caffeine : quinol (1 : 1.5), ^*g*^quinol : tetramethylpyrazine (0.5 : 0.5), ^*h*^4,4′-bipyridine : glutaric acid (2 : 2), ^*i*^4,4′-bipyridine : 3,3′-thiodipropionoic acid (0.5 : 0.5).

Our HOC ENaCt screening was next applied to the discovery of new ternary forms. Of our previously investigated binary co-crystal systems, seven contained one molecular species present in two distinct crystallographic environments. These crystal systems were selected as templates for new ternary co-crystals, in which we aimed to replace one of these molecules within the crystal with a suitable isostere, using shape-size mimicry. Structural isosteres were selected manually based on similarities in size, shape, functional group, and functional group orientation. Thus, applying our previously described ternary HOC ENaCt screening approach to these chosen systems, we were successful in designing and accessing 5 new ternary co-crystals in which at least one molecular component of a binary system had been replaced with a newly introduced co-former molecule (Fig. 6). Note known single component crystals and binary co-crystals were also identified (ESI, S4.4.2†).

Our first challenge was to design new ternary co-crystals based on nicotinamide 3. Starting with the co-crystal of nicotinamide 3 : quinol 9 (2 : 0.5) as a template, we screened benzoic acid 19, isonicotinamide 11 and nicotinic acid 20 as shape-size mimics for the replacement of a nicotinamide molecule within the crystal structure (ESI, S4.4.2†). Only co-crystallisation with benzoic acid 19 proved successful, forming a ternary co-crystal in which the newly introduced benzoic acid 19 moiety formed an H-bonded dimer with nicotinamide 3, mimicking the nicotinamide to nicotinamide H-bonded dimer in the parent binary system. In addition, attempted co-crystallisations of nicotinamide 3, quinol 9 and nicotinic acid 20 gave no ternary co-crystals, but did result in the serendipitous formation of a novel co-crystal polymorph of nicotinamide 3 : quinol 9 (2 : 0.5). We were also able to build on the nicotinamide 3 : 3,5-dinitrobenzoic acid 5 binary co-crystal, to form both a new 1 : 1 : 1 ternary co-crystal containing nicotinamide 3, 3,5-dinitrobenzoic acid 5 and glutaric acid 6, and a new 1 : 1 : 0.5 ternary co-crystal hydrate containing nicotinamide 3, 3,5-dinitrobenzoic acid 5 and tetramethylpyrazine 16. In both cases the ternary co-crystals maintained the key nicotinamide 3 to 3,5-dinitrobenzoic acid 5 N–H–O H-bond.

Attempts to generate ternary co-crystals starting from either 4,4′-bipyridine 1 : 3-hydroxy-2-naphthoic acid 7 or 4,4′-bipyridine 1 : glutaric acid 6 proved challenging, the former providing only the previously discussed binary co-crystals (1.5 : 1 and 0.5 : 1 stoichiometries), as well as a novel binary co-crystal 3-hydroxy-2-naphthoic acid 7 : 1,2-bis(4-pyridyl)ethane 21, (1 : 0.5), whilst the later mainly gave the parent 4,4′-bipyridine 1 : glutaric acid 6 co-crystal (2 : 2), as well as a known 4,4′-bipyridine 1 : 3,3′-thiodipropionic acid 22 (0.5 : 0.5) co-crystal. The 4,4′-bipyridine 1 : methyl gallate 8 system did however allow access to a new 4,4′-bipyridine 1 : methyl gallate 8 : 2-chlororesorcinol 18 (3 : 2 : 1) dihydrate, which maintained the core 4,4′-bipyridine/methyl gallate/water, H-bonded chain structure.

Finally, starting from caffeine 2 : quinol 9 (1 : 1.5), attempts to introduce suitable shape-size mimics for quinol proved unsuccessful, with no new ternary systems obtained. The parent binary co-crystal was observed in almost all cases, with the one exception being the observation of a known binary co-crystal of quinol 9 : tetramethylpyrazine 16 (0.5 : 0.5). The caffeine 2 : 3,5-dinitrobenzoic acid 5 co-crystal proved more successful, resulting in a new ternary co-crystal hydrate of caffeine 2 : 3,5-dinitrobenzoic acid 5 : 2-methylresorcinol 23 (1 : 1 : 2).

### Higher order co-crystals – quaternary co-crystals

Following the highly successful generation of new ternary co-crystals *via* ENaCt, next we decided to extend our investigation to encompass more challenging HOCs, quaternary co-crystals.

Based on our ternary screening approach, for quaternary co-crystal screening by ENaCt four different solvents were again used (MeOH, DMF, MeNO_2_ and 1,4-dioxane) to prepare near saturated solutions of each of the four molecules required for each system. 200 nL of each of the four oils were distributed across standard 96-well glass plates (ESI S3.2.4†), followed by the sequential pick-up of each component solution in a simple 1 : 1 : 1 : 1 volume ratio into the dispensing needles, for a total volume of 140 nL. These solutions were then injected into the pre-prepared oil containing 96-well plates, plates were sealed with a glass coverslip and stored for 2 weeks before being assessed by polarising optical microscopy.

**Fig. 7:**
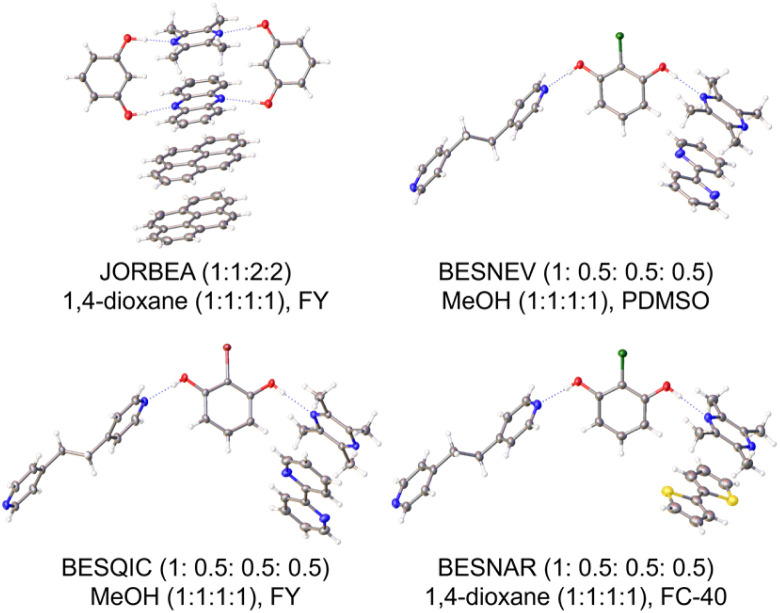
Previously known quaternary co-crystals obtained *via* HOC ENaCt screening. CSD refcode, crystal composition ratio (A/B/C/D), and ENaCt crystallisation conditions (solvent, v/v/v/v ratio, oil) shown (MO = mineral oil, PDMSO = poly(dimethylsiloxane), FY = Fomblin YR-1800, and FC-40: Fluorinert FC-40).

Four known quaternary co-crystal systems were initially targeted to validate the use of ENaCt in such a demanding HOC formation experiment, namely tetramethylpyrazine 16 : phenazine 13 : pyrene 24 : resorcinol 25,^25*a*^ 2-chlororesorcinol 18 : tetramethylpyrazine 16 : 2,2′-bipyridine 17 : 1,2-bis(4-pyridyl)ethane 21,^34^ 2-bromoresorcinol 26 : tetramethylpyrazine 16 : 2,2′-bipyridine 17 : 1,2-bis(4-pyridyl)ethane 21,^34^ and 2-chlororesorcinol 18: tetramethylpyrazine 16 : 2,2′-bithiophene 27 : 1,2-bis(4-pyridyl)ethane 21.^34^ In all four cases suitable quaternary co-crystals were grown, and structures obtained by SCXRD (Fig. 7), alongside the observation of known lower order crystals (ESI, 4.4.3†).

Due to the success of applying ENaCt to the formation of known quaternary co-crystals, next we decided to target novel quaternaries *via* this method. To this end we selected several of our previously accessed ternary co-crystal systems (caffeine 2 : 3,5-dinitrobenzoic acid 5 : 2-methylresorcinol 23 hydrate, methyl gallate 8 : 4,4′-bipyridine 1 : 2-chlororesorcinol 18 hydrate, and 2-chlororesorcinol 18 : tetramethylpyrazine 16 : 2,2′-bipyridine 17), in which at least one component was present in the structure in more than one distinct chemical environment, and engaged in a shape-size mimic screening approach to access new quaternary HOCs, based on our previous success with novel ternary systems.

**Fig. 8:**
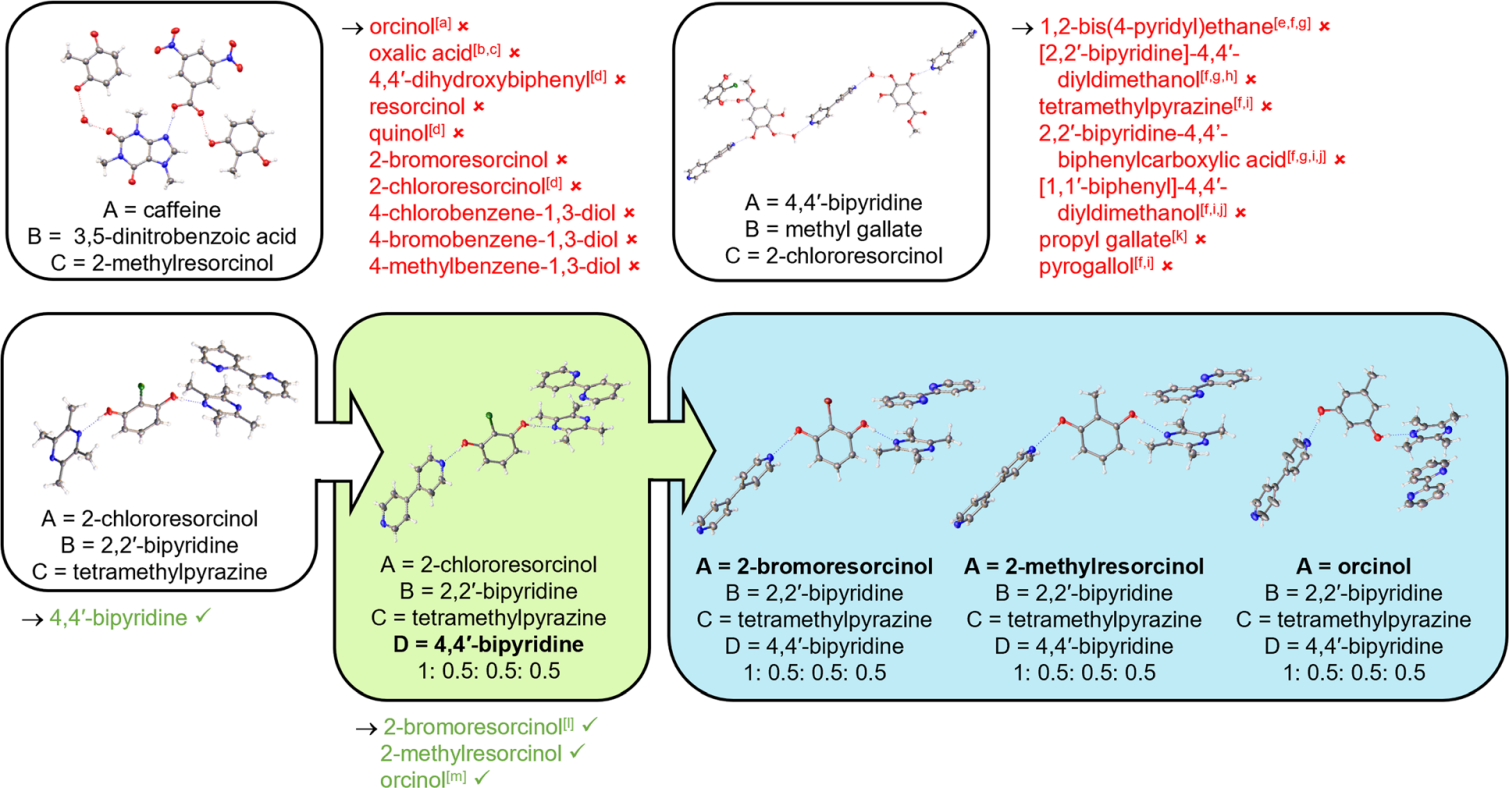
HOC ENaCt screening outcomes for quaternary co-crystal discovery *via* molecular replacement with shape-size mimics from the corresponding ternary co-crystals, including parent ternary systems (white) and new quaternary co-crystals obtained (green). Crystal composition ratio (A/B/C/D) shown. Co-formers screened are given below each parent ternary system, with successful (green) and unsuccessful (red) co-formers highlighted. Additional binary and ternary co-crystals obtained are indicated: ^*a*^orcinol : 3,5-dinitrobenzoic acid (4 : 4) hydrate, ^*b*^caffeine : oxalic acid (1 : 0.5), ^*c*^caffeine : 2-methylresorcinol : oxalic acid (1 : 1:0.5), ^*d*^caffeine : 3,5-dinitrobenzoic acid : 2-methylresorcinol (1 : 1 : 2) hydrate, ^*e*^4,4′-bipyridine : methyl gallate : 2-chlororesorcinol (3 : 1 : 1), ^*f*^4,4′-bipyridine : methyl gallate (3 : 2) MeNO_2_ disolvate dihydrate, ^*g*^4,4′-bipyridine : methyl gallate : 2-chlororesorcinol (3 : 2 : 1) dihydrate, ^*h*^methyl gallate : [2,2′-bipyridine]-4,4′-diyldimethanol (1 : 0.5), ^*i*^4,4′-bipyridine : methyl gallate (2 : 2) trihydrate, ^*j*^4,4′-bipyridine : methyl gallate (3 : 2) DMF solvate dihydrate, ^*k*^4,4′-bipyridine : propyl gallate (1 : 1), ^*l*^2-bromoresorcinol : 4,4′-bipyridine (2 : 3), ^*m*^orcinol : 4,4′-bipyridine (1 : 1.5).

**Fig. 9:**
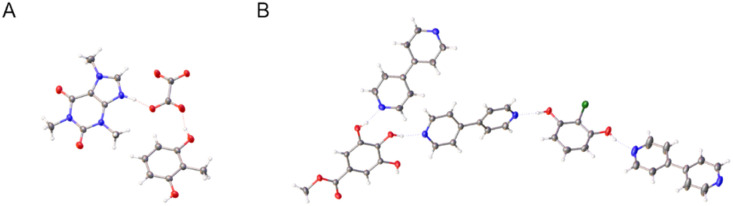
Serendipitous discovery of new ternary co-crystals (A) caffeine : 2-methylresorcinol : oxalic acid (1 : 1 : 1) and (B) 4,4′-bipyridine : methyl gallate : 2-chlororesorcinol (3 : 1 : 1).

Starting with the ternary co-crystal system caffeine 2 : 3,5-dinitrobenzoic acid 5 : 2-methylresorcinol 23 hydrate, we targeted the replacement of one of the 2-methylresorcinol 23 moieties, with quinol 9, orcinol 12, resorcinol 25, oxalic acid 28, 4,4′-dihydroxybiphenyl 29, 2-bromoresorcinol 26, 2-chlororesorcinol 18, 4-chlorobenzene-1,3-diol 30, 4-bromobenzene-1,3-diol 31 and 4-methylbenzene-1,3-diol 32 (Fig. 8) (ESI, S4.4.3†). Despite the serendipitous discovery of a new ternary co-crystal of caffeine 2 : 2-methylresorcinol 23 : oxalic acid 28 (1 : 1 : 0.5) (Fig. 9A), no quaternary co-crystals were obtained.

The second quaternary system targeted was based on the ternary co-crystal hydrate of methyl gallate 8, 4,4′-bipyridine 1 and 2-chlororesorcinol 18. We initially examined the replacement of the 4,4′-bipyridine/H_2_O complex, thus undertaking ENaCt co-crystallisations with methyl gallate 8, 4,4′-bipyridine 1 and 2-chlororesorcinol 18 in combination with 1,2-bis(4-pyridyl)ethane 21, [2,2′-bipyridine]-4,4′-diyldimethanol 33, [2,2′-bipyridine]-5,5′-dicarboxylic acid 34, 4,4′-biphenyldimethanol 35, 4,4′-dihydroxybiphenyl 29, and 4,4′-biphenyldicarboxylic acid 36 (ESI, S4.4.3†). Unfortunately, none of these experiments yielded new quaternary systems, although three previously observed binary and one previously observed ternary co-crystal were identified, alongside a new binary co-crystal of methyl gallate 8 : [2,2′-bipyridine]-4,4′-diyldimethanol 33 (1 : 0.5) and a new ternary co-crystal of 4,4′-bipyridine 1 : methyl gallate 8 : 2-chlororesorcinol 18 (3 : 1 : 1) (Fig. 9B). Additional experiments were undertaken to substitute a methyl gallate moiety, with either propyl gallate 37 or pyrogallol 38. Again, none of the desired quaternary systems were obtained, but a new binary co-crystal was observed, 4,4′-bipyridine 1: propyl gallate 37 (1 : 1) (ESI, S4.4.3†).

Finally, starting from 2-chlororesorcinol 18 : tetramethylpyrazine 16 : 2,2′-bipyridine 17, we targeted the substitution of one tetramethylpyrazine 16. 4,4′-Bipyridine 1 was chosen as it displays a similar antipodal arrangement of sp^2^ nitrogen H-bond acceptor sites. Pleasingly, ENaCt co-crystallisation screening of 2-chlororesorcinol 18, tetramethylpyrazine 16, 2,2′-bipyridine 17 and 4,4′-bipyridine 1 resulted in the discovery of a novel (1 : 0.5 : 0.5 : 0.5) quaternary co-crystal. The newly formed HOC was isostructural with the parent system, with 4,4′-bipyridine 1 replacing one tetramethylpyrazine 16 (Fig. 8).

In previous studies, new HOCs have been accessed through the replacement of 2-chlororesorcinol 18, employing shape-size mimicry with structurally related diols.^29,31,34^ Based on this, our goal was therefore to generate a new family of quaternary co-crystals using our newly discovered quaternary co-crystal as the parent system. Thus, experiments were setup following our standard quaternary co-crystal protocols (ESI, S3.2.4†), in which 2-chlororesorcinol 18 was swapped for 2-bromoresorcinol 26, 2-methylresorcinol 23, and orcinol 12.

Alongside the serendipitous discovery of a new binary co-crystal containing 4,4′-bipyridine 1 : 2-bromoresorcinol 26 (ESI, S4.4.3†), we also obtained three novel quaternary co-crystals (Fig. 8). The new quaternary (1 : 0.5 : 0.5 : 0.5) co-crystals were all isomorphous with the parent system, in each case 2-chlororesorcinol 18 being swapped for the newly introduced diol moiety, 2-bromoresorcinol 26, 2-methylresorcinol 23, or orcinol 12.

Thus, the discovery of a family of four isostructural quaternary co-crystals demonstrates the applicability of HTP methods to the solution of complex crystallisation challenges.

## Conclusions

The HTP nature of our herein developed ENaCt co-crystallisation method has allowed us to carry out 13 056 individual crystallisations, using only 0.25–5 µg of each substrate per experiment. Targeted attempts to access binary co-crystals, as well as ternary and quaternary HOCs, gave excellent success rates for both known and new systems, with the co-crystals generated being suitable for direct SCXRD analysis. ENaCt co-crystallisation screening has thus allowed rapid access to 54 different co-crystals, including 17 new binary, 8 new ternary and 4 new quaternary co-crystals. The rapid, material efficient, HTP generation of such a diverse array of co-crystals clearly demonstrates the power and promise of this methodology for future use in both fundamental research and in the development of pharmaceutically relevant co-crystal systems, heralding a new era of complex, multi-component solid-state experimental space exploration for co-crystal discovery.

## Data availability

The data supporting this article have been included as part of the ESI.† CCDC 2372065–2372114, 2428848–2428851 contain the supplementary crystallographic data for this paper. These data can be obtained free of charge from The Cambridge Crystallographic Data Centre *via*https://www.ccdc.cam.ac.uk/structure.

## Author contributions

The manuscript was written through contributions of all authors. JPM carried out crystallisation experiments, SCXRD analysis, and produced the draft manuscript. PAC, JFMcC, MRP and MJH provided supervision and advice. MRP assisted with SCXRD analysis. MJH produced the final manuscript. All authors have given approval for the final version of the manuscript.

## Conflicts of interest

JPM, MJH and MRP and authors of a patent application related to this work.

## Supplementary Material

SC-OLF-D4SC07556K-s001

SC-OLF-D4SC07556K-s002
