# Supplementary material for: High-throughput encapsulated nanodroplet screening for accelerated co-crystal discovery
Source: Chem Sci. 2025 Apr 22;16(22):9843–53. doi: 10.1039/d4sc07556k (PMC12044422; doi:10.1039/d4sc07556k)
Supplement: SC-016-D4SC07556K-s001 [file SC-016-D4SC07556K-s001.pdf]

Supplementary Information (SI) for Chemical Science.
This journal is © The Royal Society of Chemistry 2025

High-Throughput Encapsulated Nanodroplet Screening for Accelerated Co-Crystal Discovery

Jessica P. Metherall,[a] Philip A. Corner,[b] James F. McCabe,[b] Michael R. Probert[a] and Michael J. Hall[a]

[a] Chemistry, School of Natural and Environmental Sciences, Newcastle University, Newcastle upon Tyne, UK.

[b] Early Product Development & Manufacturing, Pharmaceutical Sciences, BioPharmaceuticals R&D, AstraZeneca, Macclesfield, UK.

Supporting Information (148 pages)

**S1. General Experimental Information**	S4
**S2. ENaCt Oils and Solvents**	S5
**S3. High-Throughput Crystallisation and Co-Crystallisation Methods (ENaCt)**	S6
S3.1 Stock Solution Preparation	S6
S3.2 Experimental Design and 96-Well Plate Layouts	S12
S3.2.1 Single Component High-Throughput Crystallisation	S12
S3.2.2 Binary High-Throughput Co-Crystallisation	S13
S3.2.3 Ternary High-Throughput Co-Crystallisation	S14
S3.2.4 Quaternary High-Throughput Co-Crystallisation	S16
**S4. High-Throughput Co-Crystallisation Results**	S17
S4.1 Classification of Crystallisation Outcomes by Optical Microscopy	S17
S4.2 Selection of Crystals and Co-Crystals for SCXRD Analysis	S17
S4.3 Results of ENaCt Crystallisation for Single Component Systems	S17
S4.3.1 Crystallisation of Substrates	S17
S4.3.2 Crystallisation of Co-Formers	S17
S4.4 Co-Crystallisation 96-Well Plate Readouts including Classifications and Crystals Selected for SCXRD Analysis	S18
S4.4.1 Binary Co-Crystallisation 96-Well Plate Readouts	S19
S4.4.2 Ternary Co-Crystallisation 96-Well Plate Readouts	S37
S4.4.3 Quaternary Co-Crystallisation 96-Well Plate Readouts	S62
S4.5 Cross-Polarised Optical Microscopy Images of Crystals for which full SCXRD Analysis was Performed	S87
**S5. Crystal Data and Structure Refinement Details**	S94
S5.1 Previously Known Binary Co-Crystals from Binary Co-Crystal Screening	S94
S5.2 New Binary Co-Crystals from Binary Co-Crystal Screening	S107

S5.3 Previously Known Binary Co-Crystals from Ternary and Quaternary Co-Crystal Screening	S117
S5.4 New Binary Co-Crystals from Ternary and Quaternary Co-Crystal Screening	S121
S5.5 Previously Known Ternary Co-Crystals from Ternary Co-Crystal Screening	S128
S5.6 New Ternary Co-Crystals from Ternary Co-Crystal Screening	S132
S5.7 New Ternary Co-Crystals from Quaternary Co-Crystal Screening	S138
S5.8 Previously Known Quaternary Co-Crystals from Quaternary Co-Crystal Screening	S140
S5.9 New Quaternary Co-Crystals from Quaternary Co-Crystal Screening	S144
**S6. References**	S148

S1. General Experimental Information

Crystallisation experiments were completed using a SPT LabTech mosquito® liquid handling robot using SwissSCI Modular LCP 96-well glass plates with a 100 µm spacer and sealed with a glass cover slip.

Visualisation of experiments was carried out with a Nikon SMZ1000 microscope fitted with a cross polariser, and photographs were taken with a GXCAM-U3-5 5.1MP camera.

Upon observation of suitable crystals (grade 4), the relevant wells were opened with the use of a tungsten carbide scriber to remove a small portion of the glass cover slide, and the crystal manipulated using MiTeGen Kapton microtools. Crystals were transferred onto a standard MiTeGen Kapton loop and mounted onto an in-house diffractometer.

Single crystal X-ray diffraction experiments were performed using an in-house diffractometer: XtaLAB Synergy HyPix-Arc 100 diffractometer using copper radiation ($\lambda_{CuK\alpha}$ = 1.54184 Å, equipped with an Oxford Cryosystems Cryostream with data recorded at 150 K or 295 K.

Data were reduced using CrysAlis^{PRO} with SCALE 3 ABSPACK correction implemented.[1] All samples were cooled, and temperature maintained using Oxford Cryosystems Cryostreams.[2]

All structure solution and refinement were completed using the SHELX suite[3,4] of programs via the Olex2 interface.[5]

S2. ENaCt Oils and Solvents

Table S1 ENaCt Oils and Solvents

ENaCt Oils	
PDMSO	Poly(dimethylsiloxane); CAS: 63148-62-9; supplier: Sigma Aldrich
FC-40	Fluorinert FC-40; CAS: 51142-49-5; supplier: Fluorochem
FY	Fomblin YR-1800; CAS: 69991-67-9; supplier: Alfa Aesar
MO	Mineral oil; CAS: 8042-47-5; supplier: Sigma Aldrich
**ENaCt Solvents**	
DMSO	Dimethyl sulfoxide
DMF	Dimethylformamide
MeOH	Methanol
2,2,2-TFE	2,2,2-Trifluoroethanol
Toluene	Toluene
DCE	1,2-Dichloroethane
2-MeTHF	2-Methyltetrahydrofuran
1,4-Dioxane	1,4-Dioxane
EtOAc	Ethyl acetate
MeCN	Acetonitrile
MIBK	4-Methylpentan-2-one
$MeNO_2$	Nitromethane

S3. High-Throughput Crystallisation and Co-Crystallisation Methods

S3.1 Stock Solution Preparation

Stock solutions of the compound and the appropriate co-former were freshly prepared for each crystallisation experiment. Samples were weighed (~2 mg) into screw top vials and dissolved in one of 4 solvents (dimethylformamide (DMF), methanol (MeOH), 1,4-dioxane, and nitromethane ($MeNO_2$), through portion wise solvent addition until a near saturated solution was formed.

Table S2 Stock Solutions

Compound	Solvent	Mass/ mg	Volume/ µL	Conc./ mg mL^{-1}
**1** 4,4′-bipyridine	Dimethylformamide (DMF)	2	12	167
	Methanol (MeOH)	2	12	167
	1,4-Dioxane	2	24	83
	Nitromethane ($MeNO_2$)	2	48	42
**2** caffeine	Dimethylformamide (DMF)	2	96	21
	Methanol (MeOH)	2	96	21
	1,4-Dioxane	2	48	42
	Nitromethane ($MeNO_2$)	2	96	21
**3** nicotinamide	Dimethylformamide (DMF)	2	24	83
	Methanol (MeOH)	2	24	83
	1,4-Dioxane	2	48	42
	Nitromethane ($MeNO_2$)	2	48	42
**4** 2,4-dihydroxybenzoic acid	Dimethylformamide (DMF)	2	48	42
	Methanol (MeOH)	2	12	167
	1,4-Dioxane	2	48	42
	Nitromethane ($MeNO_2$)	2	96	21
**5** 3,5-dinitrobenzoic acid	Dimethylformamide (DMF)	2	48	42
	Methanol (MeOH)	2	48	42
	1,4-Dioxane	2	24	83
	Nitromethane ($MeNO_2$)	2	48	42

**6** glutaric acid	Dimethylformamide (DMF)	2	24	83
	Methanol (MeOH)	2	12	167
	1,4-Dioxane	2	48	42
	Nitromethane ($MeNO_2$)	2	96	21
**7** 3-hydroxy-2-naphthoic acid	Dimethylformamide (DMF)	2	24	83
	Methanol (MeOH)	2	48	42
	1,4-Dioxane	2	48	42
	Nitromethane ($MeNO_2$)	2	96	21
**8** methyl gallate	Dimethylformamide (DMF)	2	24	83
	Methanol (MeOH)	2	24	83
	1,4-Dioxane	2	24	83
	Nitromethane ($MeNO_2$)	2	96	21
**9** quinol	Dimethylformamide (DMF)	2	24	83
	Methanol (MeOH)	2	12	167
	1,4-Dioxane	2	48	42
	Nitromethane ($MeNO_2$)	2	48	42
**10** toluic acid	Dimethylformamide (DMF)	2	12	167
	Methanol (MeOH)	2	12	167
	1,4-Dioxane	2	24	83
	Nitromethane ($MeNO_2$)	2	48	42
**11** isonicotinamide	Dimethylformamide (DMF)	2	12	167
	Methanol (MeOH)	2	24	83
	1,4-Dioxane	2	48	42
	Nitromethane ($MeNO_2$)	2	96	21
**12** orcinol	Dimethylformamide (DMF)	2	48	42
	Methanol (MeOH)	2	48	42
	1,4-Dioxane	2	24	83
	Nitromethane ($MeNO_2$)	2	48	42

**13**	phenazine	Dimethylformamide (DMF)	2	48	42
		Methanol (MeOH)	2	48	42
		1,4-Dioxane	2	24	83
		Nitromethane ($MeNO_2$)	2	24	83
**14**	fumaric acid	Dimethylformamide (DMF)	2	24	83
		Methanol (MeOH)	2	48	42
		1,4-Dioxane	2	48	42
		Nitromethane ($MeNO_2$)	2	96	21
**15**	isoniazid	Dimethylformamide (DMF)	2	12	167
		Methanol (MeOH)	2	12	167
		1,4-Dioxane	2	12	167
		Nitromethane ($MeNO_2$)	2	48	42
**16**	tetramethyl-pyrazine	Dimethylformamide (DMF)	2	24	83
		Methanol (MeOH)	2	12	167
		1,4-Dioxane	2	24	83
		Nitromethane ($MeNO_2$)	2	24	83
**17**	2,2′-bipyridine	Dimethylformamide (DMF)	2	24	83
		Methanol (MeOH)	2	12	167
		1,4-Dioxane	2	24	83
		Nitromethane ($MeNO_2$)	2	48	42
**18**	2-chlororesorciol	Dimethylformamide (DMF)	2	48	42
		Methanol (MeOH)	2	24	83
		1,4-Dioxane	2	24	83
		Nitromethane ($MeNO_2$)	2	24	83
**19**	benzoic acid	Dimethylformamide (DMF)	2	48	42
		Methanol (MeOH)	2	12	167
		1,4-Dioxane	2	48	42
		Nitromethane ($MeNO_2$)	2	96	21

**20**	nicotinic acid	Dimethylformamide (DMF)	2	24	83
		Methanol (MeOH)	2	48	42
		1,4-Dioxane	2	96	21
		Nitromethane ($MeNO_2$)	2	96	21
**21**	1,2-bis(4-pyridyl)ethane	Dimethylformamide (DMF)	2	24	83
		Methanol (MeOH)	2	24	83
		1,4-Dioxane	2	48	42
		Nitromethane ($MeNO_2$)	2	24	83
**22**	3,3′-thiodisopropanoic acid	Dimethylformamide (DMF)	2	24	83
		Methanol (MeOH)	2	12	167
		1,4-Dioxane	2	96	21
		Nitromethane ($MeNO_2$)	2	96	21
**23**	2-methylresorcinol	Dimethylformamide (DMF)	2	48	42
		Methanol (MeOH)	2	12	167
		1,4-Dioxane	2	24	83
		Nitromethane ($MeNO_2$)	2	24	83
**24**	pyrene	Dimethylformamide (DMF)	2	24	83
		Methanol (MeOH)	2	48	42
		1,4-Dioxane	2	24	83
		Nitromethane ($MeNO_2$)	2	24	83
**25**	resorcinol	Dimethylformamide (DMF)	2	12	167
		Methanol (MeOH)	2	12	167
		1,4-Dioxane	2	24	83
		Nitromethane ($MeNO_2$)	2	24	83
**26**	2-bromoresorcinol	Dimethylformamide (DMF)	2	12	167
		Methanol (MeOH)	2	24	83
		1,4-Dioxane	2	12	167
		Nitromethane ($MeNO_2$)	2	12	167

**27**	2,2′-bithiophene	Dimethylformamide (DMF)	2	24	83
		Methanol (MeOH)	2	12	167
		1,4-Dioxane	2	24	83
		Nitromethane ($MeNO_2$)	2	48	42
**28**	oxalic acid	Dimethylformamide (DMF)	2	24	83
		Methanol (MeOH)	2	24	83
		1,4-Dioxane	2	24	83
		Nitromethane ($MeNO_2$)	2	96	21
**29**	4,4′-dihydroxybiphenyl	Dimethylformamide (DMF)	2	48	42
		Methanol (MeOH)	2	24	83
		1,4-Dioxane	2	48	42
		Nitromethane ($MeNO_2$)	2	96	21
**30**	4-chlorobenzene-1,3-diol	Dimethylformamide (DMF)	2	24	83
		Methanol (MeOH)	2	24	83
		1,4-Dioxane	2	96	21
		Nitromethane ($MeNO_2$)	2	48	42
**31**	4-bromobenzene-1,3-diol	Dimethylformamide (DMF)	2	24	83
		Methanol (MeOH)	2	24	83
		1,4-Dioxane	2	48	42
		Nitromethane ($MeNO_2$)	2	24	83
**32**	4-methylbenzene-1,3-diol	Dimethylformamide (DMF)	2	48	42
		Methanol (MeOH)	2	48	42
		1,4-Dioxane	2	96	21
		Nitromethane ($MeNO_2$)	2	48	42
**33**	[2,2′-bipyridine]-4,4-′diyldimethanol	Dimethylformamide (DMF)	2	24	83
		Methanol (MeOH)	2	48	42
		1,4-Dioxane	2	48	42
		Nitromethane ($MeNO_2$)	2	96	21

**34**	[2,2′-bipyridine]-5,5′-dicarboxylic acid	Dimethylformamide (DMF)	2	24	83
		Methanol (MeOH)	2	24	83
		1,4-Dioxane	2	48	42
		Nitromethane ($MeNO_2$)	2	96	21
**35**	4,4′-biphenyldi-methanol	Dimethylformamide (DMF)	2	24	83
		Methanol (MeOH)	2	48	42
		1,4-Dioxane	2	48	42
		Nitromethane ($MeNO_2$)	2	96	21
**36**	4,4′-biphenyldi-carboxylic acid	Dimethylformamide (DMF)	2	48	42
		Methanol (MeOH)	2	48	42
		1,4-Dioxane	2	48	42
		Nitromethane ($MeNO_2$)	2	96	21
**37**	propyl gallate	Dimethylformamide (DMF)	2	24	83
		Methanol (MeOH)	2	24	83
		1,4-Dioxane	2	24	83
		Nitromethane ($MeNO_2$)	2	48	42
**38**	pyrogallol	Dimethylformamide (DMF)	2	24	83
		Methanol (MeOH)	2	12	167
		1,4-Dioxane	2	24	83
		Nitromethane ($MeNO_2$)	2	48	42

S3.2 Experimental Design and 96-Well Plate Layouts

S3.2.1 Single Component High-Throughput Crystallisation

The following method was used to set up single component crystallisation experiments, utilising 3 plates (SP1, SP2 and SP3) covering 12 solvents (4 per plate).

Four oils (200 nL each) were dispensed using an SPT Labtech mosquito® liquid-handling robot onto a SWISSSCI LCP glass plate with a 100 µm spacer (aspirate 1.25 mm s^{-1}, dispense 1.25 mm s^{-1}). Two columns for no-oil encapsulation were left for comparison (Fig. S1).

After which a total of 50 nL of compound stock solution was injected into each oil droplet (aspirate 25 mm s^{-1}, dispense 25 mm s^{-1}).

Plates were then sealed with a glass cover slip and left for 14 days before inspection for crystallisation.

**Volume of Oil**		**200 nL**											
**Volume of Solvent**		**50 nL**											
**Solvents**		**1**	**2**	**3**	**4**	**5**	**6**	**7**	**8**	**9**	**10**	**11**	**12**
DMSO	**A**	No oil	PDMSO					No oil	FY				
DMSO	**B**	No oil	FC-40					No oil	MO				
DMF	**C**	No oil	PDMSO					No oil	FY				
DMF	**D**	No oil	FC-40					No oil	MO				
MeOH	**E**	No oil	PDMSO					No oil	FY				
MeOH	**F**	No oil	FC-40					No oil	MO				
2,2,2-TFE	**G**	No oil	PDMSO					No oil	FY				
2,2,2-TFE	**H**	No oil	FC-40					No oil	MO				

**Volume of Oil**		**200 nL**											
**Volume of Solvent**		**50 nL**											
**Solvents**		**1**	**2**	**3**	**4**	**5**	**6**	**7**	**8**	**9**	**10**	**11**	**12**
Toluene	**A**	No oil	PDMSO					No oil	FY				
Toluene	**B**	No oil	FC-40					No oil	MO				
DCE	**C**	No oil	PDMSO					No oil	FY				
DCE	**D**	No oil	FC-40					No oil	MO				
2-MeTHF	**E**	No oil	PDMSO					No oil	FY				
2-MeTHF	**F**	No oil	FC-40					No oil	MO				
1,4-Dioxane	**G**	No oil	PDMSO					No oil	FY				
1,4-Dioxane	**H**	No oil	FC-40					No oil	MO				

**Volume of Oil**		**200 nL**											
**Volume of Solvent**		**50 nL**											
**Solvents**		**1**	**2**	**3**	**4**	**5**	**6**	**7**	**8**	**9**	**10**	**11**	**12**
EtOAc	**A**	No oil	PDMSO					No oil	FY				
EtOAc	**B**	No oil	FC-40					No oil	MO				
MeCN	**C**	No oil	PDMSO					No oil	FY				
MeCN	**D**	No oil	FC-40					No oil	MO				
MIBK	**E**	No oil	PDMSO					No oil	FY				
MIBK	**F**	No oil	FC-40					No oil	MO				
$MeNO_2$	**G**	No oil	PDMSO					No oil	FY				
$MeNO_2$	**H**	No oil	FC-40					No oil	MO				

Figure S1. Oil and solvent plate layout for single component crystallisation experiments (top: SP1, middle: SP2 and bottom: SP3).

S3.2.2 Binary High-Throughput Co-Crystallisation

The following method was used to set up binary co-crystallisation experiments, utilising 2 plates (BP1 and BP2) covering 2 solvents per plate (solvent A: MeOH; solvent B: DMF; solvent C: 1,4-dioxane; solvent D: $MeNO_2$) and ratios 2:1, 1:1 and 1:2.

Four oils (200 nL each) were dispensed using an SPT Labtech mosquito® liquid-handling robot onto a SWISSSCI LCP glass plate with a 100 µm spacer (aspirate 1.25 mm s^{-1}, dispense 1.25 mm s^{-1}). Three columns for no-oil encapsulation were left for comparison (Fig. S2).

After which a total of 150 nL of compound and co-former stock solution was injected into each oil droplet (aspirate 25 mm s^{-1}, dispense 25 mm s^{-1}) with varying ratios across the plate: 50 nL: 100 nL, 75 nL: 75 nL, and 100 nL: 50 nL. This was achieved through pick-up of the appropriate volume of compound stock solution, followed by the pick-up of the appropriate volume of co-former stock solution into the same needle, and injection of the combined sample into each oil droplet.

Plates were then sealed with a glass cover slip and left for 14 days before inspection for crystallisation.

**Volume of Oil**		**200 nL**											
**Volume of Stock Solution (A : B)**		**2 : 1 (150 nL)**				**1 : 1 (150 nL)**				**1 : 2 (150 nL)**			
**Solvent**		**1**	**2**	**3**	**4**	**5**	**6**	**7**	**8**	**9**	**10**	**11**	**12**
Solvent A	A	no oil	PDMSO			no oil	PDMSO			no oil	PDMSO		
Solvent A	B	no oil	FC-40			no oil	FC-40			no oil	FC-40		
Solvent A	C	no oil	FY			no oil	FY			no oil	FY		
Solvent A	D	no oil	MO			no oil	MO			no oil	MO		
Solvent B	E	no oil	PDMSO			no oil	PDMSO			no oil	PDMSO		
Solvent B	F	no oil	FC-40			no oil	FC-40			no oil	FC-40		
Solvent B	G	no oil	FY			no oil	FY			no oil	FY		
Solvent B	H	no oil	MO			no oil	MO			no oil	MO		

**Volume of Oil**		**200 nL**											
**Volume of Stock Solution (A : B)**		**2 : 1 (150 nL)**				**1 : 1 (150 nL)**				**1 : 2 (150 nL)**			
**Solvent**		**1**	**2**	**3**	**4**	**5**	**6**	**7**	**8**	**9**	**10**	**11**	**12**
Solvent C	A	no oil	PDMSO			no oil	PDMSO			no oil	PDMSO		
Solvent C	B	no oil	FC-40			no oil	FC-40			no oil	FC-40		
Solvent C	C	no oil	FY			no oil	FY			no oil	FY		
Solvent C	D	no oil	MO			no oil	MO			no oil	MO		
Solvent D	E	no oil	PDMSO			no oil	PDMSO			no oil	PDMSO		
Solvent D	F	no oil	FC-40			no oil	FC-40			no oil	FC-40		
Solvent D	G	no oil	FY			no oil	FY			no oil	FY		
Solvent D	H	no oil	MO			no oil	MO			no oil	MO		

Figure S2. Oil and solvent plate layout for binary co-crystallisation experiments (top: BP1 and bottom: BP2).

S3.2.3 Ternary High-Throughput Co-Crystallisation

The following method was used to set up ternary co-crystallisation experiments, utilising 3 plates (TP1, TP2 and TP3) covering 4 solvents per plate (solvent A: MeOH; solvent B: DMF; solvent C: 1,4-dioxane; solvent D: $MeNO_2$) and ratios 2:1:1, 1:2:1, and 1:1:2 (TP1), 2:2:1, 2:1:2 and 1:2:2 (TP2) and 1:1:1 (TP3) (Fig. S3).

Four oils (200 nL each) were dispensed using an SPT Labtech mosquito® liquid-handling robot onto a SWISSSCI LCP glass plate with a 100 µm spacer (aspirate 1.25 mm s^{-1}, dispense 1.25 mm s^{-1}). TP3 included two no-oil columns for comparison.

After which each compound stock solution was injected into each oil droplet (aspirate 25 mm s^{-1}, dispense 25 mm s^{-1}) with varying ratios across the three plates. TP1: 70 nL: 35 nL: 35 nL, 35 nL: 70 nL: 35 nL and 35 nL: 35 nL: 70 nL (total volume 140 nL), TP2: 55 nL: 55 nL: 30 nL, 55 nL: 30 nL: 55 nL and 30 nL: 55 nL: 55 nL (total volume 140 nL) and TP3: 50 nL: 50 nL: 50 nL (total volume 150 nL). This was achieved through pick-up of the appropriate volume of one compound stock solution, followed by the pick-up of the appropriate volume of stock solution for compounds two and three into the same needle, and injection of the combined samples into each oil droplet.

Plates were then sealed with a glass cover slip and left for 14 days before inspection for crystallisation.

Volume of Oil		200 nL											
Volume of Stock Solution (A : B : C)		2 : 1 : 1 (140 nL)				1 : 2 : 1 (140 nL)				1 : 1 : 2 (140 nL)			
Solvent		1	2	3	4	5	6	7	8	9	10	11	12
Solvent A	A	PDMSO	FY	PDMSO	FY	PDMSO	FY	PDMSO	FY	PDMSO	FY	PDMSO	FY
Solvent A	B	FC-40	MO	FC-40	MO	FC-40	MO	FC-40	MO	FC-40	MO	FC-40	MO
Solvent B	C	PDMSO	FY	PDMSO	FY	PDMSO	FY	PDMSO	FY	PDMSO	FY	PDMSO	FY
Solvent B	D	FC-40	MO	FC-40	MO	FC-40	MO	FC-40	MO	FC-40	MO	FC-40	MO
Solvent C	E	PDMSO	FY	PDMSO	FY	PDMSO	FY	PDMSO	FY	PDMSO	FY	PDMSO	FY
Solvent C	F	FC-40	MO	FC-40	MO	FC-40	MO	FC-40	MO	FC-40	MO	FC-40	MO
Solvent D	G	PDMSO	FY	PDMSO	FY	PDMSO	FY	PDMSO	FY	PDMSO	FY	PDMSO	FY
Solvent D	H	FC-40	MO	FC-40	MO	FC-40	MO	FC-40	MO	FC-40	MO	FC-40	MO

Volume of Oil		200 nL											
Volume of Stock Solution (A : B : C)		2 : 2 : 1 (140 nL)				1 : 2 : 2 (140 nL)				2 : 1 : 2 (140 nL)			
Solvent		1	2	3	4	5	6	7	8	9	10	11	12
Solvent A	A	PDMSO	FY	PDMSO	FY	PDMSO	FY	PDMSO	FY	PDMSO	FY	PDMSO	FY
Solvent A	B	FC-40	MO	FC-40	MO	FC-40	MO	FC-40	MO	FC-40	MO	FC-40	MO
Solvent B	C	PDMSO	FY	PDMSO	FY	PDMSO	FY	PDMSO	FY	PDMSO	FY	PDMSO	FY
Solvent B	D	FC-40	MO	FC-40	MO	FC-40	MO	FC-40	MO	FC-40	MO	FC-40	MO
Solvent C	E	PDMSO	FY	PDMSO	FY	PDMSO	FY	PDMSO	FY	PDMSO	FY	PDMSO	FY
Solvent C	F	FC-40	MO	FC-40	MO	FC-40	MO	FC-40	MO	FC-40	MO	FC-40	MO
Solvent D	G	PDMSO	FY	PDMSO	FY	PDMSO	FY	PDMSO	FY	PDMSO	FY	PDMSO	FY
Solvent D	H	FC-40	MO	FC-40	MO	FC-40	MO	FC-40	MO	FC-40	MO	FC-40	MO

Volume of Oil		200 nL											
Volume of Stock Solution (A : B : C)		1 : 1 : 1 (150 nL)											
**Solvent**		**1**	**2**	**3**	**4**	**5**	**6**	**7**	**8**	**9**	**10**	**11**	**12**
Solvent A	**A**	No oil	PDMSO					No oil	FY				
Solvent A	**B**	No oil	FC-40					No oil	MO				
Solvent B	**C**	No oil	PDMSO					No oil	FY				
Solvent B	**D**	No oil	FC-40					No oil	MO				
Solvent C	**E**	No oil	PDMSO					No oil	FY				
Solvent C	**F**	No oil	FC-40					No oil	MO				
Solvent D	**G**	No oil	PDMSO					No oil	FY				
Solvent D	**H**	No oil	FC-40					No oil	MO				

Figure S3. Oil and solvent plate layouts for ternary co-crystallisation experiments. Top: TP1 covering ratios 2:1:1, 1:2:1 and 1:1:2. Middle: TP2 covering ratios 2:2:1, 2:1:2 and 1:2:2. Bottom: TP3 covering ratio 1:1:1 and the no-oil combination in columns 1 and 7.

S3.2.4 Quaternary High-Throughput Co-Crystallisation

The following method was used to set up quaternary co-crystallisation experiments, utilising 1 plate (QP1) covering 4 solvents per plate (solvent A: MeOH; solvent B: DMF; solvent C: 1,4-dioxane; solvent D: $MeNO_2$) and ratio 1:1:1:1 (Fig. S4).

Four oils (200 nL each) were dispensed using an SPT Labtech mosquito® liquid-handling robot onto a SWISSSCI LCP glass plate with a 100 µm spacer (aspirate 1.25 mm s^{-1}, dispense 1.25 mm s^{-1}). Two no-oil columns were included for comparison.

After which a total of 140 nL of each compound stock solution was injected into each oil droplet (aspirate 25 mm s^{-1}, dispense 25 mm s^{-1}) in a 1:1:1:1 ratio across the plate: 35 nL: 35 nL: 35 nL: 35 nL (total volume 140 nL). This was achieved through pick-up of the appropriate volume of one compound stock solution, followed by the pick-up of the appropriate volume of stock solution for compounds two, three and four into the same needle, and injection of the combined sample into each oil droplet. Note that the order of the pick-up of these compounds was selected based on the idea of potential interaction in the final crystal.

Plates were then sealed with a glass cover slip and left for 14 days before inspection for crystallisation.

**Volume of Oil**		**200 nL**											
**Volume of Stock Solution (A : B : C :D)**		**1 : 1 : 1 : 1 (140 nL)**											
**Solvent**		**1**	**2**	**3**	**4**	**5**	**6**	**7**	**8**	**9**	**10**	**11**	**12**
Solvent A	**A**	No oil	PDMSO					No oil	FY				
Solvent A	**B**	No oil	FC-40					No oil	MO				
Solvent B	**C**	No oil	PDMSO					No oil	FY				
Solvent B	**D**	No oil	FC-40					No oil	MO				
Solvent C	**E**	No oil	PDMSO					No oil	FY				
Solvent C	**F**	No oil	FC-40					No oil	MO				
Solvent D	**G**	No oil	PDMSO					No oil	FY				
Solvent D	**H**	No oil	FC-40					No oil	MO				

Figure S4. Oil and solvent plate layout for quaternary co-crystallisation experiments. QP1 covering ratio 1:1:1:1, including two no-oil columns 1 and 7.

S4. High-Throughput Co-Crystallisation Results

S4.1 Classification of Crystallisation Outcomes by Optical Microscopy

After 14 days, the 96-well plates containing crystallisation and co-crystallisation experiments were examined by cross-polarised light microscopy, and the results of the ENaCt experiments in each well were classified as: F: fail (caused by a dispensing failure, resulting in no encapsulated droplet formation within the well); 1: remains in solution; 2: oiled-out or non-crystalline solid; 3: micro-crystalline solid; 4: crystals suitable for X-ray diffraction analysis (Figure S5).

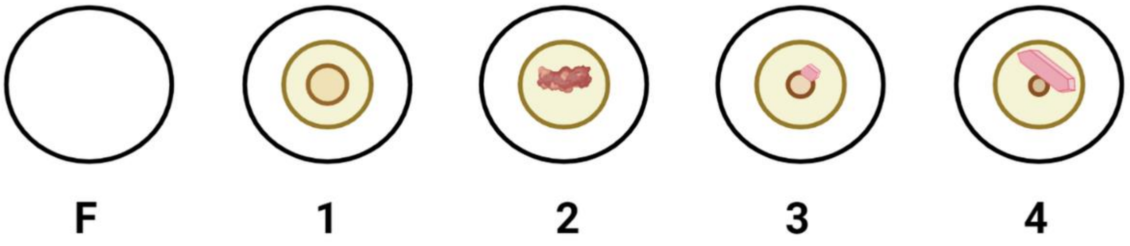

Figure S5. Top view of an ENaCt experiment where F = fail, 1 = sample remaining in solution, 2 = amorphous or oily material, 3 = microcrystalline sample and 4 = single crystal(s) suitable for SCXRD analysis.

S4.2 Selection of Crystals and Co-Crystals for SCXRD Analysis

After the classification of crystallisation outcomes by optical microscopy was completed, crystals which had been classified as "grade 4" were selected for further analysis by SCXRD. In each set of experiments, selected "grade 4" crystals were removed from the 96-well plates and unit cell parameters were obtained by SCXRD analysis to allow crystal form identification.

In order to search for target crystal forms, unit cell analysis was undertaken for all "grade 4" crystals showing different morphology, and at least one example of a "grade 4" crystal from each experimental condition (molecular ratios, oil and solvent).

Full data collections were obtained by SCXRD for all new crystals/co-crystals and for confirmation of known forms.

S4.3 Results of ENaCt Crystallisation for Single Component Systems

S4.3.1 Crystallisation of Substrates

4,4'-bipyridine (**1**), 4,4'-bipyridine dihydrate; CSD refcode: WOVYEL, conditions: MeOH/PDMSO

nicotinamide (**3**); CSD refcode: NICOAM, α polymorph, conditions: MeOH/FC-40

S4.3.2 Crystallisation of Co-formers

2,4-dihydroxybenzoic acid (**4**); CSD refcode: ZZZEEU01, polymorph II, conditions: MeOH/MO

3,5-dinitrobenzoic acid (**5**); CSD refcode: CUKCAM02, C2/c polymorph, conditions: MeOH/MO

glutaric acid (**6**), CSD refcode: GLURAC02, monoclinic III polymorph, conditions: MIBK/FY

3-hydroxy-2-naphthoic acid (**7**); CSD refcode: HNAPAC, Conditions: DMF/MO

methyl gallate (**8**); CSD refcode: ROMGAC, Conditions: DMSO/FY

quinol (**9**); CSD refcode: HYQUIN04, beta polymorph, Conditions: DMF/FY.

S4.4 Co-Crystallisation 96-Well Plate Readouts including Classifications and Crystals Selected for SCXRD Analysis

In the following 96-well plate readouts, crystals analysed by SCXRD with full data collection and structure refinement are indicated by *, ** or ***, and the crystal growth conditions noted. Crystals analysed by SCXRD where only unit cell parameters were measured are indicated with a superscript letter ([a, b, c] etc.).

S4.4.1 Binary Co-Crystallisation 96-Well Plate Readouts

4,4’-bipyridine: 2,4-dihydroxybenzoic acid

4,4'-bipyridine : 2,4-dihydroxybenzoic acid (A : B) plate 1													
Volume of Oil		200 nL											
Volume of Stock Solution (A : B)		2 : 1 (150 nL)				1 : 1 (150 nL)				1 : 2 (150 nL)			
Solvent		1	2	3	4	5	6	7	8	9	10	11	12
MeOH	A	2	3	3	3	2	3	3	3	2	3	3	3
MeOH	B	2	3	3	3	4[a]	3	3	3	2	3	3	3
MeOH	C	3	4[a]	4	4	2	4[a]	4*	4	3	3	4[a]	4
MeOH	D	2	4	3	3	2	3	3	3	2	3	3	3
DMF	E	3	4[a]	3	3	3	3	4	4[a]	3	4[a]	4	3
DMF	F	3	3	3	3	3	4[a]	4	3	3	3	4[a]	3
DMF	G	3	4	4[a]	4	3	3	4	3	3	3	3	3
DMF	H	3	3	3	3	3	4[a]	3	3	3	3	3	4[a]

4,4'-bipyridine : 2,4-dihydroxybenzoic acid (A : B) plate 2													
Volume of Oil		200 nL											
Volume of Stock Solution (A : B)		2 : 1 (150 nL)				1 : 1 (150 nL)				1 : 2 (150 nL)			
Solvent		1	2	3	4	5	6	7	8	9	10	11	12
$MeNO_2$	A	3	3	3	3	3	3	3	3	3	3	3	3
$MeNO_2$	B	2	3	3	3	3	3	3	3	3	3	3	3
$MeNO_2$	C	2	3	3	3	3	3	3	3	3	3	3	3
$MeNO_2$	D	2	3	3	2	2	3	3	3	3	3	3	3
1,4-Dioxane	E	3	3	3	3	3	3	3	3	3	3	3	3
1,4-Dioxane	F	3	3	3	4[a]	3	4[a]	4	4	3	4[a]	4	4
1,4-Dioxane	G	3	4	4	4	3	4[a]	4	4	3	4	4[a]	4
1,4-Dioxane	H	3	4	3	3	3	4[a]	4	3	3	3	3	3

SCXRD Full Data Collection and Structure Refinement

* Binary co-crystal (4,4’-bipyridine: 2,4-dihydroxybenzoic acid, 1:1; CSD refcode: IDUBUF) obtained from P1 C7 (MeOH, FY).

SCXRD Unit Cell Analysis

[a] Binary co-crystal (4,4’-bipyridine: 2,4-dihydroxybenzoic acid, 1:1; CSD refcode: IDUBUF).

4,4’-bipyridine: 3,5-dinitrobenzoic acid

4,4'-bipyridine : 3,5-dinitrobenzoic acid (A : B) plate 1													
Volume of Oil		200 nL											
Volume of Stock Solution (A : B)		2 : 1 (150 nL)				1 : 1 (150 nL)				1 : 2 (150 nL)			
Solvent		1	2	3	4	5	6	7	8	9	10	11	12
MeOH	A	4	4*	4	4	4[a]	4	4[a]	4	3	4	4	4[a]
MeOH	B	2	3	3	3	2	4[a]	4	4	3	3	3	2
MeOH	C	3	3	4[a]	4	4	4	4[a]	4	2	4[a]	3	4
MeOH	D	4	4[a]	4	4	4	4	4	4[a]	2	4	4[a]	4
DMF	E	2	2	2	2	2	3	2	2	3	3	3	3
DMF	F	2	2	2	2	2	2	2	2	2	2	2	2
DMF	G	3	2	3	2	2	2	2	2	2	2	2	2
DMF	H	2	2	2	2	2	2	2	2	2	2	2	2

4,4'-bipyridine : 3,5-dinitrobenzoic acid (A : B) plate 2													
Volume of Oil		200 nL											
Volume of Stock Solution (A : B)		2 : 1 (150 nL)				1 : 1 (150 nL)				1 : 2 (150 nL)			
Solvent		1	2	3	4	5	6	7	8	9	10	11	12
$MeNO_2$	A	4	3	3	4[b]	4	4	4[b]	4	4	4	4	4[b]
$MeNO_2$	B	4	4[b]	4	4	4[b]	4	4**	4	3	4	4[b]	4
$MeNO_2$	C	4	4	4[b]	4	4	4	4	4	3	4[b]	4	4
$MeNO_2$	D	4	4[b]	4	4	4	4	4	4[b]	4	4	4	4[b]
1,4-Dioxane	E	2	2	2	2	2	2	2	2	2	2	2	2
1,4-Dioxane	F	3	3	3	3	3	3	3	3	3	3	4[a]	4
1,4-Dioxane	G	2	2	2	3	3	2	4[a]	3	3	4	4[a]	3
1,4-Dioxane	H	2	3	3	3	3	3	3	3	3	3	3	3

SCXRD Full Data Collection and Structure Refinement

* Binary co-crystal (4,4’-bipyridine: 3,5-dinitrobenzoic acid, 0.5:1; CSD refcode: FIHYEA) obtained from P1 A2 (MeOH, PDMSO oil).

** Binary co-crystal (4,4’-bipyridine: 3,5-dinitrobenzoic acid, 1:1) obtained from P2 B7 ($MeNO_2$, FC-40 oil).

SCXRD Unit Cell Analysis

[a] Binary co-crystal (4,4’-bipyridine: 3,5-dinitrobenzoic acid, 0.5:1; CSD refcode: FIHYEA).

[b] Binary co-crystal (4,4’-bipyridine: 3,5-dinitrobenzoic acid, 1:1).

4,4’-bipyridine: glutaric acid

4,4'-bipyridine : glutaric acid (A : B) plate 1													
Volume of Oil		200 nL											
Volume of Stock Solution (A : B)		2 : 1 (150 nL)				1 : 1 (150 nL)				1 : 2 (150 nL)			
Solvent		1	2	3	4	5	6	7	8	9	10	11	12
MeOH	A	2	3	3	3	4[a]	3	3	3	4	4[a]	3	3
MeOH	B	3	4[a]	4	4	3	4[a]	4	4[a]	4	4[a]	4	3
MeOH	C	4	4	4[a]	4	4	4[a]	4	4	3	4[a]	4	4
MeOH	D	2	4[a]	3	4	2	4	4	4[a]	4	3	3	4*
DMF	E	3	3	3	3	3	3	4[a]	3	3	3	3	3
DMF	F	3	3	3	3	3	3	3	3	3	4[a]	3	3
DMF	G	3	3	3	3	3	3	4	4[a]	3	3	3	4
DMF	H	3	3	3	3	3	3	3	3	3	3	4[a]	4

4,4'-bipyridine : glutairc acid (A : B) plate 2													
Volume of Oil		200 nL											
Volume of Stock Solution (A : B)		2 : 1 (150 nL)				1 : 1 (150 nL)				1 : 2 (150 nL)			
Solvent		1	2	3	4	5	6	7	8	9	10	11	12
$MeNO_2$	A	3	3	3	3	3	3	3	3	3	3	3	3
$MeNO_2$	B	4	4[a]	4	4	4[a]	4	4	4[a]	3	4	4[a]	4
$MeNO_2$	C	4	4[a]	4[a]	4	4	4	4[a]	4	4[a]	4	4	4[a]
$MeNO_2$	D	4[a]	4	4[a]	4	3	4	4[a]	4	3	4[a]	4	4
1,4-Dioxane	E	4[a]	3	3	3	3	3	3	3	4[a]	3	3	3
1,4-Dioxane	F	3	3	3	3	3	3	3	3	3	3	3	3
1,4-Dioxane	G	3	4	4[a]	4	3	4	3	3	3	4[a]	4	4
1,4-Dioxane	H	3	3	3	3	3	3	3	3	3	3	3	3

SCXRD Full Data Collection and Structure Refinement

* Binary co-crystal (4,4’-bipyridine: glutaric acid, 2:2; CSD refcode: SOVDIQ) obtained from P1 D12 (MeOH, MO oil).

SCXRD Unit Cell Analysis

[a] Binary co-crystal (4,4’-bipyridine: glutaric acid, 2:2; CSD refcode: SOVDIQ).

4,4’-bipyridine: 3-hydroxy-2-naphthoic acid

4,4'-bipyridine : 3-hydroxy-2-napthoic acid (A : B) plate 1													
**Volume of Oil**		**200 nL**											
**Volume of Stock Solution (A : B)**		**2 : 1 (150 nL)**				**1 : 1 (150 nL)**				**1 : 2 (150 nL)**			
**Solvent**		**1**	**2**	**3**	**4**	**5**	**6**	**7**	**8**	**9**	**10**	**11**	**12**
MeOH	**A**	2	3	2	2	2	3	3	3	2	3	3	3
MeOH	**B**	3	3	4[a]	3	3	3	3	3	2	2	3	3
MeOH	**C**	4[a]	4	4	4[a]	3	4	4*	3	3	4[a]	4	3
MeOH	**D**	3	4	4	4[a]	3	4	4[a]	4	3	4[a]	4	4
DMF	**E**	2	4[a]	4	4	4[a]	4	3	3	3	4[a]	3	4
DMF	**F**	3	4	4	4	4[a]	4	3	4[a]	2	3	3	3
DMF	**G**	2	4	4	4[a]	3	4	3	4	3	4[a]	4[a]	4
DMF	**H**	4[a]	4	4	4[a]	4	4	4	4[a]	4	4	4	4[a]

4,4'-bipyridine : 3-hydroxy-2-napthoic (A : B) plate 2													
**Volume of Oil**		**200 nL**											
**Volume of Stock Solution (A : B)**		**2 : 1 (150 nL)**				**1 : 1 (150 nL)**				**1 : 2 (150 nL)**			
**Solvent**		**1**	**2**	**3**	**4**	**5**	**6**	**7**	**8**	**9**	**10**	**11**	**12**
$MeNO_2$	**A**	3	3	3	3	2	3	3	4**	2	4[b]	4	4
$MeNO_2$	**B**	3	3	3	3	2	3	3	3	2	4	4[b]	4
$MeNO_2$	**C**	3	3	4[b]	4[a]	3	3	3	4[a]	3	3	3	3
$MeNO_2$	**D**	2	3	3	3	2	3	4	4[b]	3	4	3	4[b]
1,4-Dioxane	**E**	3	2	2	3	3	2	3	3	3	3	3	3
1,4-Dioxane	**F**	3	4[a]	4	4	4[a]	4	3	4[a]	3	4[a]	3	3
1,4-Dioxane	**G**	3	4	4[a]	4	3	4	4	4[a]	3	4	4[a]	4
1,4-Dioxane	**H**	3	3	3	3	3	3	4[a]	3	3	3	3	3

SCXRD Full Data Collection and Structure Refinement

* Binary co-crystal (4,4’-bipyridine: 3-hydroxy-2-naphthoic acid, 0.5:1; CSD refcode: GEHROB) obtained from P1 C7 (MeOH, FY).

** Binary co-crystal (4,4’-bipyridine: 3-hydroxy-2-naphthoic acid, 1.5:1) obtained from P2 A8 ($MeNO_2$, PDMSO).

SCXRD Unit Cell Analysis

[a] Binary co-crystal (4,4’-bipyridine: 3-hydroxy-2-naphthoic acid, 0.5:1; CSD refcode: GEHROB).

[b] Binary co-crystal (4,4’-bipyridine: 3-hydroxy-2-naphthoic acid, 1.5:1).

4,4’-bipyridine: methyl gallate

4,4'-bipyridine : methyl gallate (A : B) plate 1													
Volume of Oil		200 nL											
Volume of Stock Solution (A : B)		2 : 1 (150 nL)				1 : 1 (150 nL)				1 : 2 (150 nL)			
Solvent		1	2	3	4	5	6	7	8	9	10	11	12
MeOH	A	2	3	3	3	4	2	2	2	3	4	4[a]	4
MeOH	B	2	3	4*	4	4[a]	3	2	3	2	2	3	3
MeOH	C	2	4[a]	4	3	1	3	1	3	2	4[a]	3	4
MeOH	D	2	4	4[a]	4	4	3	4[a]	3	2	4[a]	3	4
DMF	E	2	4	4	4	4	4	4	2	4	4	2	2
DMF	F	3	4	4	4**	4[b]	4	4[b]	4	4	4[b]	4	3
DMF	G	3	3	4[b]	4	3	4	4	4	4[b]	2	2	4
DMF	H	3	3	4	4[b]	4	4[b]	4	4	4	3	4[b]	4

4,4'-bipyridine : methyl gallate (A : B) plate 2													
Volume of Oil		200 nL											
Volume of Stock Solution (A : B)		2 : 1 (150 nL)				1 : 1 (150 nL)				1 : 2 (150 nL)			
Solvent		1	2	3	4	5	6	7	8	9	10	11	12
$MeNO_2$	A	2	4	4[c]	4	4[c]	4[c]	2	3	2	2	2	3
$MeNO_2$	B	2	4***	4	3	4[c]	2	2	3	3	3	2	2
$MeNO_2$	C	2	3	3	3	3	3	2	3	2	2	3	3
$MeNO_2$	D	2	4[c]	4[c]	4****	4[c]	4[c]	2	3	3	4[c]	3	3
1,4-Dioxane	E	2	2	2	2	2	2	2	2	2	4[a]	2	2
1,4-Dioxane	F	2	3	3	3	3	2	2	3	3	4[a]	3	3
1,4-Dioxane	G	2	3	3	3	3	3	2	2	2	2	2	3
1,4-Dioxane	H	2	3	2	2	3	3	2	2	2	2	2	2

SCXRD Full Data Collection and Structure Refinement

* Binary co-crystal (4,4’-bipyridine: methyl gallate: H_2O, 2:2:3) obtained from P1 B3 (MeOH, FC-40 oil).

** Binary co-crystal (4,4’-bipyridine: methyl gallate: DMF: H_2O, 3:2:1:2) obtained from P1 F4 (DMF, FC-40 oil).

*** Full data collection obtained, structure solution inconclusive.

**** Binary co-crystal (4,4’-bipyridine: methyl gallate: $MeNO_2$: H_2O, 3:2:2:2) obtained from P2 D4 ($MeNO_2$, MO oil).

SCXRD Unit Cell Analysis

[a] Binary co-crystal (4,4’-bipyridine: methyl gallate: H_2O, 2:2:3).

[b] Binary co-crystal (4,4’-bipyridine: methyl gallate: DMF: H_2O, 3:2:1:2).

[c] Binary co-crystal (4,4’-bipyridine: methyl gallate: $MeNO_2$: H_2O, 3:2:2:2).

4,4’-bipyridine: quinol

4,4'-bipyridine : quinol (A : B) plate 1													
Volume of Oil		200 nL											
Volume of Stock Solution (A : B)		2 : 1 (150 nL)				1 : 1 (150 nL)				1 : 2 (150 nL)			
Solvent		1	2	3	4	5	6	7	8	9	10	11	12
MeOH	A	4	4[a]	4	4	4[a]	4	4[a]	2	4	4	2	4[a]
MeOH	B	4	4	4[a]	4	4	4	4	4[a]	4	4[a]	4	4
MeOH	C	4[a]	2	4	4	4[a]	2	2	3	4	4[a]	2	4
MeOH	D	4	4[a]	4	4	4	4	4	4[a]	4[a]	3	3	4
DMF	E	2	4	4[a]	4	2	4[a]	4	2	2	4[a]	4[a]	4
DMF	F	2	4[a]	4[a]	4	2	3	2	2	2	3	2	3
DMF	G	2	3	3	3	2	2	2	2	2	2	2	2
DMF	H	2	2	2	2	2	2	2	2	2	2	2	2

4,4'-bipyridine : quinol (A : B) plate 2													
Volume of Oil		200 nL											
Volume of Stock Solution (A : B)		2 : 1 (150 nL)				1 : 1 (150 nL)				1 : 2 (150 nL)			
Solvent		1	2	3	4	5	6	7	8	9	10	11	12
$MeNO_2$	A	3	3	3	3	3	3	3	3	3	3	3	3
$MeNO_2$	B	3	3	4[a]	3	3	3	3	3	3	3	4[a]	4
$MeNO_2$	C	3	3	4[a]	3	3	4	4[a]	4	3	3	3	3
$MeNO_2$	D	3	3	3	4[a]	4[a]	4[a]	3	3	3	3	3	3
1,4-Dioxane	E	2	3	2	3	4[a]	3	2	2	3	2	2	3
1,4-Dioxane	F	2	4[a]	4	4[a]	3	4	4*	4	4[a]	4	4[a]	4[a]
1,4-Dioxane	G	2	4	4[a]	4	2	4[a]	3	3	2	3	3	2
1,4-Dioxane	H	2	2	2	3	2	2	2	3	2	4	4[a]	4

SCXRD Full Data Collection and Structure Refinement

* Binary co-crystal (4,4’-bipyridine: quinol, 1:0.5; CSD refcode: QAMRUS) obtained from P2 F7 (1,4-Dioxane, FC-40 oil).

SCXRD Unit Cell Analysis

[a] Binary co-crystal (4,4’-bipyridine: quinol, 1:0.5; CSD refcode: QAMRUS).

caffeine: 2,4-dihydroxybenzoic acid

caffeine : 2,4-dihydroxybenzoic acid (A : B) plate 1													
Volume of Oil		200 nL											
Volume of Stock Solution (A : B)		2 : 1 (150 nL)				1 : 1 (150 nL)				1 : 2 (150 nL)			
Solvent		1	2	3	4	5	6	7	8	9	10	11	12
MeOH	A	2	4[a]	4	4	2	2	2	2	2	4[a]	4[a]	4
MeOH	B	2	4[a]	4	2	2	3	2	2	2	4	3	3
MeOH	C	2	4[a]	4	4	2	3	4[a]	4	2	3	3	3
MeOH	D	2	4[a]	4	2	2	2	3	4[a]	2	3	4	4[a]
DMF	E	3	3	3	3	3	3	3	3	3	3	3	3
DMF	F	3	3	3	3	3	3	3	3	3	3	3	3
DMF	G	3	1	1	3	3	3	1	3	3	3	3	3
DMF	H	3	1	3	3	3	3	3	3	3	3	3	3

caffeine : 2,4-dihydroxybenzoic acid (A : B) plate 2													
Volume of Oil		200 nL											
Volume of Stock Solution (A : B)		2 : 1 (150 nL)				1 : 1 (150 nL)				1 : 2 (150 nL)			
Solvent		1	2	3	4	5	6	7	8	9	10	11	12
$MeNO_2$	A	3	4[a]	4	4	3	4[a]	3	4	3	3	3	3
$MeNO_2$	B	3	4	4[a]	3	3	3	3	3	3	3	3	4[a]
$MeNO_2$	C	3	4[a]	4	4	3	4	4[a]	4	3	3	3	4[a]
$MeNO_2$	D	3	3	3	4[a]	3	4[a]	4	4	3	4[a]	4	4
1,4-Dioxane	E	3	3	3	3	2	3	3	3	2	3	4[a]	3
1,4-Dioxane	F	2	3	3	3	2	3	3	3	2	3	3	3
1,4-Dioxane	G	4[a]	4[a]	4	3	2	3	4[a]	3	2	4*	4	3
1,4-Dioxane	H	3	3	3	3	2	3	3	3	2	3	3	3

SCXRD Full Data Collection and Structure Refinement

* Binary co-crystal hydrate (caffeine: 2,4-dihydroxybenzoic acid: H_2O, 1:1:1; CSD refcode: MOZCIO) obtained from P2 G10 (1,4-dioxane, FY oil).

SCXRD Unit Cell Analysis

[a] Binary co-crystal hydrate (caffeine: 2,4-dihydroxybenzoic acid: H_2O, 1:1:1; CSD refcode: MOZCIO).

caffeine: 3,5-dinitrobenzoic acid

caffeine : 3,5-dinitrobenzoic acid (A : B) plate 1													
Volume of Oil		200 nL											
Volume of Stock Solution (A : B)		2 : 1 (150 nL)				1 : 1 (150 nL)				1 : 2 (150 nL)			
Solvent		1	2	3	4	5	6	7	8	9	10	11	12
MeOH	A	2	3	4[a]	2	4	4[a]	4	2	3	3	3	3
MeOH	B	3	4	3	3	3	4[a]	4[a]	4	3	3	3	3
MeOH	C	3	4[a]	3	3	3	3	3	3	3	3	3	3
MeOH	D	3	3	3	3	3	4[a]	4	4	3	4	4**	4
DMF	E	3	2	2	2	2	2	2	2	2	2	2	2
DMF	F	3	2	2	2	2	2	2	2	2	2	2	2
DMF	G	3	4[b]	4[b]	3	4[b]	3	4*	4[b]	2	2	2	2
DMF	H	2	3	2	3	2	3	3	3	2	2	2	2

caffeine : 3,5-dinitrobenzoic acid (A : B) plate 2													
Volume of Oil		200 nL											
Volume of Stock Solution (A : B)		2 : 1 (150 nL)				1 : 1 (150 nL)				1 : 2 (150 nL)			
Solvent		1	2	3	4	5	6	7	8	9	10	11	12
$MeNO_2$	A	2	2	2	3	2	3	3	3	2	2	2	2
$MeNO_2$	B	2	2	3	3	2	2	3	3	2	3	2	3
$MeNO_2$	C	2	2	3	3	2	3	3	2	2	3	3	3
$MeNO_2$	D	2	3	3	3	2	3	3	3	2	3	2	3
1,4-Dioxane	E	2	2	2	2	2	2	2	2	2	2	2	2
1,4-Dioxane	F	2	4[c]	4	2	3	4***	4	3	2	4	3	4[c]
1,4-Dioxane	G	2	2	2	3	3	3	3	3	3	3	3	3
1,4-Dioxane	H	2	3	4[c]	4	2	3	4[c]	4	3	4	4[c]	4

SCXRD Full Data Collection and Structure Refinement

* Binary co-crystal (caffeine: 3,5-dinitrobenzoic acid, 2:2) obtained from P1 G7 (DMF, FY oil).

** Single component crystal (3,5-dinitrobenzoic acid; CSD refcode: CUKCAM14) obtained from P1 D11 (MeOH, MO oil).

*** Solvate (3,5-dinitrobenzoic acid: 1,4-dioxane) obtained from P2 F6 (1,4-dioxane, FC-40 oil).

SCXRD Unit Cell Analysis

[a] Single-component crystal (3,5-dinitrobenzoic acid; CSD refcode: CUKCAM14).

[b] Binary co-crystal (caffeine: 3,5-dinitrobenzoic acid, 2:2).

[c] Solvate (3,5-dinitrobenzoic acid: 1,4-dioxane).

caffeine: glutaric acid

caffeine : glutaric acid (A : B) plate 1													
**Volume of Oil**		**200 nL**											
**Volume of Stock Solution (A : B)**		**2 : 1 (150 nL)**				**1 : 1 (150 nL)**				**1 : 2 (150 nL)**			
**Solvent**		**1**	**2**	**3**	**4**	**5**	**6**	**7**	**8**	**9**	**10**	**11**	**12**
MeOH	**A**	3	4[a]	4	4	3	4	4	4[a]	3	3	4[a]	4
MeOH	**B**	3	3	3	3	3	3	3	3	3	3	3	3
MeOH	**C**	3	3	3	3	3	3	3	3	3	3	3	3
MeOH	**D**	3	3	3	3	3	3	4[a]	4	3	4[a]	4	3
DMF	**E**	1	3	3	1	3	3	3	3	3	3	3	3
DMF	**F**	1	3	3	3	3	3	3	3	3	3	3	3
DMF	**G**	2	3	3	3	3	3	3	3	3	3	3	3
DMF	**H**	1	1	1	3	3	3	3	3	3	3	3	3

caffeine : glutaric acid (A : B) plate 2													
**Volume of Oil**		**200 nL**											
**Volume of Stock Solution (A : B)**		**2 : 1 (150 nL)**				**1 : 1 (150 nL)**				**1 : 2 (150 nL)**			
**Solvent**		**1**	**2**	**3**	**4**	**5**	**6**	**7**	**8**	**9**	**10**	**11**	**12**
$MeNO_2$	**A**	3	3	3	3	3	4[b]	3	3	4*	3	3	4[a,b]
$MeNO_2$	**B**	2	3	3	4[b]	2	3	3	3	3	3	3	3
$MeNO_2$	**C**	2	3	3	4[a]	3	3	3	3	4	4	3	4
$MeNO_2$	**D**	3	3	4[a]	4	3	3	3	3	4	3	3	3
1,4-Dioxane	**E**	2	3	3	3	3	4[a]	4[a]	4[a,b]	3	4	3	3
1,4-Dioxane	**F**	3	3	3	3	3	4[a]	4[a]	3	3	3	3	3
1,4-Dioxane	**G**	3	3	3	1	3	1	1	3	3	3	3	1
1,4-Dioxane	**H**	3	3	3	3	3	3	3	3	3	3	3	3

SCXRD Full Data Collection and Structure Refinement

* Binary co-crystal (caffeine: glutaric acid, 1:2; CSD refcode: EXUQUJ01) obtained from P2 A9 ($MeNO_2$).

SCXRD Unit Cell Analysis

[a] Single-component crystal (glutaric acid; CSD refcode: GLURAC02).

[b] Binary co-crystal (caffeine: glutaric acid, 1:2; CSD refcode: EXUQUJ01).

caffeine: 3-hydroxy-2-naphthoic acid

caffeine : 3-hydroxy-2-napthoic acid (A : B) plate 1													
Volume of Oil		200 nL											
Volume of Stock Solution (A : B)		2 : 1 (150 nL)				1 : 1 (150 nL)				1 : 2 (150 nL)			
Solvent		1	2	3	4	5	6	7	8	9	10	11	12
MeOH	A	3	4[a]	3	3	3	3	3	4[a]	4[a]	3	3	3
MeOH	B	3	3	3	3	3	3	3	3	3	3	3	3
MeOH	C	3	4[a]	3	3	4[a]	4	4[a]	3	3	3	3	3
MeOH	D	3	4[a]	3	3	3	3	3	3	3	3	3	3
DMF	E	3	3	3	3	3	3	4[a]	3	3	3	3	3
DMF	F	3	3	3	4[a]	3	3	3	3	3	3	3	3
DMF	G	3	3	3	3	4[a]	3	3	3	4[a]	3	3	3
DMF	H	4[a]	3	3	3	3	3	3	4[b]	3	3	3	4

caffeine : 3-hydroxy-2-napthoic acid (A : B) plate 2													
Volume of Oil		200 nL											
Volume of Stock Solution (A : B)		2 : 1 (150 nL)				1 : 1 (150 nL)				1 : 2 (150 nL)			
Solvent		1	2	3	4	5	6	7	8	9	10	11	12
$MeNO_2$	A	3	3	3	3	3	3	3	3	3	3	3	3
$MeNO_2$	B	3	3	3	3	3	3	3	3	3	3	3	3
$MeNO_2$	C	3	3	3	3	3	3	3	3	3	3	3	3
$MeNO_2$	D	3	3	3	3	3	3	3	3	3	3	3	3
1,4-Dioxane	E	2	3	3	3	3	3	3	3	3	3	3	4[b]
1,4-Dioxane	F	2	3	3	3	2	3	3	3	3	3	3	3
1,4-Dioxane	G	2	4*	4[a]	3	3	3	3	4[a]	3	4[a]	4[a]	4
1,4-Dioxane	H	2	4[b]	3	3	3	4[a,b]	4[a,b]	3	3	4[a,b]	3	4[a]

SCXRD Full Data Collection and Structure Refinement

* Binary co-crystal (caffeine: 3-hydroxy-2-naphthoic acid, 1:1; CSD refcode: KIGKOB) obtained from P2 G2 (1,4-dixoane, FY oil).

SCXRD Unit Cell Analysis

[a] Binary co-crystal (caffeine: 3-hydroxy-2-naphthoic acid, 1:1; CSD refcode: KIGKOB).

[b] Single-component crystal (glutaric acid; CSD refcode: GLURAC02).

caffeine: methyl gallate

caffeine : methyl gallate (A : B) plate 1													
Volume of Oil		200 nL											
Volume of Stock Solution (A : B)		2 : 1 (150 nL)				1 : 1 (150 nL)				1 : 2 (150 nL)			
Solvent		1	2	3	4	5	6	7	8	9	10	11	12
MeOH	A	3	4[a]	3	3	2	3	4	4[a]	2	3	3	3
MeOH	B	2	3	3	3	2	3	3	4	2	4[a]	4[a]	4
MeOH	C	2	4	4	4[a]	2	4	4[a]	4	2	4	4[a]	4*
MeOH	D	3	3	3	3	2	3	3	3	3	4[a]	4	4[a]
DMF	E	1	3	1	1	1	1	1	3	1	3	3	3
DMF	F	3	3	1	1	1	1	4	3	3	4	4	4[a]
DMF	G	3	1	1	1	1	1	1	1	3	3	1	4
DMF	H	1	1	1	1	1	1	1	1	3	4	4	4

caffeine : methyl gallate (A : B) plate 2													
Volume of Oil		200 nL											
Volume of Stock Solution (A : B)		2 : 1 (150 nL)				1 : 1 (150 nL)				1 : 2 (150 nL)			
Solvent		1	2	3	4	5	6	7	8	9	10	11	12
$MeNO_2$	A	3	3	3	3	3	3	3	3	3	2	3	2
$MeNO_2$	B	3	3	3	3	3	3	4[a]	4	3	4[a]	3	3
$MeNO_2$	C	3	4[a]	3	3	3	4	4[a]	3	3	4[a]	4	4
$MeNO_2$	D	3	4	4[a]	4	3	4	4[a]	4	3	4	4[a]	4
1,4-Dioxane	E	3	3	3	3	2	2	2	2	2	2	2	3
1,4-Dioxane	F	2	4	4	4[a]	2	4[a]	4	4	3	3	3	4
1,4-Dioxane	G	2	4	4	4[a]	2	4[a]	4	4	2	4	4[a]	4
1,4-Dioxane	H	2	3	3	3	2	4[a]	2	4	2	2	3	4[a]

SCXRD Full Data Collection and Structure Refinement

* Binary co-crystal (caffeine: methyl gallate, 1:1; CSD refcode: DIJVOH) obtained from P1 C12 (MeOH, FY oil).

SCXRD Unit Cell Analysis

[a] Binary co-crystal (caffeine: methyl gallate, 1:1; CSD refcode: DIJVOH).

caffeine: quinol

caffeine : quinol (A : B) plate 1													
Volume of Oil		200 nL											
Volume of Stock Solution (A : B)		2 : 1 (150 nL)				1 : 1 (150 nL)				1 : 2 (150 nL)			
Solvent		1	2	3	4	5	6	7	8	9	10	11	12
MeOH	A	2	4	4[a]	4	2	4[a]	4	4[a]	3	4	4[a]	4
MeOH	B	2	3	4[a]	4	2	4	4[a]	4	2	3	3	2
MeOH	C	3	3	4	4[a]	3	4[a]	4	4	2	4	3	4[a]
MeOH	D	2	3	4[a]	4	3	3	4*	4	2	4[a]	4[a]	4
DMF	E	1	3	4	4[b]	1	1	1	1	4[b]	1	1	1
DMF	F	1	1	1	3	1	1	1	1	1	1	2	1
DMF	G	1	1	3	2	1	1	1	1	1	1	1	1
DMF	H	4[b]	4	4[b]	4	1	1	1	1	2	1	1	1

caffeine : quinol (A : B) plate 2													
Volume of Oil		200 nL											
Volume of Stock Solution (A : B)		2 : 1 (150 nL)				1 : 1 (150 nL)				1 : 2 (150 nL)			
Solvent		1	2	3	4	5	6	7	8	9	10	11	12
$MeNO_2$	A	2	3	3	3	3	3	3	3	2	3	3	3
$MeNO_2$	B	2	3	3	3	3	2	2	3	2	2	3	3
$MeNO_2$	C	3	3	4[b]	4	2	3	4	4[b]	2	2	2	3
$MeNO_2$	D	4[b]	4[b]	4	4	2	3	4[b]	3	2	3	4[b]	3
1,4-Dioxane	E	2	2	3	3	2	3	3	4[b]	2	4[b]	4	4
1,4-Dioxane	F	3	4	4[b]	4	4[b]	4[b]	4	4	2	4	4**	4
1,4-Dioxane	G	3	4[b]	4	4	2	4	4[b]	4	2	4[b]	3	4
1,4-Dioxane	H	3	4	4[b]	4	2	4[b]	4	3	2	4	4[b]	4

SCXRD Full Data Collection and Structure Refinement

* Single component crystal (quinol; CSD refcode: HYQUIN04) obtained from P1 D7 (MeOH, MO oil).

** Binary co-crystal (caffeine: quinol, 1:1.5) obtained from P2 F11 (1,4-Dioxane, FC-40 oil).

SCXRD Unit Cell Analysis

[a] Single component crystal (quinol; CSD refcode: HYQUIN04).

[b] Binary co-crystal (caffeine: quinol, 1:1.5).

nicotinamide: 2,4-dihydroxybenzoic acid

nicotinamide : 2,4-dihydroxybenzoic acid (A : B) plate 1													
Volume of Oil		200 nL											
Volume of Stock Solution (A : B)		2 : 1 (150 nL)				1 : 1 (150 nL)				1 : 2 (150 nL)			
Solvent		1	2	3	4	5	6	7	8	9	10	11	12
MeOH	A	2	3	3	3	2	3	2	2	2	3	3	3
MeOH	B	3	4[b]	3	3	2	4[a]	3	3	2	3	3	3
MeOH	C	3	3	3	3	2	4	4	4*	3	3	3	3
MeOH	D	2	3	2	3	2	3	3	3	2	3	4[a]	3
DMF	E	1	3	1	3	1	1	3	3	1	3	1	1
DMF	F	3	1	1	1	1	1	3	1	3	1	1	1
DMF	G	1	1	1	1	1	1	1	1	1	1	1	3
DMF	H	3	1	1	1	1	1	1	3	3	1	1	3

nicotinamide : 2,4-dihydroxybenzoic acid (A : B) plate 2													
Volume of Oil		200 nL											
Volume of Stock Solution (A : B)		2 : 1 (150 nL)				1 : 1 (150 nL)				1 : 2 (150 nL)			
Solvent		1	2	3	4	5	6	7	8	9	10	11	12
$MeNO_2$	A	3	3	2	3	3	3	3	3	3	3	3	3
$MeNO_2$	B	3	3	3	3	3	3	4	4[b]	3	3	3	3
$MeNO_2$	C	3	3	4**	4[b]	3	4	3	4[b]	3	4[b]	3	3
$MeNO_2$	D	3	3	3	3	3	4[b]	3	4	2	3	3	3
1,4-Dioxane	E	3	3	3	3	3	3	3	3	3	3	3	3
1,4-Dioxane	F	3	3	3	3	3	3	3	3	3	3	3	3
1,4-Dioxane	G	3	4	4	4[b]	3	3	4[b]	4	3	4	4[b]	3
1,4-Dioxane	H	3	3	3	3	3	4[b]	3	3	3	3	3	3

SCXRD Full Data Collection and Structure Refinement

* Binary co-crystal solvate (nicotinamide: 2,4-dihydroxybenzoic acid: MeOH, 1:1:1; CSD refcode: DINSEA) obtained from P1 C8 (MeOH, FY oil).

** Binary co-crystal (nicotinamide: 2,4-dihydroxybenzoic acid, 1:1; CSD refcode: DINRUP01) obtained from P2 C3 ($MeNO_2$, FY oil).

SCXRD Unit Cell Analysis

[a] Binary co-crystal solvate (nicotinamide: 2,4-dihydroxybenzoic acid: MeOH, 1:1:1; CSD refcode: DINSEA).

[b] Binary co-crystal (nicotinamide: 2,4-dihydroxybenzoic acid, 1:1; CSD refcode: DINRUP01).

nicotinamide: 3,5-dinitrobenzoic acid

nicotinamide : 3,5-dinitrobenzoic acid (A : B) plate 1													
Volume of Oil		200 nL											
Volume of Stock Solution (A : B)		2 : 1 (150 nL)				1 : 1 (150 nL)				1 : 2 (150 nL)			
Solvent		1	2	3	4	5	6	7	8	9	10	11	12
MeOH	A	3	3	4[a]	4	3	4[a]	4	4	3	4	4[a]	4
MeOH	B	3	4[a]	4	4	3	3	3	3	3	3	3	3
MeOH	C	3	3	3	3	3	3	3	3	4[a]	4	4	4*
MeOH	D	3	3	4[a]	3	4	4[a]	4	3	4	3	3	4[a]
DMF	E	2	1	2	1	2	2	1	1	2	1	1	1
DMF	F	2	1	1	1	1	1	1	1	1	1	1	1
DMF	G	2	2	1	1	1	1	1	1	1	1	1	1
DMF	H	2	2	1	1	2	1	1	1	2	2	1	1

nicotinamide : 3,5-dinitrobenzoic acid (A : B) plate 2													
Volume of Oil		200 nL											
Volume of Stock Solution (A : B)		2 : 1 (150 nL)				1 : 1 (150 nL)				1 : 2 (150 nL)			
Solvent		1	2	3	4	5	6	7	8	9	10	11	12
$MeNO_2$	A	2	2	2	2	2	2	2	2	2	2	2	2
$MeNO_2$	B	2	2	2	2	2	2	2	2	2	3	3	3
$MeNO_2$	C	2	2	2	2	2	2	2	2	2	2	2	2
$MeNO_2$	D	2	3	2	2	2	2	2	2	2	3	3	2
1,4-Dioxane	E	2	2	3	4[b]	2	3	4[b]	4	2	3	3	3
1,4-Dioxane	F	2	2	2	2	2	2	2	2	2	2	4[b]	4
1,4-Dioxane	G	2	4[b]	3	2	2	4[b]	3	3	2	3	3	3
1,4-Dioxane	H	2	3	3	4[b]	2	3	3	3	2	2	4[b]	4

SCXRD Full Data Collection and Structure Refinement

* Binary co-crystal (nicotinamide: 3,5-dinitrobenzoic acid: MeOH, 2:2:2) obtained from P1 C12 (MeOH, FY oil).

SCXRD Unit Cell Analysis

[a] Binary co-crystal crystal (nicotinamide: 3,5-dinitrobenzoic acid: MeOH, 2:2:2).

[b] Solvate (3,5-dinitrobenzoic acid: 1,4-dioxane).

nicotinamide: glutaric acid

nicotinamide : glutaric acid (A : B) plate 1													
Volume of Oil		200 nL											
Volume of Stock Solution (A : B)		2 : 1 (150 nL)				1 : 1 (150 nL)				1 : 2 (150 nL)			
Solvent		1	2	3	4	5	6	7	8	9	10	11	12
MeOH	A	2	3	3	4[a]	2	3	3	3	3	3	4	4[a]
MeOH	B	2	4[a]	4	3	3	3	4[a]	4	3	4[a]	4	4[a]
MeOH	C	2	3	3	3	3	4	4	4[a]	3	4[a]	4	4
MeOH	D	3	4	4	4[a]	3	4[a]	4	3	3	4	4*	4
DMF	E	3	3	3	3	3	3	3	3	3	3	3	3
DMF	F	3	4	4	4[a]	3	3	3	3	3	3	3	4[a]
DMF	G	3	3	3	3	3	1	1	3	3	1	1	3
DMF	H	3	3	3	3	4[a]	3	3	3	3	3	4[a]	3

nicotinamide : glutaric acid (A : B) plate 2													
Volume of Oil		200 nL											
Volume of Stock Solution (A : B)		2 : 1 (150 nL)				1 : 1 (150 nL)				1 : 2 (150 nL)			
Solvent		1	2	3	4	5	6	7	8	9	10	11	12
$MeNO_2$	A	3	3	3	3	3	3	3	3	3	3	3	3
$MeNO_2$	B	3	3	3	3	3	3	3	3	3	3	3	3
$MeNO_2$	C	3	3	3	3	3	3	3	3	3	3	4[a]	4
$MeNO_2$	D	3	3	3	3	3	3	3	3	3	3	3	3
1,4-Dioxane	E	3	3	3	3	3	3	3	3	3	3	3	3
1,4-Dioxane	F	4[a]	4	3	4[a]	4	3	4[a]	4	3	4	4[a]	3
1,4-Dioxane	G	3	4[a]	4	4	3	3	4[a]	4	3	3	3	4[a]
1,4-Dioxane	H	3	3	3	3	3	3	3	3	3	3	3	3

SCXRD Full Data Collection and Structure Refinement

* Binary co-crystal (nicotinamide: glutaric acid, 1:1; CSD refcode: NUKYEY) obtained from P1 D11 (MeOH, MO oil).

SCXRD Unit Cell Analysis

[a] Binary co-crystal (nicotinamide: glutaric acid, 1:1; CSD refcode: NUKYEY).

nicotinamide: 3-hydroxy-2-naphthoic acid

nicotinamide : 3-hydroxy-2-naphthoic acid (A : B) plate 1													
Volume of Oil		200 nL											
Volume of Stock Solution (A : B)		2 : 1 (150 nL)				1 : 1 (150 nL)				1 : 2 (150 nL)			
Solvent		1	2	3	4	5	6	7	8	9	10	11	12
MeOH	A	2	3	4	4[a]	2	4	3	4[a]	2	2	4	4*
MeOH	B	3	3	3	3	2	3	3	3	3	3	3	3
MeOH	C	3	3	3	4[a]	2	3	3	3	2	3	3	3
MeOH	D	2	3	3	3	2	3	4[a]	3	2	3	3	4[a]
DMF	E	3	3	3	3	1	3	3	3	1	3	3	3
DMF	F	1	2	2	1	1	1	3	3	1	3	3	3
DMF	G	3	1	1	1	1	1	1	1	1	1	1	1
DMF	H	3	1	3	3	1	1	3	3	1	3	3	3

nicotinamide : 3-hydroxy-2-naphthoic acid (A : B) plate 2													
Volume of Oil		200 nL											
Volume of Stock Solution (A : B)		2 : 1 (150 nL)				1 : 1 (150 nL)				1 : 2 (150 nL)			
Solvent		1	2	3	4	5	6	7	8	9	10	11	12
$MeNO_2$	A	3	2	3	3	3	3	3	3	3	3	3	3
$MeNO_2$	B	2	3	3	3	2	3	3	3	2	3	3	3
$MeNO_2$	C	2	2	3	3	3	3	3	3	2	3	3	3
$MeNO_2$	D	2	3	3	3	2	3	3	3	2	3	3	3
1,4-Dioxane	E	3	3	3	4[a]	3	4	4[a]	4	3	3	4[a]	3
1,4-Dioxane	F	3	3	3	3	3	3	3	3	3	3	3	3
1,4-Dioxane	G	3	3	2	2	3	2	3	2	3	2	2	2
1,4-Dioxane	H	3	3	4[a]	3	3	4	4[a]	4[a]	2	3	3	3

SCXRD Full Data Collection and Structure Refinement

* Binary co-crystal (nicotinamide: 3-hydroxy-2-naphthoic acid, 1:1; CSD refcode: ABULEQ) obtained from P1 A12 (MeOH, PDMSO oil).

SCXRD Unit Cell Analysis

[a] Binary co-crystal (nicotinamide: 3-hydroxy-2-naphthoic acid, 1:1; CSD refcode: ABULEQ).

nicotinamide: methyl gallate

nicotinamide : methyl gallate (A : B) plate 1													
**Volume of Oil**		**200 nL**											
**Volume of Stock Solution (A : B)**		**2 : 1 (150 nL)**				**1 : 1 (150 nL)**				**1 : 2 (150 nL)**			
**Solvent**		**1**	**2**	**3**	**4**	**5**	**6**	**7**	**8**	**9**	**10**	**11**	**12**
MeOH	**A**	2	4	4[a]	4	3	4[a]	3	4	2	4[a]	3	3
MeOH	**B**	2	2	3	3	3	2	3	3	2	3	3	2
MeOH	**C**	2	3	3	3	2	4[a]	4	3	2	3	4[a]	3
MeOH	**D**	3	4*	4	4	3	4	4[a]	4	2	4[a]	4	4
DMF	**E**	2	3	3	2	2	2	2	2	2	2	2	2
DMF	**F**	2	2	2	2	2	2	2	2	2	2	2	2
DMF	**G**	2	3	3	3	2	2	3	3	2	2	3	2
DMF	**H**	2	2	2	2	2	2	2	2	2	2	2	2

nicotinamide : methyl gallate (A : B) plate 2													
**Volume of Oil**		**200 nL**											
**Volume of Stock Solution (A : B)**		**2 : 1 (150 nL)**				**1 : 1 (150 nL)**				**1 : 2 (150 nL)**			
**Solvent**		**1**	**2**	**3**	**4**	**5**	**6**	**7**	**8**	**9**	**10**	**11**	**12**
$MeNO_2$	**A**	3	3	3	4[b]	2	2	2	2	2	3	3	3
$MeNO_2$	**B**	2	3	3	3	2	3	3	3	2	2	2	3
$MeNO_2$	**C**	2	3	3	3	2	4**	3	3	2	2	2	2
$MeNO_2$	**D**	2	3	3	3	2	3	2	2	2	2	2	3
1,4-Dioxane	**E**	2	3	3	3	2	3	3	3	2	2	2	2
1,4-Dioxane	**F**	2	3	3	2	2	3	3	2	2	3	3	3
1,4-Dioxane	**G**	2	2	3	3	3	3	3	3	3	3	3	3
1,4-Dioxane	**H**	2	3	3	3	2	3	3	3	2	3	3	3

SCXRD Full Data Collection and Structure Refinement

* Single-component crystal (methyl gallate; CSD refcode: ROMGAC) obtained from P1 D2 (MeOH, MO oil).

** Binary co-crystal (nicotinamide: methyl gallate, 1:1) obtained from P2 C6 ($MeNO_2$, FY oil).

SCXRD Unit Cell Analysis

[a] Single-component crystal (methyl gallate; CSD refcode: ROMGAC).

[b] Binary co-crystal (nicotinamide: methyl gallate, 1:1).

nicotinamide: quinol

nicotinamide : quinol (A : B) plate 1													
Volume of Oil		200 nL											
Volume of Stock Solution (A : B)		2 : 1 (150 nL)				1 : 1 (150 nL)				1 : 2 (150 nL)			
Solvent		1	2	3	4	5	6	7	8	9	10	11	12
MeOH	A	3	2	3	3	2	3	3	2	2	3	2	2
MeOH	B	2	2	4[a]	2	2	2	2	2	2	2	2	2
MeOH	C	2	4[a]	4	4	2	3	4[a]	4	2	4[a]	3	3
MeOH	D	2	4*	4	4	2	2	2	3	2	3	3	2
DMF	E	2	2	2	2	2	2	2	2	2	2	2	2
DMF	F	2	2	2	2	2	2	2	2	2	2	2	2
DMF	G	2	2	2	2	2	2	2	2	2	2	2	2
DMF	H	2	2	2	2	2	2	2	2	2	2	2	2

nicotinamide : quinol (A : B) plate 2													
Volume of Oil		200 nL											
Volume of Stock Solution (A : B)		2 : 1 (150 nL)				1 : 1 (150 nL)				1 : 2 (150 nL)			
Solvent		1	2	3	4	5	6	7	8	9	10	11	12
$MeNO_2$	A	2	3	3	2	2	4[a]	2	2	2	3	4[a]	4
$MeNO_2$	B	4	4[a]	4	4[a]	2	4[a]	2	2	4[a]	2	4[a]	4
$MeNO_2$	C	4[a]	4	4[a]	4	2	3	4[a]	2	2	4[a]	4	4
$MeNO_2$	D	4	4	4	4[a]	3	4[a]	2	2	2	2	2	2
1,4-Dioxane	E	2	4[a]	4	4	2	4	4[a]	3	2	4[a]	2	4
1,4-Dioxane	F	2	4	4[a]	4	2	4[a]	4	4	2	4	4[a]	3
1,4-Dioxane	G	2	4	2	4[a]	2	4[a]	2	2	2	4	4	4[a]
1,4-Dioxane	H	2	2	3	3	2	4[a]	2	2	2	4[a]	4	2

SCXRD Full Data Collection and Structure Refinement

* Binary co-crystal (nicotinamide: quinol, 2:0.5) obtained from P1 D2 (MeOH, MO).

SCXRD Unit Cell Analysis

[a] Binary co-crystal (nicotinamide: quinol, 2:0.5).

S4.4.2 Ternary Co-Crystallisation 96-Well Plate Readouts

toluic acid: isonicotinamide: 3,5-dinitrobenzoic acid

toluic acid : isonicotinamide : 3,5-dinitrobenzoic acid (A : B : C) plate 1													
Volume of Oil		200 nL											
Volume of Stock Solution (A : B : C)		2 : 1 : 1 (140 nL)				1 : 2 : 1 (140 nL)				1 : 1 : 2 (140 nL)			
Solvent		1	2	3	4	5	6	7	8	9	10	11	12
MeOH	A	4	2	3	3	2	2	3	3	4	2	3	3
MeOH	B	2	3	2	3	2	2	3	3	2	3	3	3
DMF	C	2	3	2	3	2	3	3	2	2	2	3	2
DMF	D	3	2	2	2	2	2	3	3	3	3	2	2
$MeNO_2$	E	4	4	4	4	3	4	3	4	4	4	3	4
$MeNO_2$	F	4*	4	3	3	4	4	3	4	4	4	4	4
1,4-Dioxane	G	2	2	3	3	3	3	4	3	3	3	4	4
1,4-Dioxane	H	2	2	4	4	3	3	4	3	4	4	4	4

toluic acid : isonicotinamide : 3,5-dinitrobenzoic acid (A : B : C) plate 2													
Volume of Oil		200 nL											
Volume of Stock Solution (A : B : C)		2 : 2 : 1 (140 nL)				2 : 1 : 2 (140 nL)				1 : 2 : 2 (140 nL)			
Solvent		1	2	3	4	5	6	7	8	9	10	11	12
MeOH	A	4	2	4	2	2	2	3	2	3	2	4	3
MeOH	B	2	2	4	3	2	2	3	3	2	4	4	4
DMF	C	2	3	3	2	2	3	3	3	3	2	3	3
DMF	D	2	3	3	2	2	3	2	2	2	2	2	3
$MeNO_2$	E	2	3	3	3	2	2	4	4	4	4	4	4
$MeNO_2$	F	3	4	4	4	4	3	3	4	4	4	4	4
1,4-Dioxane	G	2	3	2	3	2	3	3	4	2	3	3	3
1,4-Dioxane	H	2	4	3	3	3	3	3	3	3	3	3	3

toluic acid : isonicotinamide : 3,5-dinitrobenzoic acid (A : B : C) plate 3													
Volume of Oil		200 nL											
Volume of Stock Solution (A : B : C)		1 : 1 : 1 (140 nL)											
Solvent		1	2	3	4	5	6	7	8	9	10	11	12
MeOH	A	2	2	3	3	3	3	2	3	2	3	2	2
MeOH	B	2	2	2	2	2	2	2	3	2	3	3	4
DMF	C	2	2	3	3	2	2	2	2	2	3	3	3
DMF	D	2	3	2	3	3	3	2	2	2	3	3	3
$MeNO_2$	E	3	4	4	4	3	4	3	4	4	3	3	4
$MeNO_2$	F	4	4	4	4	4	4	4	4	4	4	4	4
1,4-Dioxane	G	2	2	2	2	2	2	2	4	4	4	4	3
1,4-Dioxane	H	2	4	3	4	4	3	2	2	2	3	4	4

SCXRD Full Data Collection and Structure Refinement

* Ternary co-crystal (toluic acid: isonicotinamide: 3,5-dinitrobenzoic acid, 1:1:1; CSD refcode: BUDZUV) obtained from P1 F1 ($MeNO_2$, FC-40 oil).

4,4’-bipyridine: orcinol: phenazine

4,4'-bipyridine : orcinol : phenazine (A : B : C) plate 1													
Volume of Oil		200 nL											
Volume of Stock Solution (A : B : C)		2 : 1 : 1 (140 nL)				1 : 2 : 1 (140 nL)				1 : 1 : 2 (140 nL)			
Solvent		1	2	3	4	5	6	7	8	9	10	11	12
MeOH	A	3	2	3	3	3	3	3	2	3	2	3	2
MeOH	B	2	2	2	2	2	2	2	2	3	2	2	3
DMF	C	2	2	2	2	2	4*	3	2	2	2	2	2
DMF	D	3	2	1	1	2	2	2	4	2	2	1	2
$MeNO_2$	E	3	3	3	3	3	2	4	4	3	4	4	4
$MeNO_2$	F	4	4	4	4	4	4	4[a]	4	4[a]	4	4	4
1,4-Dioxane	G	3	3	4	4	4	4	4	4**	4	2	4	4
1,4-Dioxane	H	4	4	4	4	4	4	4	4	4	4	4	4

4,4'-bipyridine : orcinol : phenazine (A : B : C) plate 2													
Volume of Oil		200 nL											
Volume of Stock Solution (A : B : C)		2 : 2 : 1 (140 nL)				2 : 1 : 2 (140 nL)				1 : 2 : 2 (140 nL)			
Solvent		1	2	3	4	5	6	7	8	9	10	11	12
MeOH	A	3	3	4	2	3	2	2	2	4	2	4	2
MeOH	B	2	2	2	2	2	2	2	2	2	2	2	2
DMF	C	2	4	1	4	4	4	3	4	4[a]	3	2	2
DMF	D	4	3	4	4	2	4***	3	2	2	2	2	3
$MeNO_2$	E	3	2	4[a]	4	2	3	4	4	3	3	4	4
$MeNO_2$	F	4	4	4	4	4	4	4	4	4	4	4	4
1,4-Dioxane	G	4	4	4	4	4	4	4	4	4	4	4	4
1,4-Dioxane	H	4	4	4	4	4	4	4	4	4	4	4[b]	4

4,4'-bipyridine : orcinol : phenazine (A : B : C) plate 3													
Volume of Oil		200 nL											
Volume of Stock Solution (A : B : C)		1 : 1 : 1 (140 nL)											
Solvent		1	2	3	4	5	6	7	8	9	10	11	12
MeOH	A	2	3	3	3	3	4	2	3	2	2	2	2
MeOH	B	2	2	2	2	2	2	2	2	2	2	2	2
DMF	C	2	4[a]	2	2	2	2	2	2	4	2	2	2
DMF	D	2	3	3	3	3	3	3	3	3	3	3	3
$MeNO_2$	E	3	3	3	2	2	4	4	4[a]	4	4	4	4
$MeNO_2$	F	3	4	4	3	3	3	3	3	3	3	4	4
1,4-Dioxane	G	3	4	3	3	3	3	3	4	4	4	4	4
1,4-Dioxane	H	4	4	4	4	4	4	4	4	4	4	4	4

SCXRD Full Data Collection and Structure Refinement

* Binary co-crystal (4,4’-bipyridine: orcinol, 1.5:1; CSD refcode: UBUJIM) obtained from P1 C6 (DMF, FY).
** Binary co-crystal solvate (orcinol: phenazine: 1,4-dioxane, 1:1:0.5) obtained from P1 G8 (1,4-dioxane, FY).
*** Ternary co-crystal (4,4’-bipyridine: orcinol: phenazine, 1:1:0.5; CSD refcode: UBUKEJ) obtained from P2 D6 (DMF, MO).

SCXRD Unit Cell Analysis

[a] Binary co-crystal (4,4’-bipyridine: orcinol, 1.5:1; CSD refcode: UBUJIM).
[b] Binary co-crystal (orcinol: phenazine: 1,4-dioxane, 1:1:0.5).

nicotinamide: fumaric acid: isoniazid

nicotinamide : fumaric acid : isoniazid (A : B : C) plate 1													
Volume of Oil		200 nL											
Volume of Stock Solution (A : B : C)		2 : 1 : 1 (140 nL)				1 : 2 : 1 (140 nL)				1 : 1 : 2 (140 nL)			
Solvent		1	2	3	4	5	6	7	8	9	10	11	12
MeOH	A	3	2	2	2	2	2	2	2	2	2	2	2
MeOH	B	2	2	2	2	2	2	2	2	2	2	2	2
DMF	C	2	2	1	2	3	4	2	1	2	1	2	2
DMF	D	4	3	1	4	2	2	2	2	4	3	3	2
$MeNO_2$	E	3	3	3	3	3	3	3	3	3	3	3	3
$MeNO_2$	F	3	3	3	3	3	2	2	2	3	4	4	3
1,4-Dioxane	G	2	2	2	2	2	2	2	2	2	2	2	2
1,4-Dioxane	H	2	2	2	2	2	2	2	2	2	2	2	2

nicotinamide : fumaric acid : isoniazid (A : B : C) plate 2													
Volume of Oil		200 nL											
Volume of Stock Solution (A : B : C)		2 : 2 : 1 (140 nL)				2 : 1 : 2 (140 nL)				1 : 2 : 2 (140 nL)			
Solvent		1	2	3	4	5	6	7	8	9	10	11	12
MeOH	A	3	3	3	3	3	2	2	2	2	2	2	2
MeOH	B	2	2	4	2	2	2	2	2	2	2	4	3
DMF	C	2	2	3	2	1	1	3	3	1	4	4	2
DMF	D	3	2	4	4	1	1	4	4	3	4	4*	4
$MeNO_2$	E	2	2	2	3	3	3	3	3	3	3	3	3
$MeNO_2$	F	3	3	3	2	4	2	2	2	2	4	4	3
1,4-Dioxane	G	2	2	2	2	2	2	2	2	2	2	2	2
1,4-Dioxane	H	2	2	3	2	2	2	2	2	2	2	2	2

nicotinamide : fumaric acid : isoniazid (A : B : C) plate 3													
Volume of Oil		200 nL											
Volume of Stock Solution (A : B : C)		1 : 1 : 1 (140 nL)											
Solvent		1	2	3	4	5	6	7	8	9	10	11	12
MeOH	A	2	2	2	2	2	2	2	2	2	2	2	2
MeOH	B	2	2	2	2	2	2	2	2	2	2	2	2
DMF	C	2	4	4	3	3	1	3	2	2	4	4	2
DMF	D	1	2	2	2	1	1	4	1	2	4	1	2
$MeNO_2$	E	2	3	3	2	2	2	2	2	2	2	2	2
$MeNO_2$	F	2	2	2	2	2	4	2	2	2	2	2	2
1,4-Dioxane	G	2	2	2	2	2	2	2	2	2	2	2	2
1,4-Dioxane	H	2	2	2	2	4	2	2	2	2	2	2	2

SCXRD Full Data Collection and Structure Refinement

* Ternary co-crystal (nicotinamide: fumaric acid: isoniazid, 1:1:1; CSD refcode: BICQEL) obtained from P2 D11 (DMF, FC-40 oil).

tetramethylpyrazine: 2,2’-bipyridine: 2-chlororesorcinol

tetramethylpyrazine : 2,2'-bipyridine : 2-chlororesorcinol (A : B : C) plate 1													
**Volume of Oil**		**200 nL**											
**Volume of Stock Solution (A : B : C)**		**2 : 1 : 1 (140 nL)**				**1 : 2 : 1 (140 nL)**				**1 : 1 : 2 (140 nL)**			
**Solvent**		**1**	**2**	**3**	**4**	**5**	**6**	**7**	**8**	**9**	**10**	**11**	**12**
MeOH	**A**	4	3	2	4	4	4	4	4	4	4	4	4
MeOH	**B**	2	2	2	4	4	4	4	4	2	2	4	4
DMF	**C**	2	1	1	1	1	1	1	1	1	1	1	1
DMF	**D**	1	1	1	1	1	1	4	1	1	1	1	4
$MeNO_2$	**E**	2	2	4	4	3	4	4	4	4	4	4	4
$MeNO_2$	**F**	4	4	4	4	4	4	4	4	4	4	4	4
1,4-Dioxane	**G**	2	4	4	4	4	4	4	4	4	4	4	4
1,4-Dioxane	**H**	2	2	4	4	4	4	4	4	4	4	4	4

tetramethylpyrazine : 2,2'-bipyridine : 2-chlororesorcinol (A : B : C) plate 2													
**Volume of Oil**		**200 nL**											
**Volume of Stock Solution (A : B : C)**		**2 : 2 : 1 (140 nL)**				**2 : 1 : 2 (140 nL)**				**1 : 2 : 2 (140 nL)**			
**Solvent**		**1**	**2**	**3**	**4**	**5**	**6**	**7**	**8**	**9**	**10**	**11**	**12**
MeOH	**A**	4	4	1	1	3	3	4	4	4	2	2	4
MeOH	**B**	2	2	4	4	3	3	4	4	2	2	4	4
DMF	**C**	4	1	1	1	1	1	1	1	1	1	1	1
DMF	**D**	2	1	1	4	1	1	1	1	1	1	1	1
$MeNO_2$	**E**	4	4	4	4	4	4	4	4	4	4	4	4
$MeNO_2$	**F**	4	4	4	4	4*	4	4	4	2	4	4	4
1,4-Dioxane	**G**	4	4	4	4	4	4	4	4	4	4	4	4
1,4-Dioxane	**H**	2	2	4	4	2	2	4	4	2	2	4	4

tetramethylpyrazine : 2,2'-bipyridine : 2-chlororesorcinol (A : B : C) plate 3													
**Volume of Oil**		**200 nL**											
**Volume of Stock Solution (A : B : C)**		**1 : 1 : 1 (140 nL)**											
**Solvent**		**1**	**2**	**3**	**4**	**5**	**6**	**7**	**8**	**9**	**10**	**11**	**12**
MeOH	**A**	2	4	4	4	4	4	3	4	4	4	4	4
MeOH	**B**	2	2	2	2	2	2	2	4	4	4	4	4
DMF	**C**	1	1	1	1	1	1	1	1	1	1	1	1
DMF	**D**	1	1	1	1	1	1	1	1	1	1	1	1
$MeNO_2$	**E**	3	3	3	3	3	3	4	4	4	4**	4	4
$MeNO_2$	**F**	4	4	4	4	4	4	4	4	4	4	4	4
1,4-Dioxane	**G**	4	4	4	4	4	4	4	4	4	4	4	4
1,4-Dioxane	**H**	2	4	4	4	4	4	2	4	4	4	4	4

SCXRD Full Data Collection and Structure Refinement

* Ternary co-crystal (tetramethylpyrazine: 2,2’-bipyridine: 2-chlororesorcinol, 1:0.5:1) obtained from P2 F5 ($MeNO_2$, FC-40 oil).

** Ternary co-crystal (tetramethylpyrazine: 2,2’-bipyridine: 2-chlororesorcinol, 0.5:0.5:1; CSD refcode: BESNOF) obtained from P3 E10 ($MeNO_2$, FY oil).

4,4’-bipyridine: methyl gallate: 2-chlororesorcinol

4,4'-bipyridine : methyl gallate : 2-chlororesorcinol (A : B : C) plate 1													
Volume of Oil		200 nL											
Volume of Stock Solution (A : B : C)		2 : 1 : 1 (140 nL)				1 : 2 : 1 (140 nL)				1 : 1 : 2 (140 nL)			
Solvent		1	2	3	4	5	6	7	8	9	10	11	12
MeOH	A	3	2	3	3	4^a	3	3	4	4	4*	4^a	4
MeOH	B	2	2	4^a	2	4	4^a	3	3	4^a	3	3	4
DMF	C	3	3	3	1	3	3	3	4^b	4	1	4^b	4
DMF	D	3	1	1	1	3	3	1	1	1	1	1	4^b
$MeNO_2$	E	3	3	3	3	3	3	3	3	3	3	3	3
$MeNO_2$	F	3	3	3	3	3	3	3	3	3	3	4^c	3
1,4-Dioxane	G	3	3	3	3	3	3	3	4^d	4^d	4^d	4	4
1,4-Dioxane	H	3	4^e	4^e	4	4^e	4^e	3	3	3	3	3	3

4,4'-bipyridine : methyl gallate : 2-chlororesorcinol (A : B : C) plate 2													
Volume of Oil		200 nL											
Volume of Stock Solution (A : B : C)		2 : 2 : 1 (140 nL)				2 : 1 : 2 (140 nL)				1 : 2 : 2 (140 nL)			
Solvent		1	2	3	4	5	6	7	8	9	10	11	12
MeOH	A	3	2	4^a	2	4^a	3	3	3	4^a	4^a	3	3
MeOH	B	3	3	4^a	3	3	3	3	3	4^a	3	3	4
DMF	C	3	3	3	3	3	3	3	3	3	4	4^b	4
DMF	D	2	1	1	1	2	2	1	1	1	1	1	1
$MeNO_2$	E	2	3	3	3	3	3	3	3	3	3	3	3
$MeNO_2$	F	3	3	3	3	4^c	3	3	4^c	3	4^c	4	3
1,4-Dioxane	G	3	3	3	3	3	3	3	3	3	4^e	4	3
1,4-Dioxane	H	3	3	3	3	3	3	3	3	3	3	3	3

4,4'-bipyridine : methyl gallate : 2-chlororesorcinol (A : B : C) plate 3													
Volume of Oil		200 nL											
Volume of Stock Solution (A : B : C)		1 : 1 : 1 (140 nL)											
Solvent		1	2	3	4	5	6	7	8	9	10	11	12
MeOH	A	2	3	3	4^a	3	3	2	3	3	3	3	3
MeOH	B	2	2	2	2	2	2	2	2	3	3	3	2
DMF	C	2	2	4	4^b	4	3	2	4	4	4^b	4	4
DMF	D	3	4	4^b	4	4	4	2	4	4	4^b	4	4
$MeNO_2$	E	2	3	3	3	3	3	3	3	4	4^c	3	4
$MeNO_2$	F	2	3	3	3	3	3	3	4^c	4	4	4	4
1,4-Dioxane	G	2	2	2	2	2	2	2	4	4	4^e	4	4
1,4-Dioxane	H	2	2	2	2	2	2	2	4^e	4	4	4	4

SCXRD Full Data Collection and Structure Refinement

* Ternary co-crystal (4,4’-bipyridine: methyl gallate: 2-chlororesocinol: H_2O, 3:2:1:2) obtained from P1 A10 (MeOH, FY oil).

SCXRD Unit Cell Analysis

[a] Ternary co-crystal hydrate (4,4’-bipyridine: methyl gallate: 2-chlororesocinol: H_2O, 3:2:1:2).
[b] Binary co-crystal solvate hydrate (4,4’-bipyridine: methyl gallate: DMF: H_2O, 3:2:1:2)
[c] Binary co-crystal solvate hydrate (4,4’-bipyridine: methyl gallate: $MeNO_2$: H_2O, 3:2:2:2).
[d] Binary co-crystal hydrate (4,4’-bipyridine: methyl gallate: H_2O, 2:2:3).
[e] Single-component crystal (methyl gallate, CSD refcode: ROMGAC).

caffeine: 3,5-dinitrobenzoic acid: 2-methylresorcinol

caffeine : 3,5-dinitrobenzoic acid: 2-methylresorcinol (A : B : C) plate 1													
**Volume of Oil**		**200 nL**											
**Volume of Stock Solution (A : B : C)**		**2 : 1 : 1 (140 nL)**				**1 : 2 : 1 (140 nL)**				**1 : 1 : 2 (140 nL)**			
**Solvent**		**1**	**2**	**3**	**4**	**5**	**6**	**7**	**8**	**9**	**10**	**11**	**12**
MeOH	**A**	3	3	3	3	3	3	3	3	3	3	3	3
MeOH	**B**	3	3	3	3	3	3	3	3	3	3	3	3
DMF	**C**	3	3	3	3	3	3	3	3	3	3	3	3
DMF	**D**	3	3	3	3	3	3	3	3	3	3	3	3
$MeNO_2$	**E**	3	3	4[a]	4[a]	4[a]	3	3	3	3	4[a]	3	3
$MeNO_2$	**F**	3	3	4[a]	3	3	3	3	3	3	3	3	3
1,4-Dioxane	**G**	3	3	3	3	3	3	3	3	3	3	3	3
1,4-Dioxane	**H**	3	4[b]	4[b]	3	3	3	3	3	3	4[b]	3	4

caffeine : 3,5-dinitrobenzoic acid: 2-methylresorcinol (A : B : C) plate 2													
**Volume of Oil**		**200 nL**											
**Volume of Stock Solution (A : B : C)**		**2 : 2 : 1 (140 nL)**				**2 : 1 : 2 (140 nL)**				**1 : 2 : 2 (140 nL)**			
**Solvent**		**1**	**2**	**3**	**4**	**5**	**6**	**7**	**8**	**9**	**10**	**11**	**12**
MeOH	**A**	3	3	3	3	3	3	3	3	3	3	3	3
MeOH	**B**	3	3	3	3	3	3	3	3	3	3	3	3
DMF	**C**	3	3	3	3	3	3	3	3	3	3	3	3
DMF	**D**	3	3	3	3	3	3	3	3	3	3	3	3
$MeNO_2$	**E**	4[a]	4[a]	3	3	3	3	3	3	3	3	3	3
$MeNO_2$	**F**	4[a]	3	3	3	3	3	3	3	3	3	3	3
1,4-Dioxane	**G**	3	3	3	3	3	3	3	3	3	3	3	3
1,4-Dioxane	**H**	3	4[b]	3	3	3	3	3	3	4[b]	3	3	3

caffeine : 3,5-dinitrobenzoic acid: 2-methylresorcinol (A : B : C) plate 3													
**Volume of Oil**		**200 nL**											
**Volume of Stock Solution (A : B : C)**		**1 : 1 : 1 (140 nL)**											
**Solvent**		**1**	**2**	**3**	**4**	**5**	**6**	**7**	**8**	**9**	**10**	**11**	**12**
MeOH	**A**	2	3	3	3	3	3	2	3	3	3	3	3
MeOH	**B**	2	3	3	3	3	3	2	3	3	3	3	3
DMF	**C**	3	3	3	3	3	3	3	3	3	3	3	3
DMF	**D**	3	3	3	3	3	3	3	3	3	3	3	3
$MeNO_2$	**E**	3	3	3	3	3	4*	3	3	4[a]	3	4[a]	3
$MeNO_2$	**F**	2	3	3	3	4[a]	3	3	3	3	4	4[a]	4
1,4-Dioxane	**G**	2	3	3	3	3	3	3	4[b]	3	3	3	3
1,4-Dioxane	**H**	2	4	4[b]	3	4	3	2	3	3	3	3	4[b]

SCXRD Full Data Collection and Structure Refinement

* Ternary co-crystal hydrate (caffeine: 3,5-dinitrobenzoic acid: 2-methylresorcinol: H_2O, 1:1:2:1) obtained from P3 E6 ($MeNO_2$, PDMSO oil).

SCXRD Unit Cell Analysis

[a] Ternary co-crystal hydrate (caffeine: 3,5-dinitrobenzoic acid: 2-methylresorcinol: H_2O, 1:1:2:1).

[b] Solvate (3,5-dinitrobenzoic acid: 1,4-dioxane).

nicotinamide: 3,5-dinitrobenzoic acid: glutaric acid

nicotinamide : 3,5-dinitrobenzoic acid : glutaric acid (A : B : C) plate 1													
Volume of Oil		200 nL											
Volume of Stock Solution (A : B : C)		2 : 1 : 1 (140 nL)				1 : 2 : 1 (140 nL)				1 : 1 : 2 (140 nL)			
Solvent		1	2	3	4	5	6	7	8	9	10	11	12
MeOH	A	2	2	2	1	2	3	1	2	3	3	1	2
MeOH	B	1	3	3	3	3	3	3	4[a]	4[a]	3	3	3
DMF	C	4[b]	1	1	1	3	3	1	1	3	4[b]	1	4
DMF	D	1	1	1	1	2	3	1	1	4[a]	3	4[c]	4
$MeNO_2$	E	1	1	1	1	1	3	3	3	2	3	3	1
$MeNO_2$	F	1	1	1	1	3	3	3	3	3	3	3	1
1,4-Dioxane	G	3	3	1	1	3	2	2	2	3	3	1	1
1,4-Dioxane	H	1	3	3	2	3	3	4[b]	3	1	1	3	1

nicotinamide : 3,5-dinitrobenzoic acid : glutaric acid (A : B : C) plate 2													
Volume of Oil		200 nL											
Volume of Stock Solution (A : B : C)		2 : 2 : 1 (140 nL)				2 : 1 : 2 (140 nL)				1 : 2 : 2 (140 nL)			
Solvent		1	2	3	4	5	6	7	8	9	10	11	12
MeOH	A	3	1	2	2	2	2	3	3	1	3	1	1
MeOH	B	3	4[b]	3	3	3	3	2	3	1	4[b]	4[b]	2
DMF	C	2	1	1	1	1	1	1	1	1	1	1	1
DMF	D	1	1	1	1	1	1	1	1	1	1	1	3
$MeNO_2$	E	3	2	3	1	3	3	3	3	1	1	1	1
$MeNO_2$	F	3	3	3	3	3	3	3	4	4	4	4[b]	4[b]
1,4-Dioxane	G	4	4[c]	1	1	3	2	1	1	4**	3	1	1
1,4-Dioxane	H	3	3	4[b]	4	3	3	3	3	3	3	3	4[c]

nicotinamide : 3,5-dinitrobenzoic acid : glutaric acid (A : B : C) plate 3													
Volume of Oil		200 nL											
Volume of Stock Solution (A : B : C)		1 : 1 : 1 (140 nL)											
Solvent		1	2	3	4	5	6	7	8	9	10	11	12
MeOH	A	2	2	2	2	2	2	2	2	2	1	2	1
MeOH	B	3	1	1	4[a]	4[a]	1	3	3	3	3	3	3
DMF	C	4[a]	1	1	1	1	1	1	1	1	1	1	1
DMF	D	2	1	1	1	1	1	1	1	1	1	4[a]	4[a]
$MeNO_2$	E	4[d]	4*	3	4[a]	4[a]	1	4[a]	1	3	1	3	1
$MeNO_2$	F	3	4	4[b]	4	4	3	4	4	4[a]	4[b]	4	4
1,4-Dioxane	G	3	4	4[b]	4	2	4	2	1	1	1	1	1
1,4-Dioxane	H	3	3	3	3	3	1	3	3	3	4[b]	4	4

SCXRD Full Data Collection and Structure Refinement

* Ternary co-crystal (nicotinamide: 3,5-dinitrobenzoic acid: glutaric acid, 1:1:1) obtained from P3 E2 ($MeNO_2$, PDMSO oil).

** Single-component crystal (glutaric acid; CSD refcode: GLURAC02) obtained from P2 G9 (1,4-dioxane, PDMSO oil).

SCXRD Unit Cell Analysis

[a] Binary co-crystal (nicotinamide: glutaric acid, 1:1; CSD refcode: NUKYEY).
[b] Single-component crystal (3,5-dinitrobenzoic acid; CSD refcode: CUKCAM).
[c] Single-component crystal (glutaric acid; CSD refcode: GLURAC02).
[d] Ternary co-crystal (nicotinamide: 3,5-dinitrobenzoic acid: glutaric acid, 1:1:1).

nicotinamide: 3,5-dinitrobenzoic acid: tetramethylpyrazine

nicotinamide : 3,5-dinitrobenzoic acid : tetramethylpyrazine (A : B : C) plate 1													
Volume of Oil		200 nL											
Volume of Stock Solution (A : B : C)		2 : 1 : 1 (140 nL)				1 : 2 : 1 (140 nL)				1 : 1 : 2 (140 nL)			
Solvents		1	2	3	4	5	6	7	8	9	10	11	12
MeOH	A	3	4[a]	3	3	4	4*	4	3	4[a]	4	4	4
MeOH	B	3	3	3	4[a]	3	2	2	4	3	3	3	4[b]
DMF	C	4[b]	4[b]	1	1	1	1	1	1	4	1	1	3
DMF	D	4	1	1	1	1	1	1	1	4[b]	1	1	4[b]
$MeNO_2$	E	3	3	3	4	3	3	2	3	3	3	3	3
$MeNO_2$	F	3	3	3	3	3	3	3	3	3	3	3	3
1,4-dioxane	G	3	3	3	3	3	3	3	3	3	3	3	3
1,4-dioxane	H	4	3	3	3	4[c]	3	3	3	2	4	4[c]	3

nicotinamide : 3,5-dinitrobenzoic acid : tetramethylpyrazine (A : B : C) plate 2													
Volume of Oil		200 nL											
Volume of Stock Solution (A : B : C)		2 : 2 : 1 (140 nL)				2 : 1 : 2 (140 nL)				1 : 2 : 2 (140 nL)			
Solvents		1	2	3	4	5	6	7	8	9	10	11	12
MeOH	A	3	3	3	3	3	3	3	3	3	3	3	3
MeOH	B	3	3	3	3	3	3	3	3	3	2	3	3
DMF	C	1	1	1	1	1	1	1	1	1	1	1	1
DMF	D	1	1	1	1	1	1	1	1	1	1	4[b]	1
$MeNO_2$	E	3	3	3	3	3	3	3	3	3	3	3	3
$MeNO_2$	F	3	3	3	3	3	3	3	3	3	3	3	3
1,4-dioxane	G	2	2	1	1	2	2	4[c]	4	3	3	1	4[c]
1,4-dioxane	H	3	4	3	4[c]	3	4	3	4[c]	4	4	4	3

nicotinamide : 3,5-dinitrobenzoic acid : tetramethylpyrazine (A : B : C) plate 3													
Volume of Oil		200 nL											
Volume of Stock Solution (A : B : C)		1 : 1 : 1 (140 nL)											
Solvents		1	2	3	4	5	6	7	8	9	10	11	12
MeOH	A	2	3	3	3	3	3	2	3	3	3	3	3
MeOH	B	2	3	3	3	3	3	2	3	3	3	3	3
DMF	C	4	1	1	1	1	1	1	1	1	1	1	1
DMF	D	4[b]	1	4[b]	1	1	1	1	1	1	1	1	1
$MeNO_2$	E	2	3	3	3	3	3	2	3	3	3	3	3
$MeNO_2$	F	2	3	3	3	3	3	2	3	3	3	3	3
1,4-dioxane	G	2	3	3	3	3	3	2	4[c]	3	3	4[c]	4
1,4-dioxane	H	3	3	3	3	3	3	3	3	3	3	3	4

SCXRD Full Data Collection and Structure Refinement

* Ternary co-crystal hydrate (nicotinamide: 3,5-dinitrobenzoic acid: tetramethylpyrazine: H_2O, 1:1:0.5:1) obtained from P1 A6 (MeOH, FY).

SCXRD Unit Cell Analysis

[a] Ternary co-crystal hydrate (nicotinamide: 3,5-dinitrobenzoic acid: tetramethylpyrazine: H_2O, 1:1:0.5:1).

[b] Binary co-crystal (nicotinamide: glutaric acid, 1:1; CSD refcode: NUKYEY).

[c] Single-component crystal (3,5-dinitrobenzoic acid; CSD refcode: CUKCAM).

nicotinamide: quinol: isonicotinamide

nicotinamide : quinol : isonicotinamide (A : B : C) plate 1													
Volume of Oil		200 nL											
Volume of Stock Solution (A : B : C)		2 : 1 : 1 (140 nL)				1 : 2 : 1 (140 nL)				1 : 1 : 2 (140 nL)			
Solvent		1	2	3	4	5	6	7	8	9	10	11	12
MeOH	A	4[a]	3	3	3	3	3	3	3	3	3	3	3
MeOH	B	3	3	1	3	1	3	3	3	3	3	3	3
DMF	C	1	1	4[a]	1	1	1	1	1	1	1	1	1
DMF	D	1	1	1	1	1	1	1	1	1	1	1	1
$MeNO_2$	E	3	3	3	3	3	3	3	3	3	3	3	3
$MeNO_2$	F	3	3	1	3	1	3	3	3	1	3	1	3
1,4-Dioxane	G	4	4[a]	4[a]	4	4[a]	4[a]	4	4	4[a]	4	4	4[a]
1,4-Dioxane	H	1	3	1	3	1	3	1	4[a]	1	3	1	4[a]

nicotinamide : quinol : isonicotinamide (A : B : C) plate 2													
Volume of Oil		200 nL											
Volume of Stock Solution (A : B : C)		2 : 2 : 1 (140 nL)				2 : 1 : 2 (140 nL)				1 : 2 : 2 (140 nL)			
Solvent		1	2	3	4	5	6	7	8	9	10	11	12
MeOH	A	3	3	3	3	3	3	3	3	3	3	3	3
MeOH	B	1	3	1	3	3	3	1	3	1	3	3	3
DMF	C	4[a]	1	1	1	1	1	4[a]	1	4[a]	1	4	1
DMF	D	1	1	1	1	1	1	1	1	1	1	1	1
$MeNO_2$	E	3	3	1	3	3	3	3	3	3	3	3	3
$MeNO_2$	F	1	3	1	3	3	3	1	3	1	3	3	3
1,4-Dioxane	G	4[a]	4[a]	3	4	4[a]	4[a]	3	4	4[a]	4[a]	4	4
1,4-Dioxane	H	1	4[a]	1	3	1	4[a]	1	3	1	4[a]	1	4[a]

nicotinamide : quinol : isonicotinamide (A : B : C) plate 3													
Volume of Oil		200 nL											
Volume of Stock Solution (A : B : C)		1 : 1 : 1 (140 nL)											
Solvent		1	2	3	4	5	6	7	8	9	10	11	12
MeOH	A	3	3	3	1	4[a]	3	3	3	1	1	1	3
MeOH	B	3	3	3	3	3	1	1	3	3	3	3	3
DMF	C	1	1	4[a]	4	4	4	1	1	3	1	1	3
DMF	D	1	1	1	1	1	1	1	1	1	1	1	3
$MeNO_2$	E	3	1	3	3	3	3	3	2	1	1	3	1
$MeNO_2$	F	3	1	3	1	1	4[a]	3	3	3	3	3	1
1,4-Dioxane	G	4[a]	3	3	3	3	3	3	1	1	1	1	1
1,4-Dioxane	H	3	4[a]	1	4	1	1	4[a]	4[a]	3	4	3	3

SCXRD Unit Cell Analysis

[a] Binary co-crystal (nicotinamide: quinol, 2: 0.5).

nicotinamide: quinol: nicotinic acid

nicotinamide : quinol : nicotinic acid (A : B : C) plate 1													
Volume of Oil		200 nL											
Volume of Stock Solution (A : B : C)		2 : 1 : 1 (140 nL)				1 : 2 : 1 (140 nL)				1 : 1 : 2 (140 nL)			
Solvent		1	2	3	4	5	6	7	8	9	10	11	12
MeOH	A	4[a]	3	3	2	2	2	2	2	2	2	2	2
MeOH	B	3	3	2	2	2	2	3	3	3	4[a]	3	3
DMF	C	1	1	1	1	1	3	1	3	1	1	1	1
DMF	D	3	3	3	3	3	3	3	2	3	2	3	3
$MeNO_2$	E	3	4[a]	3	4	3	4[a]	3	4	3	4[a]	4[a]	4
$MeNO_2$	F	3	3	3	3	3	3	3	3	3	3	3	3
1,4-Dioxane	G	2	2	3	3	3	3	3	2	3	3	2	2
1,4-Dioxane	H	2	3	2	3	2	3	2	3	2	3	2	3

nicotinamide : quinol : nicotinic acid (A : B : C) plate 2													
Volume of Oil		200 nL											
Volume of Stock Solution (A : B : C)		2 : 2 : 1 (140 nL)				2 : 1 : 2 (140 nL)				1 : 2 : 2 (140 nL)			
Solvent		1	2	3	4	5	6	7	8	9	10	11	12
MeOH	A	4	3	4[a]	3	3	3	4[a]	3	3	3	4[a]	3
MeOH	B	3	3	3	2	3	3	2	3	3	3	3	4[a]
DMF	C	1	1	1	3	1	1	1	3	1	3	1	3
DMF	D	1	1	3	3	1	3	2	1	1	1	3	3
$MeNO_2$	E	3	4[a]	3	4[a]	3	3	3	4[a]	3	3	3	4[a]
$MeNO_2$	F	3	3	3	3	3	3	3	3	3	3	3	3
1,4-Dioxane	G	3	2	3	2	3	2	3	2	3	2	2	2
1,4-Dioxane	H	2	4[b]	2	3	2	3	2	3	2	3	3	4[b]

nicotinamide : quinol : nicotinic acid (A : B : C) plate 3													
Volume of Oil		200 nL											
Volume of Stock Solution (A : B : C)		1 : 1 : 1 (140 nL)											
Solvent		1	2	3	4	5	6	7	8	9	10	11	12
MeOH	A	3	3	4[a]	2	2	4[a]	3	1	2	2	3	3
MeOH	B	2	3	3	2	2	2	3	2	4[a]	4	2	3
DMF	C	3	1	1	1	1	1	1	1	1	2	3	3
DMF	D	3	2	1	3	3	1	3	1	1	3	3	4[a]
$MeNO_2$	E	3	3	3	3	3	3	3	1	3	3	2	2
$MeNO_2$	F	3	3	4[a]	4	4	4[a]	3	3	3	4[a]	4	3
1,4-Dioxane	G	3	2	3	3	3	3	3	1	3	3	3	3
1,4-Dioxane	H	3	2	2	2	2	2	3	3	4*	4[b]	3	2

SCXRD Full Data Collection and Structure Refinement

* Binary co-crystal (nicotinamide: quinol, 2: 0.5, polymorph II) obtained from P3 H9 (1,4-dioxane, FY oil).

SCXRD Unit Cell Analysis

[a] Binary co-crystal (nicotinamide: quinol, 2: 0.5, polymorph I).

[b] Binary co-crystal (nicotinamide: quinol, 2: 0.5, polymorph II).

nicotinamide: quinol: benzoic acid

nicotinamide : quinol : benzoic acid (A : B : C) plate 1													
**Volume of Oil**		**200 nL**											
**Volume of Stock Solution (A : B : C)**		**2 : 1 : 1 (140 nL)**				**1 : 2 : 1 (140 nL)**				**1 : 1 : 2 (140 nL)**			
**Solvent**		**1**	**2**	**3**	**4**	**5**	**6**	**7**	**8**	**9**	**10**	**11**	**12**
MeOH	**A**	2	3	3	3	3	3	2	3	4[a]	3	1	2
MeOH	**B**	3	3	3	3	3	3	3	4[a]	4	4	1	3
DMF	**C**	2	2	2	2	2	2	2	2	2	2	2	2
DMF	**D**	2	2	2	2	2	2	2	2	2	2	2	2
$MeNO_2$	**E**	3	2	3	2	3	3	3	3	2	2	3	2
$MeNO_2$	**F**	2	3	1	3	3	3	2	2	2	2	2	3
1,4-Dioxane	**G**	2	3	2	2	2	3	2	2	2	3	2	3
1,4-Dioxane	**H**	2	3	1	3	2	3	2	3	1	3	2	3

nicotinamide : quinol : benzoic acid (A : B : C) plate 2													
**Volume of Oil**		**200 nL**											
**Volume of Stock Solution (A : B : C)**		**2 : 2 : 1 (140 nL)**				**2 : 1 : 2 (140 nL)**				**1 : 2 : 2 (140 nL)**			
**Solvent**		**1**	**2**	**3**	**4**	**5**	**6**	**7**	**8**	**9**	**10**	**11**	**12**
MeOH	**A**	3	1	3	3	2	2	3	3	2	3	3	3
MeOH	**B**	3	4[a]	3	3	3	3	2	3	3	4[a]	4[a]	4
DMF	**C**	2	2	2	2	2	2	2	2	2	2	2	2
DMF	**D**	2	2	2	2	2	2	2	2	2	2	2	2
$MeNO_2$	**E**	3	3	3	3	3	3	3	2	3	3	3	3
$MeNO_2$	**F**	2	3	3	3	2	3	2	3	3	3	2	3
1,4-Dioxane	**G**	2	3	2	3	2	3	2	3	3	3	3	3
1,4-Dioxane	**H**	1	3	2	3	3	3	2	3	2	3	3	3

nicotinamide : quinol : benzoic acid (A : B : C) plate 3													
**Volume of Oil**		**200 nL**											
**Volume of Stock Solution (A : B : C)**		**1 : 1 : 1 (140 nL)**											
**Solvent**		**1**	**2**	**3**	**4**	**5**	**6**	**7**	**8**	**9**	**10**	**11**	**12**
MeOH	**A**	2	3	3	3	3	3	2	3	2	4[a]	4[a]	3
MeOH	**B**	2	2	2	3	4[a]	3	2	3	3	3	3	3
DMF	**C**	2	2	2	2	2	2	2	2	2	2	2	2
DMF	**D**	2	2	2	2	2	2	2	2	2	2	2	2
$MeNO_2$	**E**	2	3	3	3	2	3	2	3	2	2	2	2
$MeNO_2$	**F**	2	2	3	3	3	3	2	3	2	3	3	3
1,4-Dioxane	**G**	2	2	2	2	2	2	2	2	1	1	2	1
1,4-Dioxane	**H**	2	3	3	3	3	3	2	3	3	4	4[b]	4*

SCXRD Full Data Collection and Structure Refinement

* Ternary co-crystal (nicotinamide: quinol: benzoic acid, 1: 0.5: 1) obtained from P3 H12 (1,4-Dioxane, MO).

SCXRD Unit Cell Analysis

[a] Single-component crystal (quinol; CSD refcode: HYQUIN04).

[b] Ternary co-crystal (nicotinamide: quinol: benzoic acid, 1: 0.5: 1).

caffeine: quinol: 4-hydroxybenzoic acid

caffeine : quinol : 4-hydroxybenzoic acid (A : B : C) plate 1													
Volume of Oil		200 nL											
Volume of Stock Solution (A : B : C)		2 : 1 : 1 (140 nL)				1 : 2 : 1 (140 nL)				1 : 1 : 2 (140 nL)			
Solvent		1	2	3	4	5	6	7	8	9	10	11	12
MeOH	A	2	3	2	2	2	2	2	2	2	3	2	2
MeOH	B	2	2	2	2	2	2	2	2	2	3	2	3
DMF	C	1	1	1	1	1	1	1	1	1	1	1	1
DMF	D	1	1	1	1	1	1	1	1	1	1	1	1
$MeNO_2$	E	2	4	4[a]	3	3	4	4[a]	4	3	3	3	3
$MeNO_2$	F	2	2	3	3	3	2	2	4	3	3	3	3
1,4-Dioxane	G	2	2	4	4[a]	2	2	4	4[a]	2	4	4	4
1,4-Dioxane	H	2	3	4[a]	4	3	3	3	3	2	3	3	3

caffeine : quinol : 4-hydroxybenzoic acid (A : B : C) plate 2													
Volume of Oil		200 nL											
Volume of Stock Solution (A : B : C)		2 : 2 : 1 (140 nL)				2 : 1 : 2 (140 nL)				1 : 2 : 2 (140 nL)			
Solvent		1	2	3	4	5	6	7	8	9	10	11	12
MeOH	A	2	2	2	2	2	2	2	2	2	2	2	2
MeOH	B	2	2	2	2	2	2	2	2	2	2	2	2
DMF	C	1	1	1	1	1	1	1	1	1	1	1	1
DMF	D	1	1	1	1	1	1	1	1	1	1	1	1
$MeNO_2$	E	3	3	4	4	4	4[a]	4	3	3	4[a]	4	3
$MeNO_2$	F	2	2	3	3	3	3	3	3	3	3	4[a]	4
1,4-Dioxane	G	3	2	2	2	2	4	4[a]	4	2	2	4	4
1,4-Dioxane	H	2	4	4[a]	4	3	2	4[a]	3	2	2	4[a]	3

caffeine : quinol : 4-hydroxybenzoic acid (A : B : C) plate 3													
Volume of Oil		200 nL											
Volume of Stock Solution (A : B : C)		1 : 1 : 1 (140 nL)											
Solvent		1	2	3	4	5	6	7	8	9	10	11	12
MeOH	A	2	2	2	2	2	2	2	3	2	2	4[b]	2
MeOH	B	2	2	2	2	2	2	2	2	2	3	3	2
DMF	C	1	1	1	1	1	1	1	1	1	1	1	1
DMF	D	1	1	1	1	1	1	1	1	1	1	1	1
$MeNO_2$	E	3	3	4	4	4[a]	4	3	1	1	3	4[a]	4
$MeNO_2$	F	3	4[a]	4	4	4[a]	4	4	4	4[a]	4	4	4
1,4-Dioxane	G	2	3	3	3	3	3	2	1	3	4	4	3
1,4-Dioxane	H	2	4	4[a]	4	4	4	2	4	4[a]	3	4	4

SCXRD Unit Cell Analysis

[a] Binary co-crystal (caffeine: quinol, 1:1.5).

[b] Single-component crystal (quinol; CSD refcode: HYQUIN04).

caffeine: quinol: resorcinol

caffeine : quinol : resorcinol (A : B : C) plate 1													
Volume of Oil		200 nL											
Volume of Stock Solution (A : B : C)		2 : 1 : 1 (140 nL)				1 : 2 : 1 (140 nL)				1 : 1 : 2 (140 nL)			
Solvent		1	2	3	4	5	6	7	8	9	10	11	12
MeOH	A	4	3	4[a]	4	4	4[a]	4	3	4	4[a]	4	3
MeOH	B	3	4[a]	1	3	1	3	3	4[a]	3	3	3	3
DMF	C	3	2	2	2	3	4[b]	3	4	3	3	2	2
DMF	D	1	1	3	3	1	3	3	4[b]	3	4	3	2
$MeNO_2$	E	1	1	1	1	1	1	1	1	1	1	1	1
$MeNO_2$	F	1	1	1	1	1	1	1	1	1	1	1	1
1,4-Dioxane	G	3	4[b]	4	4	4	4	3	4[b]	4	3	3	4
1,4-Dioxane	H	3	3	4[a]	4[b]	3	3	3	3	4[a]	3	3	3

caffeine : quinol : resorcinol (A : B : C) plate 2													
Volume of Oil		200 nL											
Volume of Stock Solution (A : B : C)		2 : 2 : 1 (140 nL)				2 : 1 : 2 (140 nL)				1 : 2 : 2 (140 nL)			
Solvent		1	2	3	4	5	6	7	8	9	10	11	12
MeOH	A	4	4	4	4[a]	4	3	4	4[a]	4	3	3	3
MeOH	B	4[a]	4[a]	1	3	3	4	4[a]	4	3	3	1	3
DMF	C	1	1	1	3	3	1	3	4[b]	1	3	1	1
DMF	D	1	1	3	3	3	1	1	3	1	3	3	3
$MeNO_2$	E	1	1	1	1	1	1	1	1	1	1	1	1
$MeNO_2$	F	1	1	1	1	1	1	1	1	1	1	1	1
1,4-Dioxane	G	3	3	4	4[b]	4	4	3	3	4	3	4	3
1,4-Dioxane	H	3	4	4[a]	4[b]	3	3	4[a]	3	3	4	4[a]	3

caffeine : quinol : resorcinol (A : B : C) plate 3													
Volume of Oil		200 nL											
Volume of Stock Solution (A : B : C)		1 : 1 : 1 (140 nL)											
Solvent		1	2	3	4	5	6	7	8	9	10	11	12
MeOH	A	3	4	4	4[a]	4	4	4[a]	1	3	3	4[a]	4
MeOH	B	3	1	1	3	1	1	3	4[a]	3	3	3	3
DMF	C	1	1	1	3	1	1	1	3	1	3	3	3
DMF	D	1	4[b]	3	3	1	3	1	4[a]	3	3	4	4
$MeNO_2$	E	1	1	1	1	1	1	1	1	1	1	1	1
$MeNO_2$	F	1	1	1	1	1	1	1	1	1	1	1	1
1,4-Dioxane	G	4	4[b]	4	3	4	4	4[b]	1	3	3	3	3
1,4-Dioxane	H	4[b]	3	3	4[a]	3	3	4	3	4	3	4[b]	3

SCXRD Unit Cell Analysis

[a] Single-component crystal (quinol; CSD refcode: HYQUIN04).

[b] Binary co-crystal (caffeine: quinol, 1:1.5).

caffeine: quinol: tetramethylpyrazine

caffeine : quinol : tetramethylpyrazine (A : B : C) plate 1													
Volume of Oil		200 nL											
Volume of Stock Solution (A : B : C)		2 : 1 : 1 (140 nL)				1 : 2 : 1 (140 nL)				1 : 1 : 2 (140 nL)			
Solvent		1	2	3	4	5	6	7	8	9	10	11	12
MeOH	A	3	3	4[a]	4	3	3	3	3	3	3	3	3
MeOH	B	3	3	3	3	3	3	3	3	3	3	3	3
DMF	C	1	1	1	1	1	1	1	1	1	1	1	1
DMF	D	1	1	1	1	1	1	1	1	1	1	1	1
$MeNO_2$	E	3	3	4[a]	3	3	3	3	3	3	2	2	2
$MeNO_2$	F	3	3	3	3	3	3	3	3	3	3	3	3
1,4-Dioxane	G	3	3	3	3	3	3	3	3	3	3	3	3
1,4-Dioxane	H	3	4[b]	3	3	3	3	3	3	3	3	3	3

caffeine : quinol : tetramethylpyrazine (A : B : C) plate 2													
Volume of Oil		200 nL											
Volume of Stock Solution (A : B : C)		2 : 2 : 1 (140 nL)				2 : 1 : 2 (140 nL)				1 : 2 : 2 (140 nL)			
Solvent		1	2	3	4	5	6	7	8	9	10	11	12
MeOH	A	3	4[a]	3	4	3	3	3	3	3	3	3	3
MeOH	B	3	3	3	4[a]	4	4[a]	3	3	4*	3	3	3
DMF	C	1	1	1	1	1	1	1	1	1	1	1	1
DMF	D	1	1	1	1	1	1	1	1	1	1	1	1
$MeNO_2$	E	2	2	4[b]	4	4[b]	3	3	3	3	3	3	3
$MeNO_2$	F	3	3	3	3	3	3	3	3	3	3	3	3
1,4-Dioxane	G	3	3	3	3	3	3	3	3	3	3	3	3
1,4-Dioxane	H	3	3	3	3	3	3	4[b]	3	3	3	3	3

caffeine : quinol : tetramethylpyrazine (A : B : C) plate 3													
Volume of Oil		200 nL											
Volume of Stock Solution (A : B : C)		1 : 1 : 1 (140 nL)											
Solvent		1	2	3	4	5	6	7	8	9	10	11	12
MeOH	A	2	4[a]	3	4	3	3	3	3	3	3	3	3
MeOH	B	2	4	4[a]	3	4	3	2	3	3	3	3	3
DMF	C	2	1	1	1	1	1	2	1	1	1	1	1
DMF	D	2	1	1	1	1	1	2	1	1	1	1	1
$MeNO_2$	E	2	2	3	3	3	3	2	3	3	3	3	3
$MeNO_2$	F	3	3	3	3	3	3	3	3	3	3	3	3
1,4-Dioxane	G	2	3	3	3	3	3	2	3	3	3	3	3
1,4-Dioxane	H	2	3	3	3	3	3	2	3	3	3	3	4[b]

SCXRD Full Data Collection and Structure Refinement

* Binary co-crystal (quinol: tetramethylpyrazine, 0.5:0.5; CSD refcode: COZZOH) obtained from P2 B9 (MeOH, FC-40 oil).

SCXRD Unit Cell Analysis

[a] Single-component crystal (quinol; CSD refcode: HYQUIN04).

[b] Binary co-crystal (caffeine: quinol, 1:1.5).

caffeine: quinol: 1,4-dithiane-2,5-diol

caffeine : quinol : 1,4-dithiane-2,5-diol (A : B : C) plate 1													
Volume of Oil		200 nL											
Volume of Stock Solution (A : B : C)		2 : 1 : 1 (140 nL)				1 : 2 : 1 (140 nL)				1 : 1 : 2 (140 nL)			
Solvent		1	2	3	4	5	6	7	8	9	10	11	12
MeOH	A	3	3	3	3	3	3	3	3	4[a]	4	3	3
MeOH	B	3	3	3	3	3	3	3	3	3	3	3	3
DMF	C	3	3	3	3	3	3	3	3	3	3	3	3
DMF	D	3	3	3	3	3	3	3	3	3	3	3	3
$MeNO_2$	E	3	3	3	4[a]	4[a]	3	3	3	4[a]	4[a]	3	3
$MeNO_2$	F	3	3	3	3	3	3	3	3	3	3	3	3
1,4-Dioxane	G	3	3	3	3	3	3	3	3	3	3	3	3
1,4-Dioxane	H	3	4[b]	4[a]	4[b]	3	3	3	3	3	4[b]	4[a]	4

caffeine : quinol : 1,4-dithiane-2,5-diol (A : B : C) plate 2													
Volume of Oil		200 nL											
Volume of Stock Solution (A : B : C)		2 : 2 : 1 (140 nL)				2 : 1 : 2 (140 nL)				1 : 2 : 2 (140 nL)			
Solvent		1	2	3	4	5	6	7	8	9	10	11	12
MeOH	A	3	4[a]	4[a]	4	3	3	3	3	3	4[a]	3	3
MeOH	B	3	3	3	3	3	4[a]	3	4	3	3	3	3
DMF	C	3	3	3	3	3	3	3	3	3	3	3	3
DMF	D	3	3	3	3	3	3	3	3	3	3	3	3
$MeNO_2$	E	3	3	4[a]	3	3	4[a]	4	3	3	3	4[a]	3
$MeNO_2$	F	3	3	3	3	3	3	3	3	3	3	3	3
1,4-Dioxane	G	3	3	3	3	3	3	3	3	3	3	3	3
1,4-Dioxane	H	3	4[b]	3	4[b]	3	3	3	3	4[a]	3	3	3

caffeine : quinol : 1,4-dithiane-2,5-diol (A : B : C) plate 3													
Volume of Oil		200 nL											
Volume of Stock Solution (A : B : C)		1 : 1 : 1 (140 nL)											
Solvent		1	2	3	4	5	6	7	8	9	10	11	12
MeOH	A	2	3	3	3	3	3	2	4[a]	3	3	3	3
MeOH	B	2	3	3	3	3	3	2	3	3	3	3	3
DMF	C	3	3	3	3	3	3	3	3	3	3	3	3
DMF	D	3	3	3	3	3	3	3	3	3	3	3	3
$MeNO_2$	E	3	3	4[a]	4	4	4	3	3	3	3	3	3
$MeNO_2$	F	3	3	3	3	3	3	3	3	3	3	3	3
1,4-Dioxane	G	2	3	3	3	3	3	2	3	3	3	3	3
1,4-Dioxane	H	2	3	3	4[a]	3	3	2	3	4[b]	4	4	3

SCXRD Unit Cell Analysis

[a] Single-component crystal (quinol; CSD refcode: HYQUIN04).

[b] Binary co-crystal (caffeine: quinol, 1:1.5).

caffeine: quinol: 2,3,5-tetramethylbenzene-1,4-diol

caffeine : quinol : 2,3,5-tetramethylbenzene-1,4-diol (A : B : C) plate 1													
**Volume of Oil**		**200 nL**											
**Volume of Stock Solution (A : B : C)**		**2 : 1 : 1 (140 nL)**				**1 : 2 : 1 (140 nL)**				**1 : 1 : 2 (140 nL)**			
**Solvent**		**1**	**2**	**3**	**4**	**5**	**6**	**7**	**8**	**9**	**10**	**11**	**12**
MeOH	**A**	3	3	3	3	3	3	4[a]	3	3	4	4[a]	4
MeOH	**B**	3	3	3	3	3	4	4	4[a]	3	3	3	3
DMF	**C**	1	1	1	1	1	1	1	1	1	1	1	1
DMF	**D**	1	1	1	1	1	1	1	1	1	1	1	1
$MeNO_2$	**E**	3	3	3	3	3	3	3	3	3	3	3	3
$MeNO_2$	**F**	3	3	3	3	3	3	3	3	3	3	3	3
1,4-Dioxane	**G**	3	3	3	3	3	3	3	3	3	3	3	3
1,4-Dioxane	**H**	3	3	3	3	3	3	3	3	4[a]	3	3	3

caffeine : quinol : 2,3,5-tetramethylbenzene-1,4-diol (A : B : C) plate 2													
**Volume of Oil**		**200 nL**											
**Volume of Stock Solution (A : B : C)**		**2 : 2 : 1 (140 nL)**				**2 : 1 : 2 (140 nL)**				**1 : 2 : 2 (140 nL)**			
**Solvent**		**1**	**2**	**3**	**4**	**5**	**6**	**7**	**8**	**9**	**10**	**11**	**12**
MeOH	**A**	3	3	4	4[a]	4[a]	3	3	3	3	4[a]	3	3
MeOH	**B**	3	3	3	3	3	3	3	3	4[a]	3	3	3
DMF	**C**	1	1	1	1	1	1	1	1	1	1	1	1
DMF	**D**	1	1	1	1	1	1	1	1	1	1	1	1
$MeNO_2$	**E**	3	3	3	3	3	3	3	3	3	3	3	3
$MeNO_2$	**F**	3	3	3	3	3	3	3	3	3	3	3	3
1,4-Dioxane	**G**	3	3	3	3	3	3	3	3	3	3	3	3
1,4-Dioxane	**H**	3	4[a]	4[b]	4	3	3	3	3	3	3	3	3

caffeine : quinol : 2,3,5-tetramethylbenzene-1,4-diol (A : B : C) plate 3													
**Volume of Oil**		**200 nL**											
**Volume of Stock Solution (A : B : C)**		**1 : 1 : 1 (140 nL)**											
**Solvent**		**1**	**2**	**3**	**4**	**5**	**6**	**7**	**8**	**9**	**10**	**11**	**12**
MeOH	**A**	2	3	4[a]	3	4	4	2	3	4	4[a]	4	3
MeOH	**B**	2	3	3	3	3	4[a]	2	3	3	3	3	3
DMF	**C**	1	1	1	1	1	1	1	1	1	1	1	1
DMF	**D**	1	1	1	1	1	1	1	1	1	1	1	1
$MeNO_2$	**E**	3	3	3	3	3	3	2	3	3	3	3	3
$MeNO_2$	**F**	2	3	3	4[a]	3	4	2	3	3	4[a]	3	4
1,4-Dioxane	**G**	3	3	3	3	3	3	2	4	4	4	4[b]	4[b]
1,4-Dioxane	**H**	3	3	4	4[a]	4	4	2	4[a]	4	4	3	3

SCXRD Unit Cell Analysis

[a] Single-component crystal (quinol; CSD refcode: HYQUIN04).

[b] Binary co-crystal (caffeine: quinol, 1:1.5).

4,4’-bipyridine: glutaric acid: tetramethylpyrazine

4,4'-bipyridine : glutaric acid : tetramethylpyrazine (A : B : C) plate 1													
**Volume of Oil**		**200 nL**											
**Volume of Stock Solution (A : B : C)**		**2 : 1 : 1 (140 nL)**				**1 : 2 : 1 (140 nL)**				**1 : 1 : 2 (140 nL)**			
**Solvent**		**1**	**2**	**3**	**4**	**5**	**6**	**7**	**8**	**9**	**10**	**11**	**12**
MeOH	**A**	3	3	4^a	4^a	3	3	4	4^a	4	3	4^a	4
MeOH	**B**	3	3	3	3	3	3	3	3	3	3	3	3
DMF	**C**	3	3	3	3	3	3	3	4^a	3	4^a	3	3
DMF	**D**	3	3	3	3	3	3	4	4	3	3	3	4
$MeNO_2$	**E**	4	4	4^a	4^a	3	3	4^a	4	3	3	4	4^a
$MeNO_2$	**F**	3	3	3	3	4^a	3	3	3	4	3	4^a	3
1,4-Dioxane	**G**	3	3	4^a	3	4	3	4^a	3	3	3	4^a	3
1,4-Dioxane	**H**	3	3	3	3	3	3	3	3	3	3	3	3

4,4'-bipyridine : glutaric acid : tetramethylpyrazine (A : B : C) plate 2													
**Volume of Oil**		**200 nL**											
**Volume of Stock Solution (A : B : C)**		**2 : 2 : 1 (140 nL)**				**2 : 1 : 2 (140 nL)**				**1 : 2 : 2 (140 nL)**			
**Solvent**		**1**	**2**	**3**	**4**	**5**	**6**	**7**	**8**	**9**	**10**	**11**	**12**
MeOH	**A**	3	3	4^a	4^a	4^a	4^a	3	4	3	4	4	4
MeOH	**B**	3	3	3	3	3	3	4^a	4	3	3	4	4
DMF	**C**	3	3	3	3	3	3	3	4	3	3	3	4^a
DMF	**D**	3	3	3	3	3	3	3	3	3	3	3	3
$MeNO_2$	**E**	4^a	4	4	4	4^a	4^a	4	4	4^a	4	4	4^a
$MeNO_2$	**F**	4	4	3	4^a	4	4	4^a	4^a	4	4	4^a	4
1,4-Dioxane	**G**	3	3	4^a	3	4^a	3	4	3	3	3	4^a	4
1,4-Dioxane	**H**	3	3	3	3	3	3	3	3	3	3	3	3

4,4'-bipyridine : glutaric acid : tetramethylpyrazine (A : B : C) plate 3													
**Volume of Oil**		**200 nL**											
**Volume of Stock Solution (A : B : C)**		**1 : 1 : 1 (140 nL)**											
**Solvent**		**1**	**2**	**3**	**4**	**5**	**6**	**7**	**8**	**9**	**10**	**11**	**12**
MeOH	**A**	3	3	4^a	4	3	3	3	4^a	4	4	4	4
MeOH	**B**	3	4^a	3	3	3	3	3	3	3	3	3	4^a
DMF	**C**	3	3	4^a	3	3	3	3	4	4^a	4	4	4
DMF	**D**	3	3	3	3	3	3	3	3	3	4	3	3
$MeNO_2$	**E**	4^a	3	4^a	4	4	4	3	4	4	4^a	4	4
$MeNO_2$	**F**	4^a	3	3	3	3	3	3	4	4^a	4	4	4
1,4-Dioxane	**G**	3	3	3	3	3	3	3	4	4	4^a	4^a	4
1,4-Dioxane	**H**	3	3	3	3	3	3	3	3	3	3	3	3

SCXRD Unit Cell Analysis

[a] Binary co-crystal (4,4’-bipyridine: glutaric acid, 2:2; CSD refcode: SOVDIQ).

4,4’-bipyridine: glutaric acid: phenazine

4,4'-bipyridine : glutaric acid : phenazine (A : B : C) plate 1													
Volume of Oil		200 nL											
Volume of Stock Solution (A : B : C)		2 : 1 : 1 (140 nL)				1 : 2 : 1 (140 nL)				1 : 1 : 2 (140 nL)			
Solvent		1	2	3	4	5	6	7	8	9	10	11	12
MeOH	A	3	3	3	3	3	3	3	3	3	3	3	3
MeOH	B	3	3	3	3	3	3	3	3	3	3	3	3
DMF	C	3	3	3	3	4[a]	3	3	4[a]	3	3	3	3
DMF	D	4	4[a]	4[a]	3	3	3	3	3	4	3	3	3
$MeNO_2$	E	3	3	4[a]	3	3	3	3	3	3	3	3	4[a]
$MeNO_2$	F	3	3	4[a]	3	4[a]	4[a]	4	3	4[a]	4[a]	4	4
1,4-Dioxane	G	4	4[a]	4[a]	3	4	4[a]	4[a]	4	4[a]	4[a]	3	3
1,4-Dioxane	H	3	3	3	3	3	3	3	3	3	4[a]	3	3

4,4'-bipyridine : glutaric acid : phenazine (A : B : C) plate 2													
Volume of Oil		200 nL											
Volume of Stock Solution (A : B : C)		2 : 2 : 1 (140 nL)				2 : 1 : 2 (140 nL)				1 : 2 : 2 (140 nL)			
Solvent		1	2	3	4	5	6	7	8	9	10	11	12
MeOH	A	3	3	3	3	3	3	3	3	3	3	3	3
MeOH	B	3	3	3	3	3	3	3	3	3	3	3	3
DMF	C	3	3	3	3	3	3	3	4[a]	3	3	3	4[a]
DMF	D	3	3	3	3	3	3	3	3	3	3	3	3
$MeNO_2$	E	3	4[a]	3	3	3	4[a]	3	3	3	3	3	3
$MeNO_2$	F	4[a]	4	4[a]	4[a]	4	4	4[a]	4[a]	4	4[a]	4[a]	4[a]
1,4-Dioxane	G	3	3	4[a]	4[a]	4[a]	4[a]	4[a]	4[a]	4[a]	3	4[a]	4[a]
1,4-Dioxane	H	3	3	3	3	3	3	3	3	3	3	3	3

4,4'-bipyridine : glutaric acid : phenazine (A : B : C) plate 3													
Volume of Oil		200 nL											
Volume of Stock Solution (A : B : C)		1 : 1 : 1 (140 nL)											
Solvent		1	2	3	4	5	6	7	8	9	10	11	12
MeOH	A	2	3	3	3	3	3	2	3	3	3	3	3
MeOH	B	2	3	3	3	3	3	2	3	3	3	3	4[a]
DMF	C	3	3	3	4[a]	3	3	3	4	4	4	4	4[a]
DMF	D	2	4[a]	3	4	3	3	3	4[a]	3	3	3	3
$MeNO_2$	E	3	3	3	3	4[a]	3	3	4[a]	4	4	3	4
$MeNO_2$	F	3	4	4[a]	4	4	3	2	4	4	4	4	4
1,4-Dioxane	G	3	4	4	4	4[a]	4	3	4[a]	4	3	3	3
1,4-Dioxane	H	3	4	4[a]	4	4	4	3	3	4[a]	3	3	3

SCXRD Unit Cell Analysis

[a] Binary co-crystal (4,4’-bipyridine: glutaric acid, 2:2; CSD refcode: SOVDIQ).

4,4'-bipyridine: glutaric acid: 3,3'-thiodipropionic acid

4,4'-bipyridine : glutaric acid : 3,3'-thiodipropionic acid (A : B : C) plate 1													
**Volume of Oil**		**200 nL**											
**Volume of Stock Solution (A : B : C)**		**2 : 1 : 1 (140 nL)**				**1 : 2 : 1 (140 nL)**				**1 : 1 : 2 (140 nL)**			
**Solvent**		**1**	**2**	**3**	**4**	**5**	**6**	**7**	**8**	**9**	**10**	**11**	**12**
MeOH	**A**	3	2	2	2	2	2	2	2	2	2	2	2
MeOH	**B**	3	3	4^{b}	3	3	3	3	3	3	3	3	3
DMF	**C**	3	4^{b}	4^{b}	4	4	4	4^{b}	4^{b}	4^{b}	4^{b}	4	4
DMF	**D**	3	4^{b}	3	3	3	4^{b}	3	3	3	3	3	3
$MeNO_2$	**E**	3	3	4^{b}	3	3	3	3	3	3	3	3	4^{b}
$MeNO_2$	**F**	3	3	4^{b}	3	4^{b}	4^{b}	4	4	4^{b}	4^{b}	4	4
1,4-Dioxane	**G**	3	4^{b}	3	3	3	4^{b}	3	3	3	4^{b}	3	3
1,4-Dioxane	**H**	3	3	3	3	3	3	3	3	4^{b}	3	3	3

4,4'-bipyridine : glutaric acid : 3,3'-thiodipropionic acid (A : B : C) plate 2													
**Volume of Oil**		**200 nL**											
**Volume of Stock Solution (A : B : C)**		**2 : 2 : 1 (140 nL)**				**2 : 1 : 2 (140 nL)**				**1 : 2 : 2 (140 nL)**			
**Solvent**		**1**	**2**	**3**	**4**	**5**	**6**	**7**	**8**	**9**	**10**	**11**	**12**
MeOH	**A**	2	3	2	2	2	2	2	2	2	2	2	2
MeOH	**B**	4^{b}	3	3	3	3	3	3	3	3	3	3	4^{b}
DMF	**C**	3	3	4^{b}	4^{b}	3	4	4^{b}	4^{b}	4^{b}	4^{b}	4	4
DMF	**D**	3	4	3	4	3	3	3	3	3	4	3	3
$MeNO_2$	**E**	3	4^{b}	3	3	3	4^{b}	3	3	3	3	3	3
$MeNO_2$	**F**	4^{b}	4^{b}	3	4^{b}	4	4^{b}	4^{b}	4	4	4^{b}	4^{b}	4
1,4-Dioxane	**G**	3	3	3	4^{b}	3	4^{b}	3	4	3	3	4^{b}	4^{b}
1,4-Dioxane	**H**	3	3	4^{b}	3	4^{b}	3	3	3	3	3	3	3

4,4'-bipyridine : glutaric acid : 3,3'-thiodipropionic acid (A : B : C) plate 3													
**Volume of Oil**		**200 nL**											
**Volume of Stock Solution (A : B : C)**		**1 : 1 : 1 (140 nL)**											
**Solvent**		**1**	**2**	**3**	**4**	**5**	**6**	**7**	**8**	**9**	**10**	**11**	**12**
MeOH	**A**	2	2	2	2	2	2	2	3	4^{a}	4	4	4
MeOH	**B**	2	3	3	3	3	3	3	3	3	3	3	3
DMF	**C**	3	4	4	4	4*	4	3	3	3	3	3	3
DMF	**D**	3	4	4^{b}	4	4	4	4^{b}	4	4	4^{b}	4	4
$MeNO_2$	**E**	2	3	3	3	3	3	2	3	3	3	3	3
$MeNO_2$	**F**	2	3	3	3	3	3	2	3	3	3	3	3
1,4-Dioxane	**G**	2	3	3	3	3	3	3	4	4	4	4^{b}	4
1,4-Dioxane	**H**	3	4	4	4^{b}	4	3	3	3	3	3	3	3

SCXRD Full Data Collection and Structure Refinement

* Binary co-crystal (4,4'-bipyridine: 3,3'-thiodipropionic acid, 0.5:0.5; CSD refcode: SOVHEQ), from well P3 C5 (DMF, PDMSO oil).

SCXRD Unit Cell Analysis

[a] Single-component crystal (glutaric acid; CSD refcode: GLURAC02).

[b] Binary crystal (4,4'-bipyridine: 3,3'-thiodipropionic acid, 0.5:0.5; CSD refcode: SOVHEQ).

4,4’-bipyridine: glutaric acid: trimesic acid

4,4'-bipyridine : glutaric acid : trimesic acid (A : B : C) plate 1													
**Volume of Oil**		**200 nL**											
**Volume of Stock Solution (A : B : C)**		**2 : 1 : 1 (140 nL)**				**1 : 2 : 1 (140 nL)**				**1 : 1 : 2 (140 nL)**			
**Solvent**		**1**	**2**	**3**	**4**	**5**	**6**	**7**	**8**	**9**	**10**	**11**	**12**
MeOH	**A**	3	3	3	3	3	3	3	3	3	3	3	3
MeOH	**B**	3	3	3	3	3	3	3	3	3	3	3	3
DMF	**C**	3	4[a]	4[a]	4	4[a]	3	3	4[a]	3	4[a]	4[a]	4
DMF	**D**	4[a]	4[a]	4	4	4	4	4[a]	4[a]	4[a]	4[a]	4	4
$MeNO_2$	**E**	3	3	3	4[a]	3	3	3	3	3	4[a]	3	3
$MeNO_2$	**F**	3	3	3	3	3	3	3	3	3	3	3	3
1,4-Dioxane	**G**	3	3	3	3	3	3	3	3	3	3	3	3
1,4-Dioxane	**H**	3	3	3	3	3	3	3	3	3	3	3	3

4,4'-bipyridine : glutaric acid : trimesic acid (A : B : C) plate 2													
**Volume of Oil**		**200 nL**											
**Volume of Stock Solution (A : B : C)**		**2 : 2 : 1 (140 nL)**				**2 : 1 : 2 (140 nL)**				**1 : 2 : 2 (140 nL)**			
**Solvent**		**1**	**2**	**3**	**4**	**5**	**6**	**7**	**8**	**9**	**10**	**11**	**12**
MeOH	**A**	3	3	3	3	3	3	3	3	3	3	3	3
MeOH	**B**	3	3	3	3	3	3	3	3	3	3	3	3
DMF	**C**	4	4[a]	4[a]	4	3	3	4[a]	4[a]	4	4	4[a]	4[a]
DMF	**D**	4[a]	3	3	4[a]	4[a]	4[a]	4	4	4[a]	3	4	4[a]
$MeNO_2$	**E**	3	3	3	3	3	4[a]	3	3	3	3	3	3
$MeNO_2$	**F**	3	3	3	3	3	3	3	3	3	4[a]	3	4
1,4-Dioxane	**G**	3	3	3	3	3	3	3	3	3	3	3	3
1,4-Dioxane	**H**	3	3	3	3	3	3	3	3	3	3	3	3

4,4'-bipyridine : glutaric acid : trimesic acid (A : B : C) plate 3													
**Volume of Oil**		**200 nL**											
**Volume of Stock Solution (A : B : C)**		**1 : 1 : 1 (140 nL)**											
**Solvent**		**1**	**2**	**3**	**4**	**5**	**6**	**7**	**8**	**9**	**10**	**11**	**12**
MeOH	**A**	2	3	3	3	3	3	2	3	3	3	3	3
MeOH	**B**	2	3	3	3	3	3	2	3	3	3	3	3
DMF	**C**	4[a]	3	4	4[a]	3	3	4[a]	4[a]	4	3	3	3
DMF	**D**	3	3	3	3	4[a]	4	4	4[a]	3	3	3	3
$MeNO_2$	**E**	3	3	3	3	3	3	3	3	3	3	3	3
$MeNO_2$	**F**	3	3	3	3	4	4[a]	3	4[a]	3	4	3	3
1,4-Dioxane	**G**	3	3	3	3	3	3	3	3	3	3	3	3
1,4-Dioxane	**H**	3	3	3	3	3	3	3	3	3	3	3	3

SCXRD Unit Cell Analysis

[a] Binary co-crystal (4,4’-bipyridine: glutaric acid, 2:2; CSD refcode: SOVDIQ).

4,4'-bipyridine: glutaric acid: 5-nitroisophthalic acid

4,4'-bipyridine : glutaric acid : 5-nitroisophthalic acid (A : B : C) plate 1													
Volume of Oil		200 nL											
Volume of Stock Solution (A : B : C)		2 : 1 : 1 (140 nL)				1 : 2 : 1 (140 nL)				1 : 1 : 2 (140 nL)			
Solvent		1	2	3	4	5	6	7	8	9	10	11	12
MeOH	A	3	3	3	4[a]	3	3	3	4[a]	3	3	3	4[a]
MeOH	B	3	3	4[a]	3	3	3	3	3	3	3	3	3
DMF	C	4[a]	4[a]	4	4	4	4	4[a]	4[a]	4	4[a]	4[a]	4
DMF	D	3	3	3	3	3	3	3	3	3	3	3	3
$MeNO_2$	E	3	4[a]	3	3	3	3	3	4[a]	3	3	4[a]	3
$MeNO_2$	F	4	4[a]	4[a]	4	4[a]	4[a]	3	3	4[a]	4[a]	4	4
1,4-Dioxane	G	3	3	3	3	3	3	3	3	3	3	4[a]	3
1,4-Dioxane	H	3	3	3	3	3	3	3	3	3	3	3	3

4,4'-bipyridine : glutaric acid : 5-nitroisophthalic acid (A : B : C) plate 2													
Volume of Oil		200 nL											
Volume of Stock Solution (A : B : C)		2 : 2 : 1 (140 nL)				2 : 1 : 2 (140 nL)				1 : 2 : 2 (140 nL)			
Solvent		1	2	3	4	5	6	7	8	9	10	11	12
MeOH	A	3	4[a]	3	3	3	3	3	4[a]	3	4[a]	3	3
MeOH	B	4	3	4[a]	3	4[a]	3	3	3	4[a]	3	3	3
DMF	C	4	4[a]	4[a]	4	4	3	4[a]	3	4	4	4[a]	4[a]
DMF	D	3	3	3	3	3	3	3	3	3	3	3	3
$MeNO_2$	E	3	4[a]	3	4	3	3	3	4[a]	3	4[a]	3	3
$MeNO_2$	F	4[a]	4[a]	4	4	4[a]	4	4[a]	4	4[a]	4[a]	4	4
1,4-Dioxane	G	3	3	3	3	3	3	3	3	3	3	3	3
1,4-Dioxane	H	3	3	3	3	3	3	3	3	3	3	4[a]	3

4,4'-bipyridine : glutaric acid : 5-nitroisophthalic acid (A : B : C) plate 3													
Volume of Oil		200 nL											
Volume of Stock Solution (A : B : C)		1 : 1 : 1 (140 nL)											
Solvent		1	2	3	4	5	6	7	8	9	10	11	12
MeOH	A	3	3	3	3	3	3	3	4	4	3	4[a]	4
MeOH	B	3	4	3	3	4[a]	4	3	3	3	3	3	3
DMF	C	3	4[a]	3	4	4	3	4[a]	3	3	3	3	4[a]
DMF	D	3	3	3	4[a]	3	3	3	3	3	3	3	3
$MeNO_2$	E	3	3	3	3	3	4[a]	3	3	4	4	4[a]	4
$MeNO_2$	F	3	4	4[a]	4	4	4	4[a]	4[a]	4[a]	4	4	4
1,4-Dioxane	G	3	3	3	3	3	3	3	4[a]	3	3	3	4[a]
1,4-Dioxane	H	3	3	3	3	3	3	3	3	3	3	3	3

SCXRD Unit Cell Analysis

[a] Binary co-crystal (4,4'-bipyridine: glutaric acid, 2:2; CSD refcode: SOVDIQ).

4,4’-bipyridine: 3-hydroxy-2-naphthoic acid: tetramethylpyrazine

4,4'-bipyridine : 3-hydroxy-2-naphthoic acid : tetramethylpyrazine (A : B : C) plate 1													
Volume of Oil		200 nL											
Volume of Stock Solution (A : B : C)		2 : 1 : 1 (140 nL)				1 : 2 : 1 (140 nL)				1 : 1 : 2 (140 nL)			
Solvent		1	2	3	4	5	6	7	8	9	10	11	12
MeOH	A	4[a]	3	3	3	3	3	3	3	3	3	3	3
MeOH	B	3	3	3	3	3	3	3	3	3	3	3	3
DMF	C	4[a]	3	4[a]	4[a]	4	4[a]	4[a]	4	4	4[a]	4[a]	4
DMF	D	4	4	4[a]	4	4	4	4	4[a]	4	4	4[a]	4
$MeNO_2$	E	3	4[b]	4[b]	4	3	4[b]	4[b]	3	3	3	3	3
$MeNO_2$	F	2	2	3	3	3	3	3	3	2	2	3	3
1,4-Dioxane	G	2	2	4[a]	3	2	2	2	2	2	2	3	4[a]
1,4-Dioxane	H	2	2	2	2	2	4[a]	2	2	3	4[a]	3	3

4,4'-bipyridine : 3-hydroxy-2-naphthoic acid : tetramethylpyrazine (A : B : C) plate 2													
Volume of Oil		200 nL											
Volume of Stock Solution (A : B : C)		2 : 2 : 1 (140 nL)				2 : 1 : 2 (140 nL)				1 : 2 : 2 (140 nL)			
Solvent		1	2	3	4	5	6	7	8	9	10	11	12
MeOH	A	3	3	3	3	3	3	3	3	3	3	3	3
MeOH	B	3	3	3	3	3	3	3	3	3	3	3	3
DMF	C	3	3	4[a]	4[a]	3	4	4[a]	4	4[a]	4	4	4[a]
DMF	D	4	4[a]	4	4	3	4[a]	4[a]	4	3	3	4[a]	4[a]
$MeNO_2$	E	4[b]	4	3	3	4[b]	3	4	3	4	4[b]	4	4
$MeNO_2$	F	3	3	4[b]	4[b]	3	3	3	3	3	4[b]	4	3
1,4-Dioxane	G	2	2	4[a]	4[a]	2	2	2	2	2	2	2	2
1,4-Dioxane	H	2	2	2	2	2	2	2	2	2	4	3	4[a]

4,4'-bipyridine : 3-hydroxy-2-naphthoic acid : tetramethylpyrazine (A : B : C) plate 3													
Volume of Oil		200 nL											
Volume of Stock Solution (A : B : C)		1 : 1 : 1 (140 nL)											
Solvent		1	2	3	4	5	6	7	8	9	10	11	12
MeOH	A	2	3	3	3	3	3	2	3	3	3	3	3
MeOH	B	2	3	3	3	3	3	2	3	3	3	3	3
DMF	C	4[a]	4[a]	4	4	4[a]	4	4	4	4	4[a]	4	4
DMF	D	4[a]	4	4	4	4	4	4[a]	4	4[a]	4	4	4[a]
$MeNO_2$	E	4	4	4	4[b]	4	4	4	4[b]	4	3	3	3
$MeNO_2$	F	2	3	3	3	3	3	3	3	3	3	3	3
1,4-Dioxane	G	3	2	2	2	2	2	3	4[a]	4	4	3	3
1,4-Dioxane	H	3	3	3	3	3	4[a]	3	2	2	2	2	3

SCXRD Unit Cell Analysis

[a] Binary co-crystal (4,4’-bipyridine: 3-hydroxy-2-naphthoic acid, 0.5:1; CSD refcode: GEHROB).

[b] Binary co-crystal (4,4’-bipyridine: 3-hydroxy-2-naphthoic acid, 1.5:1).

4,4’-bipyridine: 3-hydroxy-2-naphthoic acid: phenazine

4,4'-bipyridine : 3-hydroxy-2-naphthoic acid : phenazine (A : B : C) plate 1													
Volume of Oil		200 nL											
Volume of Stock Solution (A : B : C)		2 : 1 : 1 (140 nL)				1 : 2 : 1 (140 nL)				1 : 1 : 2 (140 nL)			
Solvent		1	2	3	4	5	6	7	8	9	10	11	12
MeOH	A	3	3	3	3	3	3	3	3	3	3	3	3
MeOH	B	3	3	3	3	3	3	3	3	3	3	3	3
DMF	C	4	4[a]	4[a]	4	4[a]	4[a]	4	4	4[a]	4	4[a]	4
DMF	D	4	4	4[a]	4[a]	4	4	4[a]	4[a]	4	4[a]	4[a]	4
$MeNO_2$	E	3	3	3	3	3	3	3	3	3	3	3	3
$MeNO_2$	F	3	3	3	3	3	3	3	3	3	3	3	3
1,4-Dioxane	G	3	3	3	4[a]	3	4	3	4[a]	3	3	4[a]	4[a]
1,4-Dioxane	H	3	3	3	3	3	3	3	3	3	3	3	3

4,4'-bipyridine : 3-hydroxy-2-naphthoic acid : phenazine (A : B : C) plate 2													
Volume of Oil		200 nL											
Volume of Stock Solution (A : B : C)		2 : 2 : 1 (140 nL)				2 : 1 : 2 (140 nL)				1 : 2 : 2 (140 nL)			
Solvent		1	2	3	4	5	6	7	8	9	10	11	12
MeOH	A	3	3	3	3	4[a]	3	4[a]	3	3	3	3	3
MeOH	B	3	3	3	3	3	3	3	3	3	3	3	3
DMF	C	4[a]	4[a]	4	4	4[a]	4[a]	4	4	4[a]	4[a]	4	4
DMF	D	4[a]	4	4	4[a]	4	4	4[a]	4[a]	4	4[a]	4[a]	4
$MeNO_2$	E	3	3	3	3	3	3	3	3	3	3	3	3
$MeNO_2$	F	3	3	3	3	3	3	3	3	3	3	3	3
1,4-Dioxane	G	3	4[a]	3	4[a]	3	3	3	3	3	3	3	4[a]
1,4-Dioxane	H	3	3	3	3	3	3	3	3	3	3	3	3

4,4'-bipyridine : 3-hydroxy-2-naphthoic acid : phenazine (A : B : C) plate 3													
Volume of Oil		200 nL											
Volume of Stock Solution (A : B : C)		1 : 1 : 1 (140 nL)											
Solvent		1	2	3	4	5	6	7	8	9	10	11	12
MeOH	A	4	4	4[a]	4[a]	4[a]	3	3	3	3	3	3	3
MeOH	B	3	3	3	3	3	3	3	3	3	3	3	3
DMF	C	4[a]	4	4[a]	4	4	4	4	4[a]	4	4	4	4
DMF	D	4[a]	4	4	4	4[a]	4	4	4	4	4	4[a]	4
$MeNO_2$	E	3	3	3	3	3	3	3	3	3	3	3	3
$MeNO_2$	F	3	3	3	3	3	3	3	3	3	3	3	3
1,4-Dioxane	G	3	3	3	3	3	3	3	4	3	4[a]	4	4
1,4-Dioxane	H	3	3	3	3	3	3	3	3	3	3	3	3

SCXRD Unit Cell Analysis

[a] Binary co-crystal (4,4’-bipyridine: 3-hydroxy-2-naphthoic acid, 0.5:1; CSD refcode: GEHROB).

4,4’-bipyridine: 3-hydroxy-2-naphthoic acid: 2,2’-bipyridine

4,4'-bipyridine : 3-hydroxy-2-naphthoic acid : 2,2'-bipyridine (A : B : C) plate 1													
**Volume of Oil**		**200 nL**											
**Volume of Stock Solution (A : B : C)**		**2 : 1 : 1 (140 nL)**				**1 : 2 : 1 (140 nL)**				**1 : 1 : 2 (140 nL)**			
**Solvent**		**1**	**2**	**3**	**4**	**5**	**6**	**7**	**8**	**9**	**10**	**11**	**12**
MeOH	**A**	3	3	3	3	3	3	3	3	3	3	3	3
MeOH	**B**	3	3	3	3	3	3	3	3	3	3	3	3
DMF	**C**	3	3	3	3	3	3	3	3	3	3	3	3
DMF	**D**	3	4[a]	3	3	3	3	3	3	3	3	3	3
$MeNO_2$	**E**	3	3	3	3	3	3	3	3	3	3	3	3
$MeNO_2$	**F**	2	3	2	3	3	3	2	3	3	3	3	3
1,4-Dioxane	**G**	3	3	4[a]	3	3	3	3	3	4[a]	3	3	3
1,4-Dioxane	**H**	3	3	3	3	3	3	3	3	3	3	3	3

4,4'-bipyridine : 3-hydroxy-2-naphthoic acid : 2,2'-bipyridine (A : B : C) plate 2													
**Volume of Oil**		**200 nL**											
**Volume of Stock Solution (A : B : C)**		**2 : 2 : 1 (140 nL)**				**2 : 1 : 2 (140 nL)**				**1 : 2 : 2 (140 nL)**			
**Solvent**		**1**	**2**	**3**	**4**	**5**	**6**	**7**	**8**	**9**	**10**	**11**	**12**
MeOH	**A**	3	3	3	3	3	3	3	3	3	3	3	3
MeOH	**B**	3	3	4[a]	3	3	3	3	3	3	3	3	3
DMF	**C**	3	3	3	3	3	3	3	3	3	3	3	3
DMF	**D**	3	3	3	3	3	3	3	3	3	3	3	3
$MeNO_2$	**E**	4[b]	3	3	3	3	3	3	3	3	3	3	3
$MeNO_2$	**F**	3	3	3	3	2	3	3	3	2	3	2	3
1,4-Dioxane	**G**	3	2	3	3	3	3	3	3	3	3	3	3
1,4-Dioxane	**H**	3	3	3	3	3	3	3	3	3	3	3	3

4,4'-bipyridine : 3-hydroxy-2-naphthoic acid : 2,2'-bipyridine (A : B : C) plate 3													
**Volume of Oil**		**200 nL**											
**Volume of Stock Solution (A : B : C)**		**1 : 1 : 1 (140 nL)**											
**Solvent**		**1**	**2**	**3**	**4**	**5**	**6**	**7**	**8**	**9**	**10**	**11**	**12**
MeOH	**A**	2	3	3	3	3	3	3	3	3	3	3	4[a]
MeOH	**B**	3	4[a]	3	3	4[a]	3	3	3	3	3	3	3
DMF	**C**	3	3	4[a]	3	3	3	3	3	3	3	3	3
DMF	**D**	3	3	3	3	3	3	3	4[a]	3	3	3	3
$MeNO_2$	**E**	2	3	3	3	3	4[b]	2	2	2	2	2	2
$MeNO_2$	**F**	2	3	3	3	3	3	2	3	3	3	3	3
1,4-Dioxane	**G**	2	3	3	3	3	3	2	4[a]	3	3	4[a]	4
1,4-Dioxane	**H**	3	2	2	2	3	3	2	3	3	3	3	3

SCXRD Unit Cell Analysis

[a] Binary co-crystal (4,4’-bipyridine: 3-hydroxy-2-naphthoic acid, 0.5:1; CSD refcode: GEHROB).

[b] Binary co-crystal (4,4’-bipyridine: 3-hydroxy-2-naphthoic acid, 1.5:1).

4,4’-bipyridine: 3-hydroxy-2-naphthoic acid: 1,2-bis(4-pyridyl)ethane

4,4'-bipyridine : 3-hydroxy-2-naphthoic acid : 1,2-bis(4-pyridyl)ethane (A : B : C) plate 1													
Volume of Oil		200 nL											
Volume of Stock Solution (A : B : C)		2 : 1 : 1 (140 nL)				1 : 2 : 1 (140 nL)				1 : 1 : 2 (140 nL)			
Solvent		1	2	3	4	5	6	7	8	9	10	11	12
MeOH	A	3	3	3	3	3	3	3	3	3	3	3	3
MeOH	B	3	3	3	3	3	3	3	3	3	3	3	3
DMF	C	3	4[a]	3	3	3	3	3	3	3	3	3	3
DMF	D	3	4[a]	3	3	3	3	3	3	3	3	3	3
$MeNO_2$	E	3	3	3	3	3	3	3	3	3	3	3	3
$MeNO_2$	F	3	3	3	3	3	3	3	3	3	3	3	3
1,4-Dioxane	G	3	3	3	3	3	3	3	3	3	3	3	3
1,4-Dioxane	H	3	3	3	3	3	3	1	4[a]	3	3	3	3

4,4'-bipyridine : 3-hydroxy-2-naphthoic acid : 1,2-bis(4-pyridyl)ethane (A : B : C) plate 2													
Volume of Oil		200 nL											
Volume of Stock Solution (A : B : C)		2 : 2 : 1 (140 nL)				2 : 1 : 2 (140 nL)				1 : 2 : 2 (140 nL)			
Solvent		1	2	3	4	5	6	7	8	9	10	11	12
MeOH	A	3	3	3	3	3	3	3	3	3	3	3	3
MeOH	B	3	3	3	3	3	3	3	3	3	3	3	3
DMF	C	3	3	3	3	3	3	3	3	3	3	3	3
DMF	D	3	3	3	3	3	4*	3	3	3	3	3	3
$MeNO_2$	E	3	3	3	3	3	3	3	3	3	3	3	3
$MeNO_2$	F	3	3	3	3	3	3	3	3	3	3	3	3
1,4-Dioxane	G	3	3	4[a]	3	3	3	3	3	3	3	3	3
1,4-Dioxane	H	3	3	3	3	3	3	3	3	3	3	3	3

4,4'-bipyridine : 3-hydroxy-2-naphthoic acid : 1,2-bis(4-pyridyl)ethane (A : B : C) plate 3													
Volume of Oil		200 nL											
Volume of Stock Solution (A : B : C)		1 : 1 : 1 (140 nL)											
Solvent		1	2	3	4	5	6	7	8	9	10	11	12
MeOH	A	3	3	3	3	3	3	3	3	3	3	3	3
MeOH	B	3	3	3	3	3	3	3	3	3	3	3	3
DMF	C	3	3	3	3	3	3	3	4[a]	3	3	3	3
DMF	D	3	3	3	3	3	3	3	3	4[a]	3	3	3
$MeNO_2$	E	3	3	3	3	3	3	3	3	3	3	3	3
$MeNO_2$	F	3	3	3	3	3	3	3	3	3	3	3	3
1,4-Dioxane	G	3	3	3	3	3	3	3	3	1	3	1	3
1,4-Dioxane	H	3	3	3	3	4[a]	3	3	3	3	3	3	4[a]

SCXRD Full Data Collection and Structure Refinement

* Binary co-crystal (3-hydroxy-2-naphthoic acid: 1,2-bis(4-pyridyl)ethane, 1:0.5) obtained from P2 D6, DMF, MO oil).

SCXRD Unit Cell Analysis

[a] Binary co-crystal (3-hydroxy-2-naphthoic acid: 1,2-bis(4-pyridyl)ethane, 1:0.5).

S4.4.3 Quaternary Co-Crystallisation 96-Well Plate Readouts

2-chlorolresorcinol: tetramethylpyrazine: 2,2’-bithiophene: 1,2-bis(4-pyridyl)ethane

2-chlororesorcinol : tetramethylpyrazine : 2,2'-bithiophene : 1,2-bis(4-pyridyl)ethane (A : B : C : D)													
Volume of Oil		200 nL											
Volume of Stock Solution (A : B : C : D)		1 : 1 : 1 : 1 (140 nL)											
Solvent		1	2	3	4	5	6	7	8	9	10	11	12
MeOH	A	2	2	2	3	3	3	3	3	3	3	3	3
MeOH	B	3	3	3	3	3	3	4	3	3	3	3	3
DMF	C	3	4	3	3	3	4	3	1	1	1	1	3
DMF	D	3	3	3	3	3	3	3	1	4	4	3	3
$MeNO_2$	E	3	3	4	4	4	4	3	3	4	4	4	4
$MeNO_2$	F	2	4	3	4	3	4	3	4	4	3	4	4[a]
1,4-Dioxane	G	2	3	3	3	3	3	3	3	3	3	3	3
1,4-Dioxane	H	2	4*	3	4	3	3	3	3	3	3	3	3

SCXRD Full Data Collection and Structure Refinement

*Quaternary co-crystal (2-chlororesorcinol: tetramethylpyrazine: 2,2’-bithiophene: 1,2-bis(4-pyridyl)ethane, 1:0.5:0.5:0.5; CSD refcode: BESNAR) obtained from well H2 (1,4-Dioxane, FC-40 oil).

SCXRD Unit Cell Analysis

[a] Quaternary co-crystal (2-chlororesorcinol: tetramethylpyrazine: 2,2’-bithiophene: 1,2-bis(4-pyridyl)ethane, 1:0.5:0.5:0.5; CSD refcode: BESNAR)

resorcinol: tetramethylpyrazine: phenazine: pyrene

resorcinol: tetramethylpyrazine: phenazine: pyrene (A : B : C : D)													
Volume of Oil		200 nL											
Volume of Stock Solution (A : B : C : D)		1 : 1 : 1 : 1 (140 nL)											
Solvent		1	2	3	4	5	6	7	8	9	10	11	12
MeOH	A	3	3	4^a	3	3	3	3	4	1	4	4^a	4
MeOH	B	3	4	4^a	3	3	4	3	4^a	3	4	4	3
DMF	C	3	1	1	1	1	3	3	1	1	1	3	1
DMF	D	3	1	1	1	3	3	3	1	1	1	3	3
$MeNO_2$	E	3	4^a	3	3	3	3	3	3	3	3	4^a	3
$MeNO_2$	F	3	3	3	3	3	3	3	4^a	3	4	3	3
1,4-Dioxane	G	3	3	3	3	3	3	4^a	4*	4	4	4	4
1,4-Dioxane	H	4	4^a	4	4	4	4	3	3	3	3	3	3

SCXRD Full Data Collection and Structure Refinement

*Quaternary co-crystal (resorcinol: tetramethylpyrazine: phenazine: pyrene, 2:1:1:2; CSD refcode: JORBEA) obtained from well G8 (1,4-Dioxane and FY oil).

SCXRD Unit Cell Analysis

[a] Quaternary co-crystal (resorcinol: tetramethylpyrazine: phenazine: pyrene, 2:1:1:2; CSD refcode: JORBEA).

2-chlororesorcinol: tetramethylpyrazine: 2,2'-bipyridine: 1,2-bis(4-pyridyl)ethane

2-chlororesorcinol : tetramethylpyrazine : 2,2'-bipyrdine : 1,2-bis(4-pyridyl)ethane (A : B : C : D)													
Volume of Oil		200 nL											
Volume of Stock Solution (A : B : C : D)		1 : 1 : 1 : 1 (140 nL)											
Solvent		1	2	3	4	5	6	7	8	9	10	11	12
MeOH	A	3	4	3	3	4	4*	3	4	4[a]	4	4	4
MeOH	B	3	3	3	3	3	3	3	3	3	3	3	3
DMF	C	3	3	3	3	3	3	3	1	1	1	1	1
DMF	D	3	3	3	3	3	3	3	3	3	3	3	3
$MeNO_2$	E	2	3	3	3	3	3	3	3	3	3	3	3
$MeNO_2$	F	2	3	3	3	3	3	2	3	3	3	3	3
1,4-Dioxane	G	3	3	3	3	3	3	3	4[a]	4	3	3	3
1,4-Dioxane	H	3	3	3	3	3	4[a]	3	4	4	4[a]	4	4

SCXRD Full Data Collection and Structure Refinement

*Quaternary co-crystal (2-chlororesorcinol: tetramethylpyrazine: 2,2'-bipyridine: 1,2-bis(4-pyridyl)ethane, 1: 0.5: 0.5: 0.5; CSD refcode: BESNEV) obtained from well A6 (MeOH, PDMSO oil).

SCXRD Unit Cell Analysis

[a] Quaternary co-crystal (2-chlororesorcinol: tetramethylpyrazine: 2,2'-bipyridine: 1,2-bis(4-pyridyl)ethane 1: 0.5: 0.5: 0.5; CSD refcode BESNEV).

2-bromoresorcinol: tetramethylpyrazine: 2,2’-bipyridine: 1,2-bis(4-pyridyl)ethane

2-bromoresorcinol : tetramethylpyrazine : 2,2'-bipyrdine : 1,2-bis(4-pyridyl)ethane (A : B : C : D)													
**Volume of Oil**		**200 nL**											
**Volume of Stock Solution (A : B : C : D)**		**1 : 1 : 1 : 1 (140 nL)**											
**Solvent**		**1**	**2**	**3**	**4**	**5**	**6**	**7**	**8**	**9**	**10**	**11**	**12**
MeOH	**A**	3	3	3	3	3	3	3	4	4	4*	4	4
MeOH	**B**	3	3	3	3	3	3	3	3	3	3	3	3
DMF	**C**	3	3	3	3	3	3	3	1	1	1	1	1
DMF	**D**	3	3	3	3	4[a]	4	3	4	4[a]	4	4	4
$MeNO_2$	**E**	3	3	3	3	3	3	3	3	3	3	3	3
$MeNO_2$	**F**	2	3	3	3	3	3	2	3	3	3	3	3
1,4-Dioxane	**G**	3	3	3	3	3	3	3	4[a]	3	3	3	3
1,4-Dioxane	**H**	3	4[a]	3	3	3	3	3	4[a]	4	4	4	4

SCXRD Full Data Collection and Structure Refinement

*Quaternary co-crystal (2-bromoresorcinol: tetramethylpyrazine: 2,2’-bipyridine: 1,2-bis(4-pyridyl)ethane, 1: 0.5: 0.5: 0.5; CSD refcode: BESQIC) obtained from well A10 (MeOH, FY oil).

SCXRD Unit Cell Analysis

[a] Quaternary co-crystal (2-bromoresorcinol: tetramethylpyrazine: 2,2’-bipyridine: 1,2-bis(4-pyridyl)ethane, 1: 0.5: 0.5: 0.5; CSD refcode: BESQIC).

2-chlororesorcinol: tetramethylpyrazine: 2,2’-bipyridine: 4,4’-bipyridine

2-chlororesorcinol : tetramethylpyrazine : 2,2'-bipyrdine : 4,4'-bipyridine (A : B : C : D)													
Volume of Oil		200 nL											
Volume of Stock Solution (A : B : C : D)		1 : 1 : 1 : 1 (140 nL)											
Solvent		1	2	3	4	5	6	7	8	9	10	11	12
MeOH	A	2	2	2	2	2	2	2	2	2	3	4	4*
MeOH	B	2	2	2	2	2	2	2	2	2	2	4[a]	3
DMF	C	2	3	3	3	3	3	2	3	3	3	4[a]	3
DMF	D	2	3	3	3	3	3	2	4[a]	3	3	4	3
$MeNO_2$	E	3	3	3	3	3	2	2	3	3	3	3	3
$MeNO_2$	F	3	3	3	3	3	3	2	3	3	3	3	3
1,4-Dioxane	G	2	3	4[a]	4	4	4	2	3	3	4[a]	3	3
1,4-Dioxane	H	2	4[a]	4	4	4	4	2	4	4	4	4[a]	4

SCXRD Full Data Collection and Structure Refinement

*Quaternary co-crystal (2-chlororesorcinol: tetramethylpyrazine: 2,2’-bipyridine: 4,4’-bipyridine, 1: 0.5: 0.5: 0.5) obtained from well A12 (MeOH, FY oil).

SCXRD Unit Cell Analysis

[a]Quaternary co-crystal (2-chlororesorcinol: tetramethylpyrazine: 2,2’-bipyridine: 4,4’-bipyridine, 1: 0.5: 0.5: 0.5).

2-bromoresorcinol: tetramethylpyrazine: 2,2’-bipyridine: 4,4’-bipyridine

2-bromoresorcinol : tetramethylpyrazine : 2,2'-bipyrdine : 4,4'-bipyridine (A : B : C : D)													
Volume of Oil		200 nL											
Volume of Stock Solution (A : B : C : D)		1 : 1 : 1 : 1 (140 nL)											
Solvent		1	2	3	4	5	6	7	8	9	10	11	12
MeOH	A	2	2	3	3	3	3	2	2	2	2	2	2
MeOH	B	2	3	3	3	3	3	3	4[a]	3	3	3	3
DMF	C	3	3	1	4**	3	4[b]	3	1	1	1	1	1
DMF	D	3	3	3	1	3	3	3	1	4[b]	1	4	4[b]
$MeNO_2$	E	3	3	4[a]	4	3	4	3	4[a]	4	3	3	4[a]
$MeNO_2$	F	3	3	3	4	3	3	3	3	3	4	3	3
1,4-Dioxane	G	3	3	3	3	3	3	3	3	3	3	3	3
1,4-Dioxane	H	3	3	3	3	4[a]	3	3	4	4	4*	4	4

SCXRD Full Data Collection and Structure Refinement

* Quaternary co-crystal (2-bromoresorcinol: tetramethylpyrazine: 2,2’-bipyridine: 4,4’-bipyridine, 1: 0.5: 0.5: 0.5) obtained from P1 H10 (1,4-dioxane, MO oil).

** Binary co-crystal (4,4’-bipyridine: 2-bromoresorcinol, 3:2) obtained from P1 C4 (DMF, PDMSO oil).

SCXRD Unit Cell Analysis

[a] Quaternary co-crystal (2-bromoresorcinol: tetramethylpyrazine: 2,2’-bipyridine: 4,4’-bipyridine, 1: 0.5: 0.5: 0.5).

[b] Binary co-crystal (4,4’-bipyridine: 2-bromoresorcinol, 3:2).

2-methylresorcinol: tetramethylpyrazine: 2,2'-bipyridine: 4,4'-bipyridine

2-methylresorcinol : tetramethylpyrazine : 2,2'-bipyrdine : 4,4'-bipyridine (A : B : C : D)													
Volume of Oil		200 nL											
Volume of Stock Solution (A : B : C : D)		1 : 1 : 1 : 1 (140 nL)											
Solvent		1	2	3	4	5	6	7	8	9	10	11	12
MeOH	A	2	4	4	3	4[a]	4	4	3	3	4[a]	3	3
MeOH	B	3	3	4	3	3	3	3	3	3	3	3	3
DMF	C	3	3	4**	3	4**	3	3	4**	4**	4**	3	3
DMF	D	3	3	4**	3	4**	3	3	3	3	3	4**	3
$MeNO_2$	E	4	3	3	3	3	3	4	3	3	3	3	3
$MeNO_2$	F	3	3	3	3	3	3	4	3	3	3	3	3
1,4-Dioxane	G	4	4	3	4	4	4	4	4	4[a]	4	4	3
1,4-Dioxane	H	3	4	4	4	3	4	4	4*	4	4	4	4

SCXRD Full Data Collection and Structure Refinement

* Quaternary co-crystal (2-methylresorcinol: tetramethylpyrazine: 2,2'-bipyridine: 4,4'-bipyridine, 1: 0.5: 0.5: 0.5) obtained from P1 H8 (1,4-dioxane, MO oil).

**Full data collection obtained, structure refinement inconclusive.

SCXRD Unit Cell Analysis

[a] Quaternary co-crystal (2-methylresorcinol: tetramethylpyrazine: 2,2'-bipyridine: 4,4'-bipyridine, 1: 0.5: 0.5: 0.5).

orcinol: tetramethylpyrazine: 2,2’-bipyridine: 4,4’-bipyridine

orcinol : tetramethylpyrazine : 2,2'-bipyrdine : 4,4'-bipyridine (A : B : C : D)													
**Volume of Oil**		**200 nL**											
**Volume of Stock Solution (A : B : C : D)**		**1 : 1 : 1 : 1 (140 nL)**											
**Solvent**		**1**	**2**	**3**	**4**	**5**	**6**	**7**	**8**	**9**	**10**	**11**	**12**
MeOH	**A**	3	3	3	3	3	3	3	3	3	3	3	3
MeOH	**B**	3	3	3	3	3	3	3	3	3	3	3	3
DMF	**C**	3	3	3	3	4	3	3	4[b]	4	4[b]	4[b]	4[b]
DMF	**D**	3	3	4**	3	3	3	3	4	4	4	4	4
$MeNO_2$	**E**	3	4	4	4	4	4	4	4	4	4*	4	4
$MeNO_2$	**F**	4	4[a]	4	4[a]	4	4	4	4	4	4	4	4
1,4-Dioxane	**G**	3	3	3	3	3	3	3	3	4	4	4	4
1,4-Dioxane	**H**	3	4	4	3	3	3	4	4	4	4[a]	4	4[a]

SCXRD Full Data Collection and Structure Refinement

* Quaternary co-crystal (orcinol: tetramethylpyrazine: 2,2’-bipyridine: 4,4’-bipyridine, 1: 0.5: 0.5: 0.5) obtained from P1 E10 ($MeNO_2$, FY oil).

** Binary co-crystal (4,4’-bipyridine: orcinol, 1.5:1; CSD recode: UBUJIM) obtained from P1 D3 (DMF, FC-40 oil).

SCXRD Unit Cell Analysis

[a] Quaternary co-crystal (orcinol: tetramethylpyrazine: 2,2’-bipyridine: 4,4’-bipyridine, 1: 0.5: 0.5: 0.5).

[b] Binary co-crystal (4,4’-bipyridine: orcinol, 1.5:1; CSD refcode: UBUJIM).

Caffeine: 3,5-dinitrobenzoic acid: 2-methylresorcinol: oxalic acid

caffeine : 3,5-dinitrobenzoic acid : 2-methylresorcinol : oxalic acid (A : B : C : D)													
Volume of Oil		200 nL											
Volume of Stock Solution (A : B : C : D)		1 : 1 : 1 : 1 (140 nL)											
Solvent		1	2	3	4	5	6	7	8	9	10	11	12
MeOH	A	3	4[a]	3	3	3	3	3	3	3	3	3	3
MeOH	B	3	3	3	3	3	3	3	4*	3	4	3	3
DMF	C	4[a]	3	3	4	4[a]	3	4	3	3	3	4[a]	4
DMF	D	4	4	4	4[a]	3	4	4	3	3	4	4[a]	3
$MeNO_2$	E	3	3	3	3	3	3	3	3	3	3	3	3
$MeNO_2$	F	3	3	3	4**	3	4	3	4	4	4[b]	4	4
1,4-Dioxane	G	3	3	3	3	3	3	3	3	4	4***	3	3
1,4-Dioxane	H	3	3	3	3	4	4[c]	3	3	3	4	4[c]	3

SCXRD Full Data Collection and Structure Refinement

*Hydrate (oxalic acid hydrate; CSD refcode: OXACBH) obtained from well B8 (MeOH, FC-40).

**Binary co-crystal (caffeine: oxalic acid, 1:0.5; CSD refcode: GANXUP) obtained from well F4 ($MeNO_2$, FC-40 oil).

***Ternary co-crystal (caffeine: 2-methylresorcinol: oxalic acid, 1:1:0.5) obtained from well G10 (1,4-dioxane, FY oil).

SCXRD Unit Cell Analysis

[a] Hydrate (oxalic acid: H_2O, 0.5:1; CSD refcode: OXACBH).

[b] Binary co-crystal (caffeine: oxalic acid, 1:0.5; CSD refcode: GANXUP).

[c] Ternary co-crystal (caffeine: 2-methylresorcinol: oxalic acid, 1:1:0.5).

Caffeine: 3,5-dinitrobenzoic acid: 2-methylresorcinol: 4,4’-dihydroxybiphenyl

caffeine : 3,5-dinitrobenzoic acid : 2-methylresorcinol : 4,4'-dihydroxybiphenyl (A : B : C : D)													
Volume of Oil		200 nL											
Volume of Stock Solution (A : B : C : D)		1 : 1 : 1 : 1 (140 nL)											
**Solvent**		**1**	**2**	**3**	**4**	**5**	**6**	**7**	**8**	**9**	**10**	**11**	**12**
MeOH	**A**	3	3	3	3	3	3	3	3	3	3	3	3
MeOH	**B**	3	3	3	3	3	3	4[a]	3	3	3	3	3
DMF	**C**	4	4	4[a]	3	3	4	4*	1	1	3	4[a]	2
DMF	**D**	3	3	3	3	1	1	4	4	4[a]	4	4	4
$MeNO_2$	**E**	2	3	3	3	3	3	3	3	3	3	3	3
$MeNO_2$	**F**	2	3	3	3	3	3	2	3	3	3	3	3
1,4-Dioxane	**G**	3	3	3	3	3	3	3	4	4[b]	4	4	4
1,4-Dioxane	**H**	3	3	3	3	3	3	3	4	4[b]	4	4	4

SCXRD Full Data Collection and Structure Refinement

*Single-component crystal (4,4’-dihydroxybiphenyl; CSD refcode: DOHDPH02), obtained from well P1 C7 (DMF, no oil).

SCXRD Unit Cell Analysis

[a] Single-component crystal (4,4’-dihydroxybiphenyl; CSD refcode: DOHDPH02).

[b] Ternary co-crystal hydrate (caffeine: 3,5-dinitrobenzoic acid: 2-methylresorcinol: H_2O, 1:1:2:1).

Caffeine: 3,5-dinitrobenzoic acid: 2-methylresorcinol: resorcinol

caffeine : 3,5-dinitrobenzoic acid : 2-methylresorcinol : resorcinol (A : B : C : D)													
Volume of Oil		200 nL											
Volume of Stock Solution (A : B : C : D)		1 : 1 : 1 : 1 (140 nL)											
Solvent		1	2	3	4	5	6	7	8	9	10	11	12
MeOH	A	2	2	2	2	2	2	2	2	2	2	2	2
MeOH	B	2	2	2	2	2	2	2	2	3	3	2	2
DMF	C	2	2	2	2	2	2	2	2	2	2	2	2
DMF	D	2	2	2	2	2	2	2	2	2	2	2	3
$MeNO_2$	E	2	3	3	2	2	2	2	2	2	2	3	3
$MeNO_2$	F	2	2	3	3	2	3	2	2	2	3	3	2
1,4-Dioxane	G	2	2	3	3	2	2	3	2	2	2	3	2
1,4-Dioxane	H	2	3	2	2	2	2	2	2	2	2	3	2

caffeine: 3,5-dinitrobenzoic acid: 2-methylresorcinol: quinol

caffeine : 3,5-dinitrobenzoic acid : 2-methylresorcinol : quinol (A : B : C : D)													
Volume of Oil		200 nL											
Volume of Stock Solution (A : B : C : D)		1 : 1 : 1 : 1 (140 nL)											
Solvent		1	2	3	4	5	6	7	8	9	10	11	12
MeOH	A	3	3	3	3	3	3	3	2	3	3	3	3
MeOH	B	3	3	3	3	3	4[a]	3	4[a]	4	4	4	3
DMF	C	2	2	2	2	2	2	2	2	2	3	3	1
DMF	D	2	2	2	2	2	2	3	2	2	3	3	3
$MeNO_2$	E	3	3	3	3	3	3	3	3	3	3	3	3
$MeNO_2$	F	3	3	3	3	3	3	3	4[b]	3	3	3	3
1,4-Dioxane	G	3	3	3	3	3	3	3	3	3	2	3	2
1,4-Dioxane	H	3	3	3	3	4	4[b]	3	4	3	3	4	2

SCXRD Unit Cell Analysis

[a] Single-component crystal (quinol; CSD refcode: HYQUIN04).

[b] Ternary co-crystal hydrate (caffeine: 3,5-dinitrobenzoic acid: 2-methylresorcinol: H_2O, 1:1:2:1).

Caffeine: 3,5-dinitrobenzoic acid: 2-methylresorcinol: orcinol

caffeine : 3,5-dinitrobenzoic acid : 2-methylresorcinol : orcinol (A : B : C : D)													
Volume of Oil		200 nL											
Volume of Stock Solution (A : B : C : D)		1 : 1 : 1 : 1 (140 nL)											
Solvent		1	2	3	4	5	6	7	8	9	10	11	12
MeOH	A	3	3	3	3	4*	2	4[a]	2	2	3	1	3
MeOH	B	3	3	3	2	3	2	3	3	3	3	3	3
DMF	C	2	2	2	2	2	2	2	2	2	2	2	2
DMF	D	2	2	2	2	2	2	2	2	2	2	2	2
$MeNO_2$	E	3	4**	3	4**	3	4**	3	3	1	2	2	3
$MeNO_2$	F	3	2	2	4**	2	3	3	3	3	2	3	3
1,4-Dioxane	G	3	3	3	3	3	3	3	2	2	2	2	2
1,4-Dioxane	H	3	2	2	2	2	3	3	2	3	2	3	3

SCXRD Full Data Collection and Structure Refinement

*Binary co-crystal hydrate (3,5-dinitrobenzoic acid: orcinol: H_2O, 2:2:2) obtained from well A5 (MeOH, PDMSO oil).

** Full data collection obtained, structure refinement inconclusive.

SCXRD Unit Cell Analysis

[a] Binary co-crystal hydrate (3,5-dinitrobenzoic acid: orcinol: H_2O, 2:2:2).

caffeine: 3,5-dinitrobenzoic acid: 2-methylresorcinol: 2-bromoresorcinol

caffeine : 3,5-dinitrobenzoic acid : 2-methylresorcinol : 2-bromoresorcinol (A : B : C : D)													
Volume of Oil		200 nL											
Volume of Stock Solution (A : B : C : D)		1 : 1 : 1 : 1 (140 nL)											
Solvent		1	2	3	4	5	6	7	8	9	10	11	12
MeOH	A	3	4[a]	4	3	3	4[a]	3	3	1	1	3	3
MeOH	B	3	1	1	3	4[a]	1	3	3	3	1	1	1
DMF	C	1	1	1	1	1	1	1	1	1	1	1	1
DMF	D	1	1	1	1	1	1	1	1	1	1	1	1
$MeNO_2$	E	3	3	3	3	3	3	3	3	1	3	1	3
$MeNO_2$	F	3	3	3	3	3	3	3	3	3	3	3	3
1,4-Dioxane	G	3	3	3	3	3	3	3	3	3	3	3	3
1,4-Dioxane	H	3	3	3	3	3	3	3	3	4[a]	4[a]	4[a]	3

SCXRD Unit Cell Analysis

[a] Single component crystal (3,5-dinitrobenzoic acid).

caffeine: 3,5-dinitrobenzoic acid: 2-methylresorcinol: 2-chlororesorcinol

caffeine : 3,5-dinitrobenzoic acid : 2-methylresorcinol : 2-chlororesorcinol (A : B : C : D)													
Volume of Oil		200 nL											
Volume of Stock Solution (A : B : C : D)		1 : 1 : 1 : 1 (140 nL)											
Solvent		1	2	3	4	5	6	7	8	9	10	11	12
MeOH	A	3	4[a]	3	4[a]	3	3	4	1	3	3	3	3
MeOH	B	3	3	3	3	4[a]	4	3	4	3	3	1	3
DMF	C	1	1	1	1	1	1	1	1	1	1	1	1
DMF	D	1	1	1	1	1	1	1	1	1	1	1	1
$MeNO_2$	E	3	4[a]	3	3	3	3	3	1	1	1	1	1
$MeNO_2$	F	2	4	4[a]	4	4	3	3	3	3	4	4[a]	3
1,4-Dioxane	G	2	4	3	3	3	3	3	4	4	3	4	4
1,4-Dioxane	H	3	3	3	4[a]	3	3	3	3	4[a]	3	4[b]	4

SCXRD Unit Cell Analysis

[a] Single component crystal (3,5-dinitrobenzoic acid).

[b] Ternary co-crystal hydrate (caffeine: 3,5-dinitrobenzoic acid: 2-methylresorcinol: H_2O (1:1:2:1).

caffeine: 3,5-dinitrobenzoic acid: 2-methylresorcinol: 4-chlorobenzene-1,3-diol

caffeine : 3,5-dinitrobenzoic acid : 2-methylresorcinol : 4-chlorobenzene-1,3-diol (A : B : C : D)													
Volume of Oil		200 nL											
Volume of Stock Solution (A : B : C : D)		1 : 1 : 1 : 1 (140 nL)											
Solvent		1	2	3	4	5	6	7	8	9	10	11	12
MeOH	A	3	3	3	3	3	3	3	1	1	3	1	1
MeOH	B	3	1	1	1	1	1	3	3	1	1	3	1
DMF	C	3	1	1	1	1	1	1	1	1	1	1	1
DMF	D	1	1	1	1	1	1	1	1	1	1	1	1
$MeNO_2$	E	3	3	3	3	3	3	3	3	3	3	1	1
$MeNO_2$	F	2	3	3	3	3	3	2	3	3	3	3	3
1,4-Dioxane	G	3	3	3	3	3	4[a]	3	3	4[a]	3	3	3
1,4-Dioxane	H	3	2	2	2	3	2	2	4[a]	4	4	4[a]	4

SCXRD Unit Cell Analysis

[a] Single component crystal (3,5-dinitrobenzoic acid).

caffeine: 3,5-dinitrobenzoic acid: 2-methylresorcinol: 4-bromobenzene-1,3-diol

caffeine : 3,5-dinitrobenzoic acid : 2-methylresorcinol : 4-bromo-1,3-benzenediol (A : B : C : D)													
Volume of Oil		200 nL											
Volume of Stock Solution (A : B : C : D)		1 : 1 : 1 : 1 (140 nL)											
Solvent		1	2	3	4	5	6	7	8	9	10	11	12
MeOH	A	3	3	3	3	3	3	3	1	1	1	1	1
MeOH	B	3	3	3	3	3	1	3	1	3	3	3	1
DMF	C	1	1	1	1	1	1	1	1	1	1	1	1
DMF	D	1	1	1	1	1	1	1	1	1	1	1	1
$MeNO_2$	E	2	2	2	2	2	2	2	3	1	1	3	1
$MeNO_2$	F	2	3	3	3	3	3	3	3	3	3	3	3
1,4-Dioxane	G	2	3	3	3	3	3	2	3	3	3	3	3
1,4-Dioxane	H	3	3	3	3	3	3	2	4	4[a]	4	4[a]	4

SCXRD Unit Cell Analysis

[a] Single component crystal (3,5-dinitrobenzoic acid).

caffeine: 3,5-dinitrobenzoic acid: 2-methylresorcinol: 4-methylbenzene-1,3-diol

caffeine : 3,5-dinitrobenzoic acid : 2-methylresorcinol : 4-methyl-1,3-benzenediol (A : B : C : D)													
Volume of Oil		200 nL											
Volume of Stock Solution (A : B : C : D)		1 : 1 : 1 : 1 (140 nL)											
Solvent		1	2	3	4	5	6	7	8	9	10	11	12
MeOH	A	3	3	3	3	3	3	3	1	1	1	3	1
MeOH	B	3	3	3	3	3	1	3	1	3	3	3	3
DMF	C	1	1	1	1	1	1	1	1	1	1	1	1
DMF	D	1	1	1	1	1	1	1	1	1	1	1	1
$MeNO_2$	E	3	3	3	3	3	3	3	3	3	3	3	3
$MeNO_2$	F	3	3	3	3	3	3	3	3	3	3	3	3
1,4-Dioxane	G	3	3	4[a]	4	4[a]	3	3	3	3	3	3	3
1,4-Dioxane	H	3	3	4[a]	3	3	3	3	3	3	3	3	3

SCXRD Unit Cell Analysis

[a] Single component crystal (3,5-dinitrobenzoic acid).

4,4'-bipyridine: methyl gallate: 2-chlororesorcinol: 1,2-bis(4-pyridyl)ethane

4,4'-bipyridine : methyl gallate : 2-chlororesorcinol : 1,2-bis(4-pyridyl)ethane (A : B : C : D)													
Volume of Oil		200 nL											
Volume of Stock Solution (A : B : C : D)		1 : 1 : 1 : 1 (140 nL)											
Solvent		1	2	3	4	5	6	7	8	9	10	11	12
MeOH	A	3	3	3	3	3	3	3	4*	4[a]	1	1	1
MeOH	B	3	3	3	3	3	3	2	3	3	3	3	2
DMF	C	1	1	1	1	1	1	3	1	1	1	1	3
DMF	D	1	3	3	1	1	1	1	2	1	1	1	1
$MeNO_2$	E	3	3	4	4[b]	4	4	4	4	4[b]	4	4[b]	4
$MeNO_2$	F	4[b]	4	4[b]	4	4	4	4	4	4	4[b]	4	4
1,4-Dioxane	G	3	2	3	3	3	3	3	4[c]	1	4[c]	3	3
1,4-Dioxane	H	3	1	1	3	3	3	3	3	3	3	3	3

SCXRD Full Data Collection and Structure Refinement

* Ternary co-crystal (4,4'-bipyridine: methyl gallate: 2-chlororesorcinol, 3:1:1) obtained from well P1 A8 (MeOH, FY oil).

SCXRD Unit Cell Analysis

[a] Ternary co-crystal (4,4'-bipyridine: methyl gallate: 2-chlororesorcinol, 3:1:1).

[b] Binary co-crystal solvate hydrate (4,4'-bipyridine: methyl gallate: $MeNO_2$: H_2O, 3:2:2:2).

[c] Ternary co-crystal hydrate (methyl gallate: 4,4'-bipyridine: 2-chlororesorcinol: H_2O, 2:3:1:2).

4,4'-bipyridine: methyl gallate: 2-chlororesorcinol: [2,2'-bipyridine]-4,4-diyldimethanol

4,4'-bipyridine : methyl gallate : 2-chlororesorcinol : [2,2'-bipyridine]-4,4'-diyldimethanol (A : B : C : D)													
Volume of Oil		200 nL											
Volume of Stock Solution (A : B : C : D)		1 : 1 : 1 : 1 (140 nL)											
Solvent		1	2	3	4	5	6	7	8	9	10	11	12
MeOH	A	3	3	3	3	3	3	3	4[a]	4	3	3	4
MeOH	B	3	3	3	3	3	3	3	3	3	3	4[a]	4
DMF	C	3	4	4[b]	4	3	4	3	4[b]	4	4[b]	4	4
DMF	D	3	3	3	3	3	3	3	4*	3	3	3	4
$MeNO_2$	E	4	4[c]	4	4	4	4	4[c]	4	4	4[c]	4	4
$MeNO_2$	F	3	4	4	4[c]	4	4	4	4	4	4	4[c]	4
1,4-Dioxane	G	3	3	3	3	3	3	3	3	3	4[d]	4[d]	3
1,4-Dioxane	H	3	4[a]	4	4	4[a]	4	3	3	3	3	3	3

SCXRD Full Data Collection and Structure Refinement

* Binary co-crystal (methyl gallate: [2,2'-bipyridine]-4,4-diyldimethanol, 1:0.5) obtained from well D8 (DMF, MO oil).

SCXRD Unit Cell Analysis

[a] Ternary co-crystal hydrate (methyl gallate: 4,4'-bipyridine: 2-chlororesorcinol: H_2O, 2:3:1:2).

[b] Binary co-crystal (methyl gallate: [2,2'-bipyridine]-4,4-diyldimethanol, 1:0.5).

[c] Binary co-crystal solvate hydrate (4,4'-bipyridine: methyl gallate: $MeNO_2$: H_2O, 3:2:2:2).

[d] Single-component crystal (methyl gallate; CSD refcode: ROMGAC).

4,4’-bipyridine: methyl gallate: 2-chlororesorcinol: tetramethylpyrazine

4,4'-bipyridine : methyl gallate : 2-chlororesorcinol : tetramethylpyrazine (A : B : C : D)													
Volume of Oil		200 nL											
Volume of Stock Solution (A : B : C : D)		1 : 1 : 1 : 1 (140 nL)											
Solvent		1	2	3	4	5	6	7	8	9	10	11	12
MeOH	A	3	3	3	3	3	3	3	3	3	3	3	3
MeOH	B	3	3	3	3	3	3	3	3	3	3	3	3
DMF	C	3	4[a]	4	3	3	3	3	4	3	4[a]	3	4
DMF	D	3	3	3	3	3	3	4[a]	4[a]	3	3	3	4
$MeNO_2$	E	3	4[b]	3	3	3	4	3	4[b]	4[b]	4	4	4[b]
$MeNO_2$	F	3	4	4[b]	4	4	4	3	3	4	4	4	4[b]
1,4-Dioxane	G	3	3	3	3	3	3	3	3	3	3	3	3
1,4-Dioxane	H	3	3	3	3	3	3	3	3	3	3	3	3

SCXRD Unit Cell Analysis

[a] Binary co-crystal hydrate (4,4’-bipyridine: methyl gallate: H_2O, 2:2:3).

[b] Binary co-crystal solvate hydrate (4,4’-bipyridine: methyl gallate: $MeNO_2$: H_2O, 3:2:2:2).

4,4'-bipyridine: methyl gallate: 2-chlororesorcinol: 2,2'-bipyridine-4,4'-biphenylcarboxylic acid

4,4'-bipyridine : methyl gallate : 2-chlororesorcinol : 2,2'-bipyridine-4,4'-biphenylcarboxylic acid (A : B : C : D)													
**Volume of Oil**		**200 nL**											
**Volume of Stock Solution (A : B : C : D)**		**1 : 1 : 1 : 1 (140 nL)**											
**Solvent**		**1**	**2**	**3**	**4**	**5**	**6**	**7**	**8**	**9**	**10**	**11**	**12**
MeOH	**A**	3	3	3	3	3	3	2	4	4[a]	3	4	4[a]
MeOH	**B**	2	3	3	3	3	3	2	3	3	3	3	3
DMF	**C**	3	3	3	4[b]	4	4[b]	3	3	3	3	3	4[c]
DMF	**D**	3	3	3	3	3	4[c]	3	3	3	3	3	3
$MeNO_2$	**E**	3	3	3	3	3	3	3	4	4	4	4	4[b]
$MeNO_2$	**F**	3	4[d]	4	4	4	4	3	4	4	4[d]	4	4
1,4-Dioxane	**G**	3	3	3	3	3	3	3	4[a]	4	4	4	4
1,4-Dioxane	**H**	3	3	3	3	3	3	3	3	3	3	3	3

SCXRD Unit Cell Analysis

[a] Ternary co-crystal hydrate (methyl gallate: 4,4'-bipyridine: 2-chlororesorcinol: H_2O, 2:3:1:2).

[b] Binary co-crystal hydrate (4,4'-bipyridine: methyl gallate: H_2O, 2:2:3).

[c] Binary co-crystal solvate hydrate (4,4'-bipyridine: methyl gallate: DMF: H_2O, 3:2:1:2).

[d] Binary co-crystal solvate hydrate (4,4'-bipyridine: methyl gallate: $MeNO_2$: H_2O, 3:2:2:2).

4,4’-bipyridine: methyl gallate: 2-chlororesorcinol: [1,1’-biphenyl]-4,4’-diyldimethanol

4,4'-bipyridine : methyl gallate : 2-chlororesorcinol : [1,1'-biphenyl]-4,4'-diyldimethanol (A : B : C : D)													
Volume of Oil		200 nL											
Volume of Stock Solution (A : B : C : D)		1 : 1 : 1 : 1 (140 nL)											
Solvent		1	2	3	4	5	6	7	8	9	10	11	12
MeOH	A	3	3	3	3	3	3	3	3	4[a]	4	3	3
MeOH	B	3	3	3	3	3	3	3	3	4[a]	3	3	3
DMF	C	3	3	3	3	3	4[b]	3	3	3	3	4[b]	3
DMF	D	3	3	3	3	3	3	3	3	3	3	3	3
$MeNO_2$	E	4	3	3	3	4[c]	3	3	3	4	4	4[c]	4
$MeNO_2$	F	3	4	4	4[c]	4	4	4[c]	3	3	4	4	4[c]
1,4-Dioxane	G	3	3	3	3	3	3	3	4	4	4[a]	4	4
1,4-Dioxane	H	3	4	3	4[a]	3	4	3	3	3	3	3	3

SCXRD Unit Cell Analysis

[a] Binary co-crystal hydrate (4,4’-bipyridine: methyl gallate: H_2O, 2:2:3).

[b] Binary co-crystal solvate hydrate 4,4’-bipyridine: methyl gallate: DMF: H_2O, 3:2:1:2).

[c] Binary co-crystal solvate hydrate (4,4’-bipyridine: methyl gallate: $MeNO_2$: H_2O, 3:2:2:2).

4,4’-bipyridine: methyl gallate: 2-chlororesorcinol: propyl gallate

4,4'-bipyridine : methyl gallate : 2-chlororesorcinol : propyl gallate (A : B : C : D)													
Volume of Oil		200 nL											
Volume of Stock Solution (A : B : C : D)		1 : 1 : 1 : 1 (140 nL)											
Solvent		1	2	3	4	5	6	7	8	9	10	11	12
MeOH	A	3	3	3	3	3	3	3	3	3	3	3	3
MeOH	B	2	1	3	3	3	3	2	3	3	3	3	3
DMF	C	3	3	2	2	2	2	1	3	1	2	3	3
DMF	D	3	3	1	3	2	1	1	3	1	3	1	1
$MeNO_2$	E	3	4	4[a]	3	3	4	3	3	4*	3	4[a]	4
$MeNO_2$	F	3	3	3	3	3	3	3	4[a]	4[a]	4	4	4
1,4-Dioxane	G	3	3	3	3	3	3	3	4	3	4	4	4
1,4-Dioxane	H	3	4	4	4[a]	3	3	3	3	3	3	3	4[a]

SCXRD Full Data Collection and Structure Refinement

* Binary co-crystal (4,4’-bipyridine: propyl gallate, 1:1) obtained from well E9 ($MeNO_2$, FY oil).

SCXRD Unit Cell Analysis

[a] Binary co-crystal (4,4’-bipyridine: propyl gallate, 1:1).

4,4'-bipyridine: methyl gallate: 2-chlororesorcinol: pyrogallol

4,4'-bipyridine : methyl gallate : 2-chlororesorcinol : pyrogallol (A : B : C : D)													
Volume of Oil		200 nL											
Volume of Stock Solution (A : B : C : D)		1 : 1 : 1 : 1 (140 nL)											
Solvent		1	2	3	4	5	6	7	8	9	10	11	12
MeOH	A	3	4	3	4[a]	4	3	3	2	3	4[a]	4	3
MeOH	B	4[a]	3	3	3	3	3	3	3	3	3	3	3
DMF	C	2	2	2	2	2	2	2	2	2	2	2	2
DMF	D	2	2	2	2	2	2	2	2	2	2	2	2
$MeNO_2$	E	2	3	3	3	3	3	3	3	4[b]	3	4	3
$MeNO_2$	F	3	3	3	3	3	3	3	4[b]	4	3	3	4
1,4-Dioxane	G	4[a]	4	4	3	4[a]	4	3	4	4	4	4[a]	4
1,4-Dioxane	H	4	4[a]	4	3	4	4	3	4	4[a]	4	4	3

SCXRD Unit Cell Analysis

[a] Binary co-crystal hydrate (4,4'-bipyridine: methyl gallate: H_2O (2:2:3).

[b] Binary co-crystal solvate hydrate (4,4'-bipyridine: methyl gallate: $MeNO_2$: H_2O, (3:2:2:2).

S4.5 Cross-Polarised Optical Microscopy Images of Crystals for which full SCXRD Analysis was Performed

**Binary co-crystals**		
**Previously known binary co-crystals from binary co-crystal screening**		
1	4,4'-bipyridine: 2,4-dihydroxybenzoic acid<br>P1 C7, MeOH, FY (1:1)<br>CSD refcode: IDUBUF	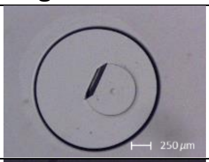
2	4,4'-bipyridine: 3,5-dinitrobenzoic acid<br>P1 A2, MeOH, PDMSO (2:1)<br>CSD refcode: FIHYEA	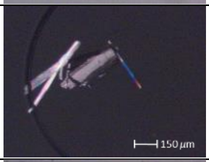
3	4,4'-bipyridine: glutaric acid<br>P1 D12, MeOH, MO (1:2)<br>CSD refcode: SOVDIQ	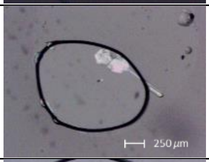
4	4,4'-bipyridine: 3-hydroxy-2-naphthoic acid<br>P1 C7, MeOH, FY (1:1)<br>CSD refcode: GEHROB	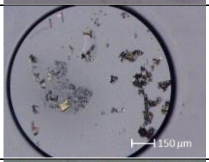
5	4,4'-bipyridine: quinol<br>P2 F7, 1,4-Dioxane, FC-40 (1:1)<br>CSD refcode: QAMRUS	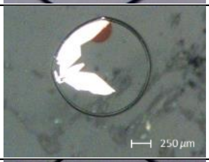
6	caffeine: 2,4-dihydroxybenzoic acid<br>P2 G10, 1,4-dioxane, FY (1:2)<br>CSD refcode: MOZCIO	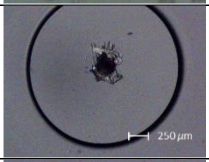
7	caffeine: glutaric acid<br>P2 A9, $MeNO_2$ (1:2)<br>CSD refcode: EXUQUJ01	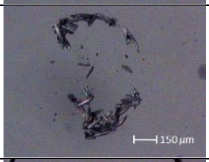
8	caffeine: 3-hydroxy-2-naphthoic acid<br>P2 G2, 1,4-dioxane, FY (2:1)<br>CSD refcode: KIGKOB	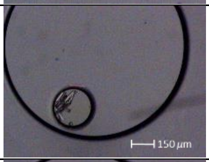
9	caffeine: methyl gallate<br>P1 C12, MeOH, FY (1:2)<br>CSD refcode: DIJVOH	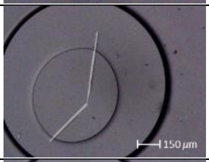
10	nicotinamide: 2,4-dihydroxybenzoic acid MeOH solvate<br>P1 C8, MeOH, FY (1:1)<br>CSD refcode: DINSEA	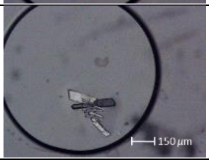

11	nicotinamide: 2,4-dihydroxybenzoic acid<br>P2 C3, $MeNO_2$, FY (2:1)<br>CSD refcode: DINRUP01	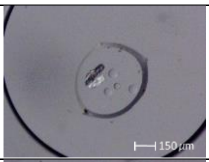
12	nicotinamide: glutaric acid<br>P1 D11, MeOH, MO (1:2)<br>CSD refcode: NUKYEY	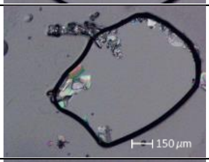
13	nicotinamide: 3-hydroxy-2-naphthoic acid<br>P1 A12, MeOH, PDMSO (1:2)<br>CSD refcode: ABULEQ	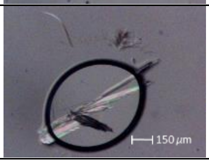
	**New binary co-crystals from binary co-crystal screening**	
14	4,4'-bipyridine: 3,5-dinitrobenzoic acid<br>P2 B7, $MeNO_2$, FC-40 (1:1)	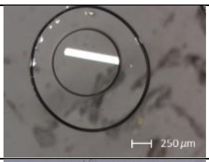
15	4,4'-bipyridine: 3-hydroxy-2-naphthoic acid<br>P2 A8, $MeNO_2$, PDMSO (1:1)	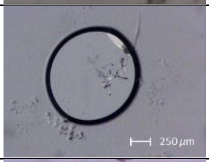
16	4,4'-bipyridine: methyl gallate<br>P1 B3, MeOH, FC-40 (2:1)	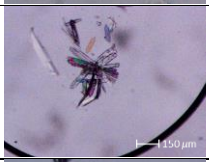
17	4,4'-bipyridine: methyl gallate<br>P1 F4, DMF, FC-40 (2:1)	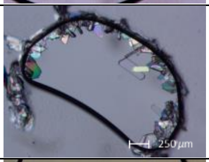
18	4,4'-bipyridine: methyl gallate<br>P2 D4, $MeNO_2$, MO (2:1)	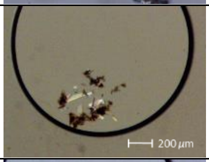
19	caffeine: 3,5-dinitrobenzoic acid<br>P1 G7, DMF, FY (1:1)	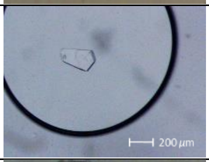
20	caffeine: quinol<br>P2 F11, 1,4-Dioxane, FC-40 (1:2)	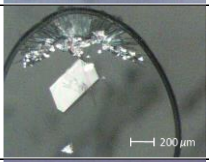
21	nicotinamide: 3,5-dinitrobenzoic acid<br>P1 C12, MeOH, FY (1:2)	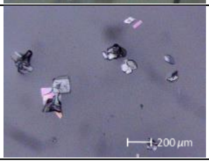

22	nicotinamide: methyl gallate<br>P2 C6, $MeNO_2$, FY (1:1)	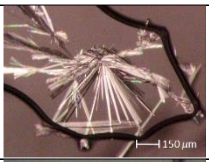
23	nicotinamide: quinol<br>P1 D2, MeOH, MO (2:1)	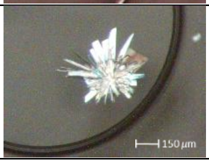
**Binary co-crystals from ternary and quaternary co-crystal screening**		
**Previously known binary co-crystals from ternary and quaternary co-crystal screening**		
24	4,4’-bipyridine: 3,3’-thiodipropinoic acid<br>(4,4’-bipyridine: glutaric acid: 3,3’-thiodipropinoic acid)<br>P3 C5, DMF, PDMSO (1:1:1)<br>CSD refcode: SOVHEQ	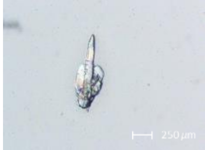
25	Quinol: tetramethylpyrazine<br>(caffeine: quinol: tetramethylpyrazine)<br>P2 B9, MeOH, FY (1:2:2)<br>CSD refcode: COZZOH	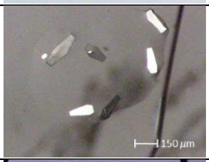
26	Caffeine: oxalic acid<br>(caffeine: 3,5-dinitrobenzoic acid: 2-methylresorcinol: oxalic acid)<br>P1 F4, $MeNO_2$, FC-40 (1:1:1:1)<br>CSD refcode: GANXUP	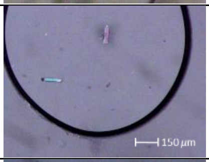
27	4,4’-bipyridine: orcinol<br>(4,4’-bipyridine: orcinol: phenazine)<br>P1 C6, DMF, FY (1:2:1)<br>CSD refcode: UBUJIM	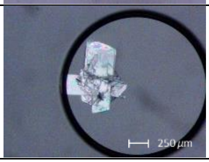
**New binary co-crystals from ternary and quaternary co-crystal screening**		
28	orcinol: phenazine<br>(4,4’-bipyridine: orcinol: phenazine)<br>P1 G8, 1,4-dioxane, FY (1:2:1)	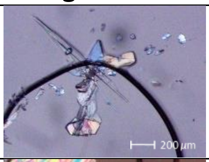
29	nicotinamide: quinol<br>(nicotinamide: quinol: nicotinic acid)<br>P3 H9, 1,4-dioxane, FY (1:1:1)	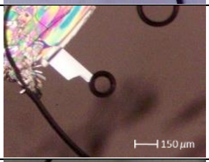
30	3-hydroxy-2-naphthoic acid: 1,2-bis(4-pyridyl)ethane<br>(4,4’-bipyridine: 3-hydroxy-2-naphthoic acid: 1,2-bis(4-pyridyl)ethane)<br>P2 D6, DMF, MO (2:1:2)	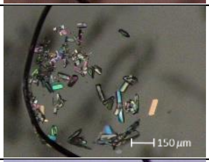
31	methyl gallate: [2,2’-bipyridine]-4,4-diyldimethanol<br>(4,4’-bipyridine: methyl gallate: 2-chlororesorcinol: [2,2’-bipyridine]-4,4-diyldimethanol)<br>P1 D8, DMF, MO (1:1:1:1)	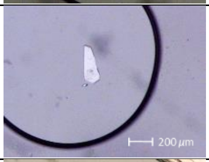
32	3,5-dinitrobenzoic acid: orcinol hydrate<br>Caffeine: 3,5-dinitrobenzoic acid: 2-methylresorcinol: orcinol<br>P1 A5, MeOH, PDMSO (1:1:1:1)	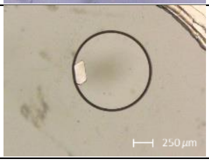

33	4,4'-bipyridine: propyl gallate<br>(4,4'-bipyridine: methyl gallate: 2-chlororesorcinol: propyl gallate)<br>P1 E9, MeNO2, FY (1:1:1:1)	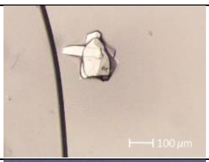
34	4,4'-bipyridine: 2-bromoresorcinol<br>(2-bromoresorcinol: tetramethylpyrazine: 2,2'-bipyridine: 4,4'-bipyridine)<br>P1 C4, DMF, PDMSO (1:1:1:1)	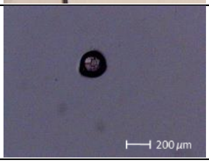

Ternary Co-crystals		
**Previously known ternary co-crystals from ternary co-crystal screening**		
35	toluic acid: isonicotinamide: 3,5-dinitrobenzoic acid<br>P1 F1, $MeNO_2$, FC-40 (2:1:1)<br>CSD refcode: BUDZUV	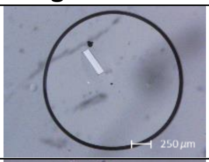
36	4,4′-bipyridine: orcinol: phenazine<br>P2 D6, DMF, MO (2:1:2)<br>CSD refcode: UBUKEJ	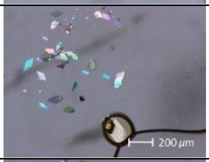
37	nicotinamide: fumaric acid: isoniazid<br>P2 D11, DMF, FC-40 (1:2:2)<br>CSD refcode: BICQEL	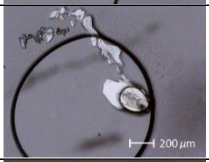
38	tetramethylpyrazine: 2,2′-bipyridine: 2-chlororesorcinol<br>P3 E10, $MeNO_2$, FY (1:1:1)<br>CSD refcode: BESNOF	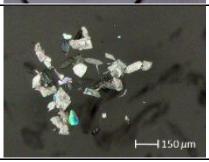
**New ternary co-crystals from ternary co-crystal screening**		
39	tetramethylpyrazine: 2,2′-bipyridine: 2-chlororesorcinol<br>P2 F5, $MeNO_2$, FC-40 (2:1:2)	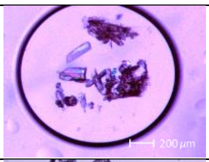
40	4,4′-bipyridine: methyl gallate: 2-chlororesorcinol<br>P1 A10, MeOH, PDMSO (1:1:2)	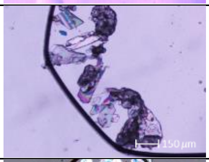
41	caffeine: 3,5-dinitrobenzoic acid: 2-methylresorcinol<br>P3 E6, $MeNO_2$, PDMSO (1:1:1).	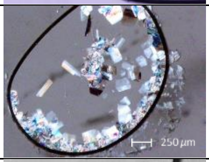
42	nicotinamide: 3,5-dinitrobenzoic acid: glutaric acid<br>P3 E2, $MeNO_2$, PDMSO (1:1:1)	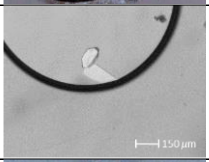
43	nicotinamide: 3,5-dinitrobenzoic acid: tetramethylpyrazine<br>P1 A6, MeOH, FY (1:2:1)	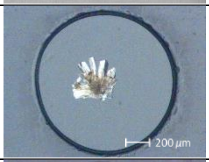
44	nicotinamide: quinol: benzoic acid<br>P3 H12, 1,4-Dioxane, MO (1:1:1)	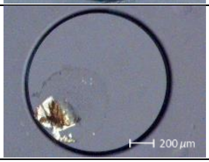
**New ternary co-crystals from quaternary co-crystal screening**		
45	4,4′-bipyridine: methyl gallate: 2-chloroquinol<br>(4,4′-bipyridine: methyl gallate: 2-chloroquinol: 1,2-bis(4-pyridyl)ethane)<br>P1 A8, MeOH, FY (1:1:1:1)	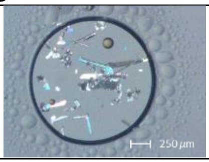

46	caffeine: 2-methylresorcinol: oxalic acid<br>(caffeine: 3,5-dinitrobenzoic acid: 2-methylresorcinol: oxalic acid)<br>P1 G10, 1,4-dioxane, FY (1:1:1:1)	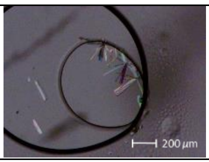

Quaternary Co-crystals		
**Previously known quaternary co-crystals from quaternary co-crystal screening**		
47	2-chlororesorcinol: tetramethylpyrazine: 2,2’-bithiophene: 1,2-bis(4-pyridyl)ethane<br>P1 H2, 1,4-Dioxane, FC-40 (1:1:1:1)<br>CSD refcode: BESNAR	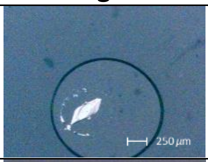
48	resorcinol: tetramethylpyrazine: phenazine: pyrene<br>P1 G8, 1,4-Dioxane, FY (1:1:1:1)<br>CSD refcode: JORBEA	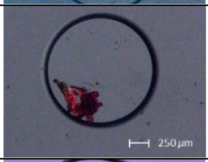
49	2-chlororesorcinol: tetramethylpyrazine: 2,2’-bipyridine: 1,2-bis(4-pyridyl)ethane<br>P1 A10, MeOH, PDMSO (1:1:1:1)<br>CSD refcode: BESNEV	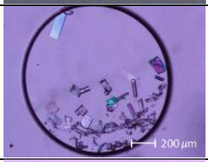
50	2-bromoresorcinol: tetramethylpyrazine: 2,2’-bipyridine: 1,2-bis(4-pyridyl)ethane<br>P1 H2, MeOH, FY (1:1:1:1)<br>CSD refcode: BESQIC	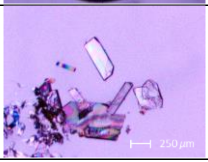
**New quaternary co-crystals from quaternary co-crystal screening**		
51	2-chlororesorcinol: tetramethylpyrazine: 2,2’-bipyridine: 4,4’-bipyridine<br>P1 A12, MeOH, FY (1:1:1:1)	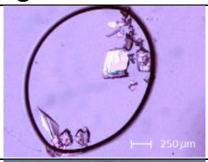
52	2-bromoresorcinol: tetramethylpyrazine: 2,2’-bipyridine: 4,4’-bipyridine<br>P1 H10, 1,4-dioxane, MO (1:1:1:1)	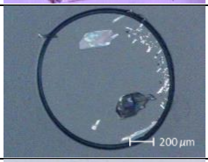
53	2-methylresorcinol: tetramethylpyrazine: 2,2’-bipyridine: 4,4’-bipyridine<br>P1 H8, 1,4-dioxane, MO (1:1:1:1)	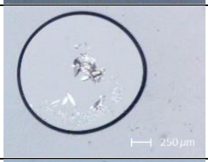
54	orcinol: tetramethylpyrazine: 2,2’-bipyridine: 4,4’-bipyridine<br>P1 E10, $MeNO_2$, FY (1:1:1:1)	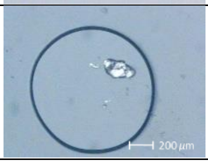

S5. Crystal Data and Structure Refinement Details

S5.1 Previously Known Binary Co-Crystals from Binary Co-Crystal Screening

CCDC Deposition Number: 2372065

4,4'-bipyridine: 2,4-dihydroxybenzoic acid (1:1)

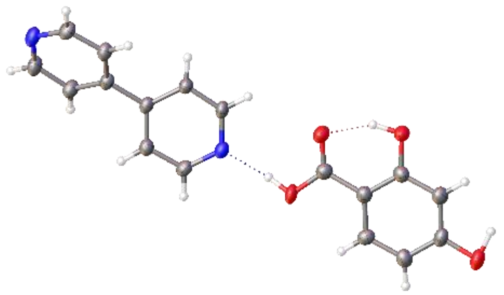

Empirical formula	$C_{17}H_{14}N_2O_4$
Formula weight	310.30
Temperature/K	150.00
Crystal system	monoclinic
Space group	$P2_1/c$
a/Å	6.5495(4)
b/Å	10.7984(7)
c/Å	20.6573(13)
α/°	90
β/°	97.076(6)
γ/°	90
Volume/Å^3	1449.84(16)
Z	4
ρ_{calc}g/cm^3	1.422
μ/mm^{-1}	0.855
F(000)	648.0
Crystal size/mm^3	0.062 × 0.035 × 0.015
Radiation	Cu Kα (λ = 1.54184)
2Θ range for data collection/°	8.626 to 141.91
Index ranges	-7 ≤ h ≤ 7, -12 ≤ k ≤ 12, -22 ≤ l ≤ 25
Reflections collected	7423
Independent reflections	2703 [R_{int} = 0.0250, R_{sigma} = 0.0308]
Data/restraints/parameters	2703/273/318
Goodness-of-fit on F^2	1.178
Final R indexes [I>=2σ (I)]	R_1 = 0.0481, wR_2 = 0.1164
Final R indexes [all data]	R_1 = 0.0560, wR_2 = 0.1204
Largest diff. peak/hole / e Å^{-3}	0.22/-0.17

CCDC Deposition Number: 2372066

4,4'-bipyridine: 3,5-dinitrobenzoic acid (0.5:1)

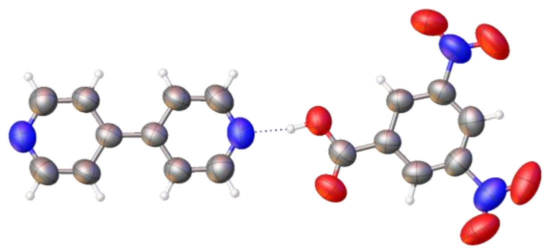

Empirical formula	$C_{12}H_8N_3O_6$
Formula weight	290.21
Temperature/K	294.98(17)
Crystal system	monoclinic
Space group	$P2_1/n$
a/Å	6.2329(7)
b/Å	22.008(3)
c/Å	9.4129(11)
α/°	90
β/°	99.411(10)
γ/°	90
Volume/Å^3	1273.9(3)
Z	4
ρ_{calc}g/cm^3	1.513
μ/mm^{-1}	1.076
F(000)	596.0
Crystal size/mm^3	0.31 × 0.023 × 0.017
Radiation	Cu Kα (λ = 1.54184)
2Θ range for data collection/°	8.034 to 133.19
Index ranges	-4 ≤ h ≤ 7, -26 ≤ k ≤ 26, -11 ≤ l ≤ 11
Reflections collected	8471
Independent reflections	2234 [R_{int} = 0.0387, R_{sigma} = 0.0393]
Data/restraints/parameters	2234/145/193
Goodness-of-fit on F^2	1.089
Final R indexes [I>=2σ (I)]	R_1 = 0.0593, wR_2 = 0.1570
Final R indexes [all data]	R_1 = 0.0832, wR_2 = 0.1716
Largest diff. peak/hole / e Å^{-3}	0.18/-0.17

CCDC Deposition Number: 2372067

4,4’-bipyridine: glutaric acid (2:2)

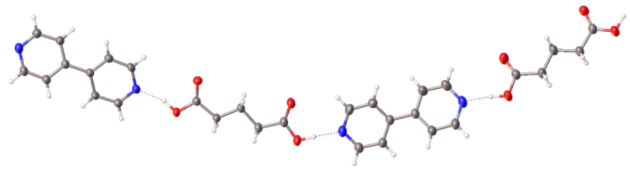

Empirical formula	$C_{15}H_{16}N_2O_4$
Formula weight	288.30
Temperature/K	149.99(10)
Crystal system	monoclinic
Space group	$P2_1/c$
a/Å	5.55880(10)
b/Å	9.9773(3)
c/Å	50.1061(16)
α/°	90
β/°	92.693(2)
γ/°	90
Volume/Å^3	2775.91(13)
Z	8
ρ_{calc}g/cm^3	1.380
μ/mm^{-1}	0.842
F(000)	1216.0
Crystal size/mm^3	0.19 × 0.062 × 0.024
Radiation	Cu Kα (λ = 1.54184)
2Θ range for data collection/°	7.064 to 145.976
Index ranges	-6 ≤ h ≤ 6, -12 ≤ k ≤ 7, -61 ≤ l ≤ 61
Reflections collected	25349
Independent reflections	5328 [R_{int} = 0.0321, R_{sigma} = 0.0276]
Data/restraints/parameters	5328/276/391
Goodness-of-fit on F^2	1.070
Final R indexes [I>=2σ (I)]	R_1 = 0.0440, wR_2 = 0.1155
Final R indexes [all data]	R_1 = 0.0574, wR_2 = 0.1242
Largest diff. peak/hole / e Å^{-3}	0.28/-0.20

CCDC Deposition Number: 2372068

4,4’-bipyridine: 3-hydroxy-2-naphthoic acid (0.5:1)

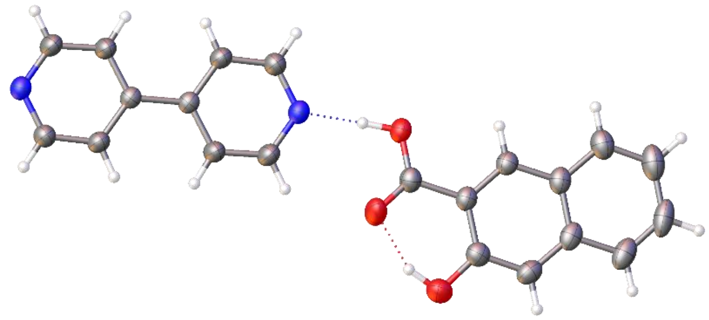

Empirical formula	$C_{16}H_{12}NO_3$
Formula weight	266.27
Temperature/K	149.99(10)
Crystal system	monoclinic
Space group	$P2_1/c$
a/Å	8.9136(4)
b/Å	11.3523(5)
c/Å	12.4921(5)
α/°	90
β/°	98.270(4)
γ/°	90
Volume/Å^3	1250.93(9)
Z	4
ρ_{calc}g/cm^3	1.414
μ/mm^{-1}	0.809
F(000)	556.0
Crystal size/mm^3	0.12 × 0.086 × 0.03
Radiation	Cu Kα (λ = 1.54184)
2Θ range for data collection/°	10.028 to 153.938
Index ranges	$-10 \le h \le 11$, $-12 \le k \le 13$, $-15 \le l \le 14$
Reflections collected	15848
Independent reflections	2481 [R_{int} = 0.0361, R_{sigma} = 0.0241]
Data/restraints/parameters	2481/145/187
Goodness-of-fit on F^2	1.069
Final R indexes [I>=2σ (I)]	R_1 = 0.0448, wR_2 = 0.1163
Final R indexes [all data]	R_1 = 0.0561, wR_2 = 0.1249
Largest diff. peak/hole / e Å^{-3}	0.26/-0.24

CCDC Deposition Number: 2372069

4,4'-bipyridine: quinol (1:0.5)

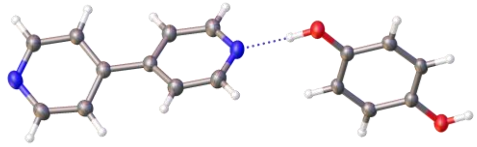

Empirical formula	$C_{13}H_{11}N_2O$
Formula weight	211.24
Temperature/K	150.01(10)
Crystal system	triclinic
Space group	P-1
a/Å	7.8241(4)
b/Å	8.6299(5)
c/Å	9.2154(6)
α/°	112.063(6)
β/°	109.788(6)
γ/°	94.584(5)
Volume/Å^3	527.10(6)
Z	2
ρ_{calc}g/cm^3	1.331
μ/mm^{-1}	0.693
F(000)	222.0
Crystal size/mm^3	0.23 × 0.1 × 0.028
Radiation	Cu Kα (λ = 1.54184)
2Θ range for data collection/°	11.298 to 155.608
Index ranges	-9 ≤ h ≤ 9, -10 ≤ k ≤ 10, -10 ≤ l ≤ 11
Reflections collected	5155
Independent reflections	2042 [R_{int} = 0.0238, R_{sigma} = 0.0295]
Data/restraints/parameters	2042/102/148
Goodness-of-fit on F^2	1.079
Final R indexes [I>=2σ (I)]	R_1 = 0.0400, wR_2 = 0.1101
Final R indexes [all data]	R_1 = 0.0441, wR_2 = 0.1136
Largest diff. peak/hole / e Å^{-3}	0.18/-0.22

CCDC Deposition Number: 2372070

caffeine: 2,4-dihydroxybenzoic acid: H_2O (1:1:1)

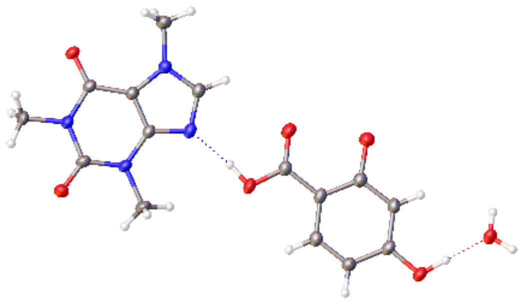

Empirical formula	$C_{15}H_{18}N_4O_7$
Formula weight	366.33
Temperature/K	150.00
Crystal system	triclinic
Space group	P-1
a/Å	7.1718(4)
b/Å	8.5853(5)
c/Å	13.7480(6)
α/°	90.289(4)
β/°	100.961(4)
γ/°	106.499(5)
Volume/Å^3	795.26(8)
Z	2
ρ_{calc}g/cm^3	1.530
μ/mm^{-1}	1.051
F(000)	384.0
Crystal size/mm^3	0.17 × 0.012 × 0.007
Radiation	Cu Kα (λ = 1.54184)
2Θ range for data collection/°	6.562 to 142.68
Index ranges	-8 ≤ h ≤ 6, -10 ≤ k ≤ 10, -16 ≤ l ≤ 16
Reflections collected	6338
Independent reflections	2949 [R_{int} = 0.0318, R_{sigma} = 0.0497]
Data/restraints/parameters	2949/192/253
Goodness-of-fit on F^2	1.050
Final R indexes [I>=2σ (I)]	R_1 = 0.0480, wR_2 = 0.1220
Final R indexes [all data]	R_1 = 0.0620, wR_2 = 0.1290
Largest diff. peak/hole / e Å^{-3}	0.34/-0.23

CCDC Deposition Number: 2372071

caffeine: glutaric acid (1:1)

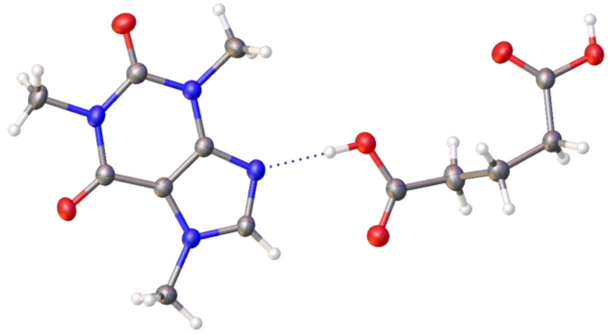

Empirical formula	$C_{13}H_{18}N_4O_6$
Formula weight	326.31
Temperature/K	149.99(10)
Crystal system	monoclinic
Space group	$P2_1/c$
a/Å	13.0056(3)
b/Å	6.5779(2)
c/Å	17.0942(4)
α/°	90
β/°	97.832(2)
γ/°	90
Volume/Å^3	1448.76(7)
Z	4
ρ_{calc}g/cm^3	1.496
μ/mm^{-1}	1.021
F(000)	688.0
Crystal size/mm^3	0.12 × 0.089 × 0.031
Radiation	Cu Kα (λ = 1.54184)
2Θ range for data collection/°	6.86 to 152.094
Index ranges	-16 ≤ h ≤ 15, -8 ≤ k ≤ 7, -21 ≤ l ≤ 15
Reflections collected	8658
Independent reflections	2826 [R_{int} = 0.0269, R_{sigma} = 0.0292]
Data/restraints/parameters	2826/165/217
Goodness-of-fit on F^2	1.034
Final R indexes [I>=2σ (I)]	R_1 = 0.0377, wR_2 = 0.1044
Final R indexes [all data]	R_1 = 0.0472, wR_2 = 0.1116
Largest diff. peak/hole / e Å^{-3}	0.25/-0.23

CCDC Deposition Number: 2372072

caffeine: 3-hydroxy-2-naphthoic acid (1:1)

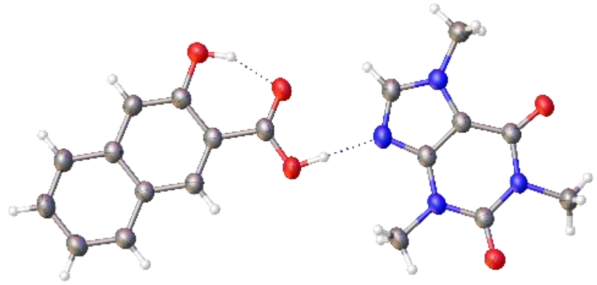

Empirical formula	$C_{19}H_{18}N_4O_5$
Formula weight	382.37
Temperature/K	150.00
Crystal system	monoclinic
Space group	$P2_1/c$
a/Å	9.0412(5)
b/Å	24.4254(10)
c/Å	8.6209(5)
α/°	90
β/°	116.710(7)
γ/°	90
Volume/Å^3	1700.65(18)
Z	4
ρ_{calc}g/cm^3	1.493
μ/mm^{-1}	0.925
F(000)	800.0
Crystal size/mm^3	0.15 × 0.11 × 0.079
Radiation	Cu Kα (λ = 1.54184)
2Θ range for data collection/°	7.238 to 155.162
Index ranges	-10 ≤ h ≤ 11, -19 ≤ k ≤ 30, -10 ≤ l ≤ 10
Reflections collected	11468
Independent reflections	3384 [R_{int} = 0.0310, R_{sigma} = 0.0256]
Data/restraints/parameters	3384/222/262
Goodness-of-fit on F^2	1.059
Final R indexes [I>=2σ (I)]	R_1 = 0.0395, wR_2 = 0.1090
Final R indexes [all data]	R_1 = 0.0427, wR_2 = 0.1116
Largest diff. peak/hole / e Å^{-3}	0.29/-0.19

CCDC Deposition Number: 2372073

caffeine: methyl gallate (1:1)

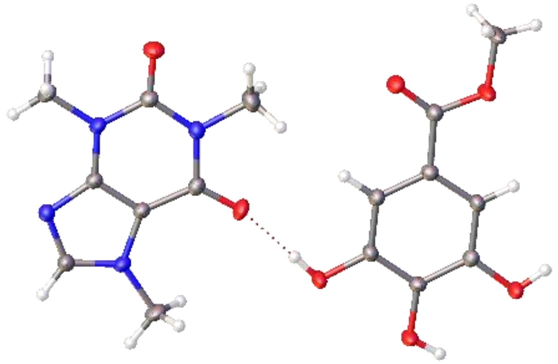

Empirical formula	$C_{16}H_{18}N_4O_7$
Formula weight	378.34
Temperature/K	150.00
Crystal system	monoclinic
Space group	$P2_1/n$
a/Å	6.9229(2)
b/Å	10.1175(3)
c/Å	23.0844(7)
α/°	90
β/°	91.161(2)
γ/°	90
Volume/Å^3	1616.56(8)
Z	4
ρ_{calc}g/cm^3	1.555
μ/mm^{-1}	1.057
F(000)	792.0
Crystal size/mm^3	0.4 × 0.17 × 0.1
Radiation	Cu Kα (λ = 1.54184)
2Θ range for data collection/°	7.662 to 133.144
Index ranges	-8 ≤ h ≤ 6, -9 ≤ k ≤ 12, -27 ≤ l ≤ 27
Reflections collected	8578
Independent reflections	2833 [R_{int} = 0.0145, R_{sigma} = 0.0158]
Data/restraints/parameters	2833/207/258
Goodness-of-fit on F^2	1.056
Final R indexes [I>=2σ (I)]	R_1 = 0.0330, wR_2 = 0.0946
Final R indexes [all data]	R_1 = 0.0347, wR_2 = 0.0960
Largest diff. peak/hole / e Å^{-3}	0.23/-0.23

CCDC Deposition Number: 2372074

nicotinamide: 2,4-dihydroxybenzoic acid: MeOH (1:1:1)

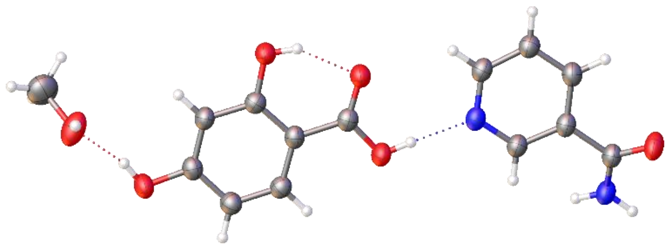

Empirical formula	$C_{14}H_{16}N_2O_6$
Formula weight	308.29
Temperature/K	149.99(10)
Crystal system	triclinic
Space group	P-1
a/Å	7.2390(2)
b/Å	7.5083(2)
c/Å	15.0443(4)
α/°	77.229(2)
β/°	80.117(2)
γ/°	65.746(3)
Volume/Å^3	724.07(4)
Z	2
ρ_{calc}g/cm^3	1.414
μ/mm^{-1}	0.950
F(000)	324.0
Crystal size/mm^3	0.19 × 0.16 × 0.092
Radiation	Cu Kα (λ = 1.54184)
2Θ range for data collection/°	6.048 to 152.808
Index ranges	-8 ≤ h ≤ 9, -9 ≤ k ≤ 9, -18 ≤ l ≤ 18
Reflections collected	14600
Independent reflections	2852 [R_{int} = 0.0267, R_{sigma} = 0.0189]
Data/restraints/parameters	2852/141/218
Goodness-of-fit on F^2	1.045
Final R indexes [I>=2σ (I)]	R_1 = 0.0393, wR_2 = 0.1112
Final R indexes [all data]	R_1 = 0.0429, wR_2 = 0.1139
Largest diff. peak/hole / e Å^{-3}	0.33/-0.28

CCDC Deposition Number: 2372075

nicotinamide: 2,4-dihydroxybenzoic acid (1:1)

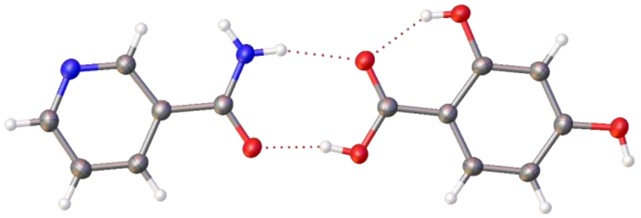

Empirical formula	$C_{13}H_{12}N_2O_5$
Formula weight	276.25
Temperature/K	150.00
Crystal system	triclinic
Space group	P-1
a/Å	5.7882(2)
b/Å	8.0510(3)
c/Å	13.3821(5)
α/°	96.039(3)
β/°	91.946(3)
γ/°	94.003(3)
Volume/Å^3	618.11(4)
Z	2
ρ_{calc}g/cm^3	1.484
μ/mm^{-1}	0.984
F(000)	288.0
Crystal size/mm^3	0.17 × 0.11 × 0.031
Radiation	Cu Kα (λ = 1.54184)
2Θ range for data collection/°	6.648 to 153.554
Index ranges	-7 ≤ h ≤ 6, -10 ≤ k ≤ 8, -16 ≤ l ≤ 15
Reflections collected	5731
Independent reflections	2403 [R_{int} = 0.0217, R_{sigma} = 0.0285]
Data/restraints/parameters	2403/138/196
Goodness-of-fit on F^2	1.072
Final R indexes [I>=2σ (I)]	R_1 = 0.0351, wR_2 = 0.0965
Final R indexes [all data]	R_1 = 0.0413, wR_2 = 0.1000
Largest diff. peak/hole / e Å^{-3}	0.20/-0.20

CCDC Deposition Number: 2372076

nicotinamide: glutaric acid (1:1)

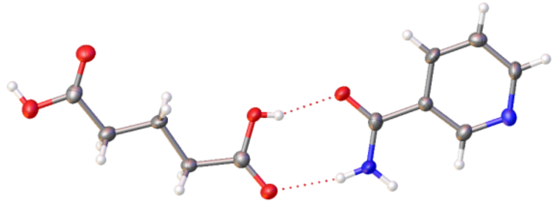

Empirical formula	$C_{11}H_{14}N_2O_5$
Formula weight	254.24
Temperature/K	150
Crystal system	triclinic
Space group	P-1
a/Å	5.4572(2)
b/Å	7.3636(3)
c/Å	15.1899(11)
α/°	99.538(5)
β/°	94.453(4)
γ/°	104.740(4)
Volume/Å^3	577.54(5)
Z	2
ρ_{calc}g/cm^3	1.462
μ/mm^{-1}	0.992
F(000)	268.0
Crystal size/mm^3	0.2 × 0.1 × 0.026
Radiation	Cu Kα (λ = 1.54184)
2Θ range for data collection/°	5.948 to 153.662
Index ranges	-6 ≤ h ≤ 6, -9 ≤ k ≤ 9, -19 ≤ l ≤ 19
Reflections collected	8283
Independent reflections	8283 [R_{int} = 0269, R_{sigma} = 0.0181]
Data/restraints/parameters	8283/111/176
Goodness-of-fit on F^2	1.047
Final R indexes [I>=2σ (I)]	R_1 = 0.0392, wR_2 = 0.1070
Final R indexes [all data]	R_1 = 0.0436, wR_2 = 0.1096
Largest diff. peak/hole / e Å^{-3}	0.26/-0.18

CCDC Deposition Number: 2372077

nicotinamide: 3-hydroxy-2-naphthoic acid (1:1)

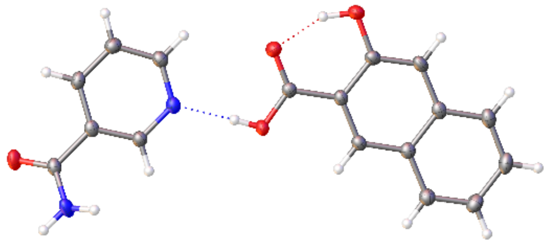

Empirical formula	$C_{17}H_{14}N_2O_4$
Formula weight	310.30
Temperature/K	150.00
Crystal system	monoclinic
Space group	C2/c
a/Å	25.4599(10)
b/Å	4.8787(2)
c/Å	23.7344(9)
α/°	90
β/°	107.608(4)
γ/°	90
Volume/Å^3	2810.0(2)
Z	8
ρ_{calc}g/cm^3	1.467
μ/mm^{-1}	0.882
F(000)	1296.0
Crystal size/mm^3	0.12 × 0.042 × 0.033
Radiation	Cu Kα (λ = 1.54184)
2Θ range for data collection/°	7.286 to 154.406
Index ranges	-29 ≤ h ≤ 31, -5 ≤ k ≤ 3, -29 ≤ l ≤ 29
Reflections collected	9127
Independent reflections	2808 [R_{int} = 0.0176, R_{sigma} = 0.0194]
Data/restraints/parameters	2808/169/220
Goodness-of-fit on F^2	1.047
Final R indexes [I>=2σ (I)]	R_1 = 0.0316, wR_2 = 0.0825
Final R indexes [all data]	R_1 = 0.0359, wR_2 = 0.0851
Largest diff. peak/hole / e Å^{-3}	0.24/-0.22

S5.2 New Binary Co-Crystals from Binary Co-Crystal Screening

CCDC Deposition Number: 2372078

4,4'-bipyridine: 3,5-dinitrobenzoic acid (1:1)

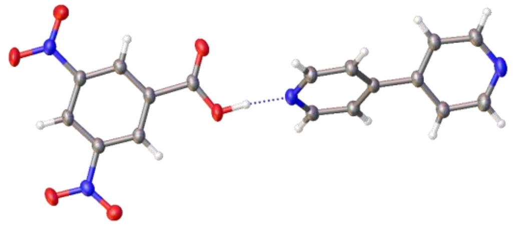

Empirical formula	$C_{17}H_{12}N_4O_6$
Formula weight	368.31
Temperature/K	150.01(10)
Crystal system	monoclinic
Space group	$P2_1/c$
a/Å	13.6498(5)
b/Å	7.3982(2)
c/Å	16.4817(5)
α/°	90
β/°	106.994(3)
γ/°	90
Volume/Å^3	1591.71(9)
Z	4
ρ_{calc}g/cm^3	1.537
μ/mm^{-1}	1.018
F(000)	760.0
Crystal size/mm^3	0.28 × 0.077 × 0.064
Radiation	Cu Kα (λ = 1.54184)
2Θ range for data collection/°	6.772 to 155.622
Index ranges	-16 ≤ h ≤ 16, -9 ≤ k ≤ 3, -19 ≤ l ≤ 20
Reflections collected	9803
Independent reflections	3096 [R_{int} = 0.0267, R_{sigma} = 0.0295]
Data/restraints/parameters	3096/196/247
Goodness-of-fit on F^2	1.035
Final R indexes [I>=2σ (I)]	R_1 = 0.0438, wR_2 = 0.1206
Final R indexes [all data]	R_1 = 0.0492, wR_2 = 0.1264
Largest diff. peak/hole / e Å^{-3}	0.34/-0.37

CCDC Deposition Number: 2372079

4,4’-bipyridine: 3-hydroxy-2-naphthoic acid (1.5:1)

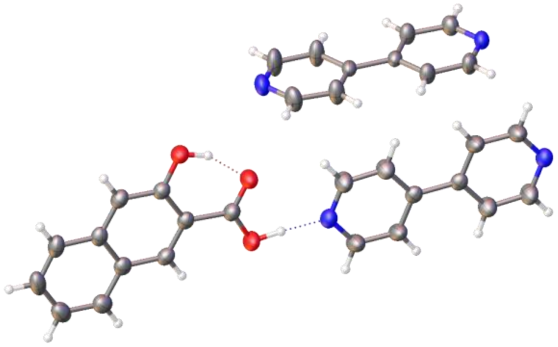

Empirical formula	$C_{26}H_{20}N_3O_3$
Formula weight	422.45
Temperature/K	150.00
Crystal system	triclinic
Space group	P-1
a/Å	9.7342(6)
b/Å	10.4465(5)
c/Å	10.8557(7)
α/°	77.280(5)
β/°	86.238(5)
γ/°	72.443(5)
Volume/Å^3	1026.65(11)
Z	2
ρ_{calc}g/cm^3	1.367
μ/mm^{-1}	0.737
F(000)	442.0
Crystal size/mm^3	0.12 × 0.062 × 0.034
Radiation	Cu Kα (λ = 1.54184)
2Θ range for data collection/°	8.35 to 142.662
Index ranges	-11 ≤ h ≤ 11, -12 ≤ k ≤ 12, -13 ≤ l ≤ 8
Reflections collected	8392
Independent reflections	3810 [R_{int} = 0.0231, R_{sigma} = 0.0320]
Data/restraints/parameters	3810/232/296
Goodness-of-fit on F^2	1.051
Final R indexes [I>=2σ (I)]	R_1 = 0.0455, wR_2 = 0.1119
Final R indexes [all data]	R_1 = 0.0577, wR_2 = 0.1188
Largest diff. peak/hole / e Å^{-3}	0.20/-0.18

CCDC Deposition Number: 2372080

4,4'-bipyridine: methyl gallate: H_2O (2:2:3)

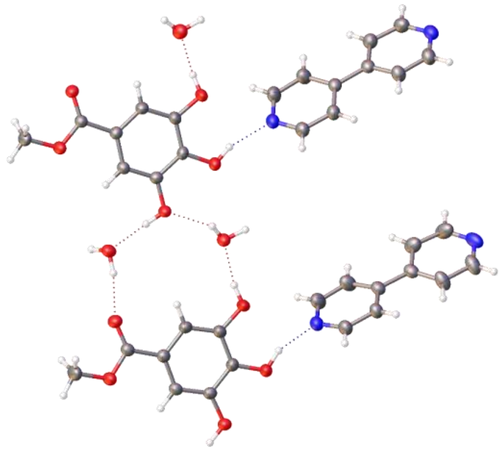

Empirical formula	$C_{36}H_{38}N_4O_{13}$
Formula weight	734.70
Temperature/K	149.99(10)
Crystal system	triclinic
Space group	P-1
a/Å	9.5205(2)
b/Å	12.1056(3)
c/Å	16.1549(3)
α/°	99.092(2)
β/°	97.390(2)
γ/°	105.510(2)
Volume/Å^3	1742.73(7)
Z	2
ρ_{calc}g/cm^3	1.400
μ/mm^{-1}	0.907
F(000)	772.0
Crystal size/mm^3	0.1 × 0.076 × 0.031
Radiation	Cu Kα (λ = 1.54184)
2Θ range for data collection/°	5.632 to 152.756
Index ranges	-12 ≤ h ≤ 11, -12 ≤ k ≤ 15, -20 ≤ l ≤ 19
Reflections collected	19549
Independent reflections	6898 [R_{int} = 0.0237, R_{sigma} = 0.0292]
Data/restraints/parameters	6898/361/516
Goodness-of-fit on F^2	1.044
Final R indexes [I>=2σ (I)]	R_1 = 0.0385, wR_2 = 0.1051
Final R indexes [all data]	R_1 = 0.0491, wR_2 = 0.1127
Largest diff. peak/hole / e Å^{-3}	0.21/-0.19

CCDC Deposition Number: 2372081

4,4'-bipyridine: methyl gallate: DMF: H_2O (3:2:1:2)

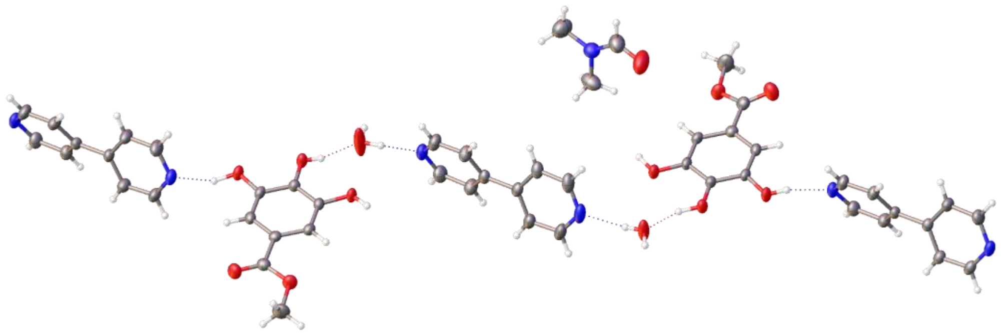

Empirical formula	$C_{49}H_{51}N_7O_{13}$
Formula weight	945.96
Temperature/K	150.00
Crystal system	monoclinic
Space group	Cc
a/Å	9.4553(2)
b/Å	17.6614(4)
c/Å	28.0494(5)
α/°	90
β/°	93.143(2)
γ/°	90
Volume/Å^3	4677.03(17)
Z	4
ρ_{calc}g/cm^3	1.343
μ/mm^{-1}	0.821
F(000)	1992.0
Crystal size/mm^3	0.11 × 0.059 × 0.033
Radiation	Cu Kα (λ = 1.54184)
2Θ range for data collection/°	6.312 to 154.544
Index ranges	-11 ≤ h ≤ 6, -22 ≤ k ≤ 21, -34 ≤ l ≤ 33
Reflections collected	17067
Independent reflections	6028 [R_{int} = 0.0185, R_{sigma} = 0.0200]
Data/restraints/parameters	6028/475/665
Goodness-of-fit on F^2	1.049
Final R indexes [I>=2σ (I)]	R_1 = 0.0274, wR_2 = 0.0749
Final R indexes [all data]	R_1 = 0.0282, wR_2 = 0.0753
Largest diff. peak/hole / e Å^{-3}	0.16/-0.17
Flack parameter	0.11(8)

CCDC Deposition Number: 2372082

4,4'-bipyridine: methyl gallate: $MeNO_2$: H_2O (3:2:2:2)

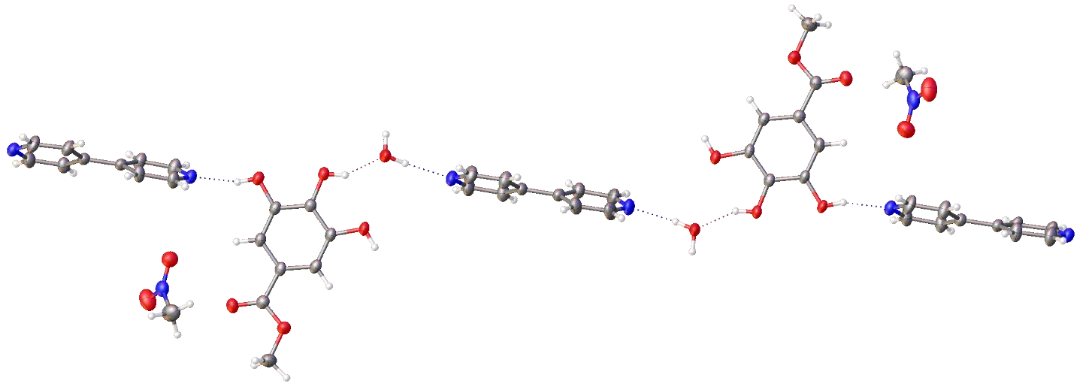

Empirical formula	$C_{48}H_{50}N_8O_{16}$
Formula weight	994.96
Temperature/K	150.00(10)
Crystal system	monoclinic
Space group	$P2_1$
a/Å	9.68930(10)
b/Å	16.7367(2)
c/Å	15.0094(2)
α/°	90
β/°	99.9370(10)
γ/°	90
Volume/Å^3	2397.51(5)
Z	2
ρ_{calc}g/cm^3	1.378
μ/mm^{-1}	0.884
F(000)	1044.0
Crystal size/mm^3	0.06 × 0.048 × 0.038
Radiation	Cu Kα (λ = 1.54184)
2Θ range for data collection/°	5.978 to 154.366
Index ranges	-12 ≤ h ≤ 12, -20 ≤ k ≤ 20, -18 ≤ l ≤ 18
Reflections collected	29451
Independent reflections	9408 [R_{int} = 0.0367, R_{sigma} = 0.0365]
Data/restraints/parameters	9408/484/665
Goodness-of-fit on F^2	1.040
Final R indexes [I>=2σ (I)]	R_1 = 0.0428, wR_2 = 0.1164
Final R indexes [all data]	R_1 = 0.0481, wR_2 = 0.1200
Largest diff. peak/hole / e Å^{-3}	0.32/-0.26
Flack parameter	0.46(7)

CCDC Deposition Number: 2372083

caffeine: 3,5-dinitrobenzoic acid (2:2)

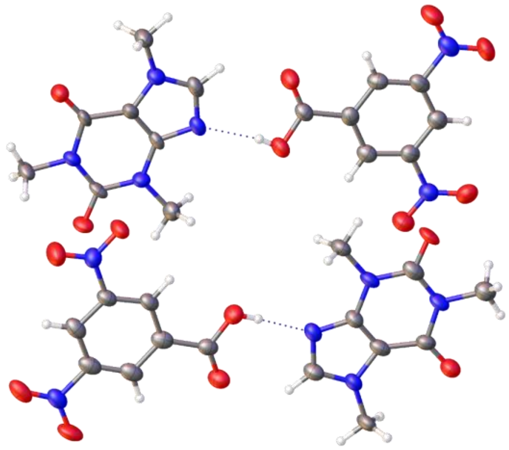

Empirical formula	$C_{15}H_{14}N_6O_8$
Formula weight	406.32
Temperature/K	149.99(10)
Crystal system	orthorhombic
Space group	$P2_12_12_1$
a/Å	9.0637(2)
b/Å	29.4253(8)
c/Å	12.6604(4)
α/°	90
β/°	90
γ/°	90
Volume/Å^3	3376.56(16)
Z	8
ρ_{calc}g/cm^3	1.599
μ/mm^{-1}	1.144
F(000)	1680.0
Crystal size/mm^3	0.17 × 0.072× 0.019
Radiation	Cu Kα (λ = 1.54184)
2Θ range for data collection/°	6.008 to 153.086
Index ranges	-7 ≤ h ≤ 10, -36 ≤ k ≤ 36, -15 ≤ l ≤ 15
Reflections collected	18418
Independent reflections	6539 [R_{int} = 0.0387, R_{sigma} = 0.0419]
Data/restraints/parameters	6539/444/531
Goodness-of-fit on F^2	1.123
Final R indexes [I>=2σ (I)]	R_1 = 0.0597, wR_2 = 0.1454
Final R indexes [all data]	R_1 = 0.0669, wR_2 = 0.1493
Largest diff. peak/hole / e Å^{-3}	0.47/-0.24
Flack parameter	0.50(12)

CCDC Deposition Number: 2372084

caffeine: quinol (1:1.5)

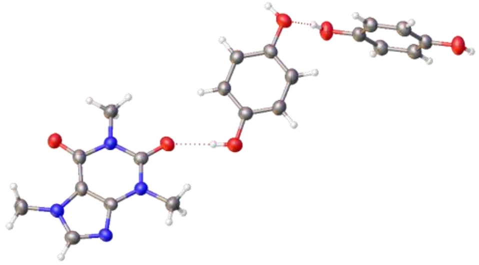

Empirical formula	$C_{17}H_{19}N_4O_5$
Formula weight	359.36
Temperature/K	150.15
Crystal system	triclinic
Space group	P-1
a/Å	8.2386(6)
b/Å	9.1933(6)
c/Å	12.2557(7)
α/°	109.346(5)
β/°	104.319(6)
γ/°	92.860(5)
Volume/Å^3	839.75(10)
Z	2
ρ_{calc}g/cm^3	1.421
μ/mm^{-1}	0.894
F(000)	378.0
Crystal size/mm^3	0.22 × 0.13 × 0.045
Radiation	Cu Kα (λ = 1.54184)
2Θ range for data collection/°	7.964 to 158.306
Index ranges	-10 ≤ h ≤ 7, -11 ≤ k ≤ 11, -15 ≤ l ≤ 14
Reflections collected	7978
Independent reflections	3272 [R_{int} = 0.0240, R_{sigma} = 0.0299]
Data/restraints/parameters	3272/352/284
Goodness-of-fit on F^2	1.050
Final R indexes [I>=2σ (I)]	R_1 = 0.0567, wR_2 = 0.1550
Final R indexes [all data]	R_1 = 0.0696, wR_2 = 0.1682
Largest diff. peak/hole / e Å^{-3}	0.22/-0.33

CCDC Deposition Number: 2372085

nicotinamide: 3,5-dinitrobenzoic acid: MeOH (2:2:2)

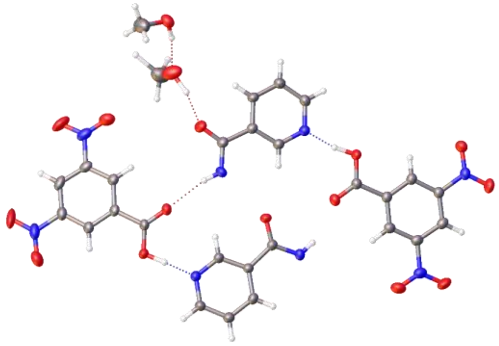

Empirical formula	$C_{14}H_{14}N_4O_8$
Formula weight	366.29
Temperature/K	150.01(10)
Crystal system	monoclinic
Space group	$P2_1/c$
a/Å	7.0215(3)
b/Å	22.3185(8)
c/Å	20.2612(7)
α/°	90
β/°	92.304(3)
γ/°	90
Volume/Å^3	3172.6(2)
Z	8
ρ_{calc}g/cm^3	1.534
μ/mm^{-1}	1.107
F(000)	1520.0
Crystal size/mm^3	0.28 × 0.12 × 0.084
Radiation	Cu Kα (λ = 1.54184)
2Θ range for data collection/°	5.894 to 156.554
Index ranges	-8 ≤ h ≤ 8, -27 ≤ k ≤ 17, -25 ≤ l ≤ 25
Reflections collected	23820
Independent reflections	6309 [R_{int} = 0.0301, R_{sigma} = 0.0262]
Data/restraints/parameters	6309/343/496
Goodness-of-fit on F^2	1.074
Final R indexes [I>=2σ (I)]	R_1 = 0.0459, wR_2 = 0.1307
Final R indexes [all data]	R_1 = 0.0577, wR_2 = 0.1427
Largest diff. peak/hole / e Å^{-3}	0.24/-0.40

CCDC Deposition Number: 2372086

nicotinamide: methyl gallate (1:1)

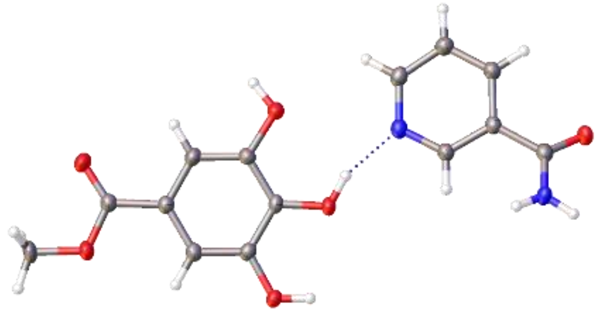

Empirical formula	$C_{14}H_{14}N_2O_6$
Formula weight	306.27
Temperature/K	149.99(10)
Crystal system	triclinic
Space group	P-1
a/Å	5.1980(3)
b/Å	7.7142(4)
c/Å	16.8659(8)
α/°	85.429(4)
β/°	88.957(4)
γ/°	83.901(4)
Volume/Å^3	670.30(6)
Z	2
ρ_{calc}g/cm^3	1.517
μ/mm^{-1}	1.026
F(000)	320.0
Crystal size/mm^3	0.39 × 0.058 × 0.042
Radiation	Cu Kα (λ = 1.54184)
2Θ range for data collection/°	5.256 to 153.828
Index ranges	-5 ≤ h ≤ 6, -9 ≤ k ≤ 9, -19 ≤ l ≤ 20
Reflections collected	6082
Independent reflections	2606 [R_{int} = 0.0371, R_{sigma} = 0.0469]
Data/restraints/parameters	2606/153/215
Goodness-of-fit on F^2	1.050
Final R indexes [I>=2σ (I)]	R_1 = 0.0449, wR_2 = 0.1245
Final R indexes [all data]	R_1 = 0.0537, wR_2 = 0.1323
Largest diff. peak/hole / e Å^{-3}	0.35/-0.26

CCDC Deposition Number: 2372087

nicotinamide: quinol (2:0.5)

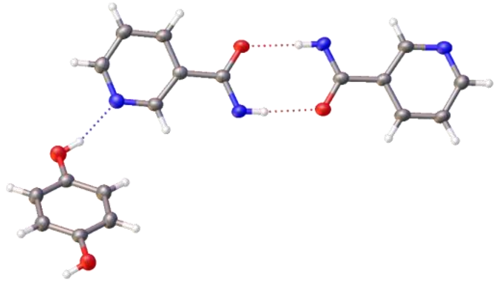

Empirical formula	$C_{15}H_{15}N_4O_3$
Formula weight	299.31
Temperature/K	150.01(10)
Crystal system	monoclinic
Space group	$P2_1/n$
a/Å	5.2125(2)
b/Å	27.0087(10)
c/Å	10.3421(3)
α/°	90
β/°	102.312(3)
γ/°	90
Volume/Å^3	1422.50(9)
Z	4
ρ_{calc}g/cm^3	1.398
μ/mm^{-1}	0.833
F(000)	628.0
Crystal size/mm^3	0.19 × 0.05 × 0.031
Radiation	Cu Kα (λ = 1.54184)
2Θ range for data collection/°	6.546 to 155.522
Index ranges	-4 ≤ h ≤ 6, -32 ≤ k ≤ 31, -13 ≤ l ≤ 12
Reflections collected	9492
Independent reflections	2788 [R_{int} = 0.0371, R_{sigma} = 0.0357]
Data/restraints/parameters	2788/135/214
Goodness-of-fit on F^2	1.036
Final R indexes [I>=2σ (I)]	R_1 = 0.0553, wR_2 = 0.1485
Final R indexes [all data]	R_1 = 0.0648, wR_2 = 0.1551
Largest diff. peak/hole / e Å^{-3}	0.36/-0.35

S5.3 Previously Known Binary Co-Crystals from Ternary and Quaternary Co-Crystal Screening

CCDC Deposition Number: 2372088

4,4'-bipyridine_3,3'-thiodipropinoic acid (0.5:0.5)

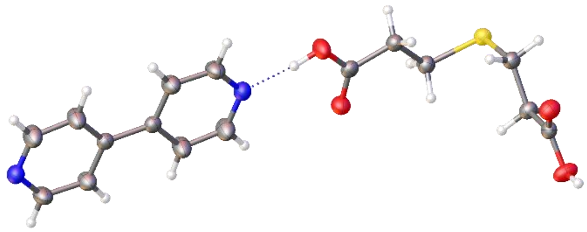

Empirical formula	$C_{16}H_{18}N_2O_4S$
Formula weight	334.38
Temperature/K	150.00(10)
Crystal system	monoclinic
Space group	P2/n
a/Å	11.3891(4)
b/Å	4.84020(10)
c/Å	15.7088(5)
α/°	90
β/°	110.000(4)
γ/°	90
Volume/Å^3	813.73(5)
Z	2
ρ_{calc}g/cm^3	1.365
μ/mm^{-1}	1.962
F(000)	352.0
Crystal size/mm^3	0.32 × 0.073 × 0.056
Radiation	Cu Kα (λ = 1.54184)
2Θ range for data collection/°	8.384 to 151.494
Index ranges	-13 ≤ h ≤ 14, -4 ≤ k ≤ 5, -18 ≤ l ≤ 19
Reflections collected	4321
Independent reflections	1577 [R_{int} = 0.0323, R_{sigma} = 0.0343]
Data/restraints/parameters	1577/64/108
Goodness-of-fit on F^2	1.071
Final R indexes [I>=2σ (I)]	R_1 = 0.0353, wR_2 = 0.0911
Final R indexes [all data]	R_1 = 0.0397, wR_2 = 0.0943
Largest diff. peak/hole / e Å^{-3}	0.17/-0.22

CCDC Deposition Number: 2372089

Quinol: tetramethylpyrazine (0.5:0.5)

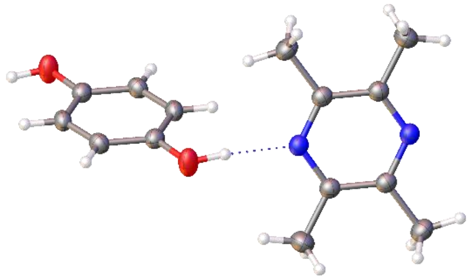

Empirical formula	$C_{14}H_{18}N_2O_2$
Formula weight	246.30
Temperature/K	149.99(10)
Crystal system	monoclinic
Space group	$P2_1/c$
a/Å	7.6282(4)
b/Å	9.2277(4)
c/Å	10.0237(5)
α/°	90
β/°	111.011(6)
γ/°	90
Volume/Å^3	658.66(6)
Z	2
ρ_{calc}g/cm^3	1.242
μ/mm^{-1}	0.675
F(000)	264.0
Crystal size/mm^3	0.32 × 0.15 × 0.049
Radiation	Cu Kα (λ = 1.54184)
2Θ range for data collection/°	12.43 to 154.812
Index ranges	$-9 \le h \le 9, -10 \le k \le 7, -12 \le l \le 11$
Reflections collected	3804
Independent reflections	1299 [R_{int} = 0.0195, R_{sigma} = 0.0199]
Data/restraints/parameters	1299/36/87
Goodness-of-fit on F^2	1.075
Final R indexes [I>=2σ (I)]	R_1 = 0.0338, wR_2 = 0.0906
Final R indexes [all data]	R_1 = 0.0363, wR_2 = 0.0922
Largest diff. peak/hole / e Å^{-3}	0.14/-0.17

CCDC Deposition Number: 2372090

Caffeine: oxalic acid (1:0.5)

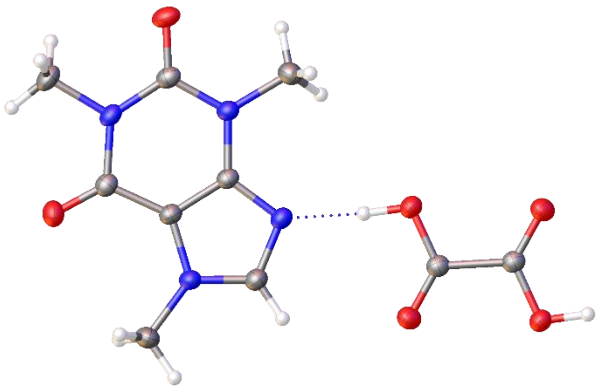

Empirical formula	$C_9H_{11}N_4O_4$
Formula weight	239.22
Temperature/K	150.00(10)
Crystal system	monoclinic
Space group	$P2_1/c$
a/Å	4.40790(10)
b/Å	14.7459(4)
c/Å	15.8719(4)
α/°	90
β/°	96.239(2)
γ/°	90
Volume/Å^3	1025.54(4)
Z	4
ρ_{calc}g/cm^3	1.549
μ/mm^{-1}	1.063
F(000)	500.0
Crystal size/mm^3	0.15 × 0.038 × 0.028
Radiation	Cu Kα (λ = 1.54184)
2Θ range for data collection/°	8.208 to 152.292
Index ranges	-3 ≤ h ≤ 5, -17 ≤ k ≤ 17, -18 ≤ l ≤ 19
Reflections collected	6546
Independent reflections	2054 [R_{int} = 0.0244, R_{sigma} = 0.0257]
Data/restraints/parameters	2054/123/160
Goodness-of-fit on F^2	1.042
Final R indexes [I>=2σ (I)]	R_1 = 0.0346, wR_2 = 0.0909
Final R indexes [all data]	R_1 = 0.0414, wR_2 = 0.0952
Largest diff. peak/hole / e Å^{-3}	0.21/-0.24

CCDC Deposition Number: 2372091

4,4'-bipyridine: orcinol (1.5:1)

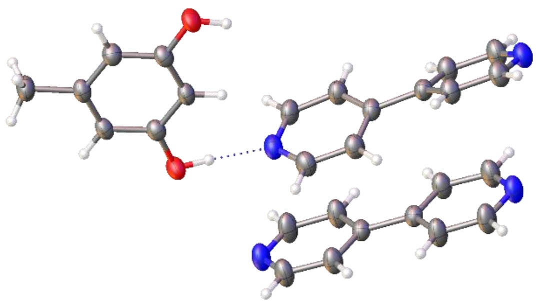

Empirical formula	$C_{44}H_{40}N_6O_4$
Formula weight	716.82
Temperature/K	150.10(10)
Crystal system	triclinic
Space group	P-1
a/Å	8.7799(8)
b/Å	10.0344(10)
c/Å	11.9985(10)
α/°	67.885(9)
β/°	77.744(7)
γ/°	69.420(9)
Volume/Å^3	912.91(16)
Z	1
ρ_{calc}g/cm^3	1.304
μ/mm^{-1}	0.683
F(000)	378.0
Crystal size/mm^3	0.27 × 0.23 × 0.15
Radiation	Cu Kα (λ = 1.54184)
2Θ range for data collection/°	7.988 to 149.034
Index ranges	-10 ≤ h ≤ 10, -12 ≤ k ≤ 12, -14 ≤ l ≤ 14
Reflections collected	8435
Independent reflections	3491 [R_{int} = 0.0253, R_{sigma} = 0.0326]
Data/restraints/parameters	3491/186/251
Goodness-of-fit on F^2	1.076
Final R indexes [I>=2σ (I)]	R_1 = 0.0517, wR_2 = 0.1416
Final R indexes [all data]	R_1 = 0.0576, wR_2 = 0.1476
Largest diff. peak/hole / e Å^{-3}	0.39/-0.26

S5.4 New Binary Co-Crystals from Ternary and Quaternary Co-Crystal Screening

CCDC Deposition Number: 2372092

orcinol: phenazine: 1,4-dioxane (1:1:0.5)

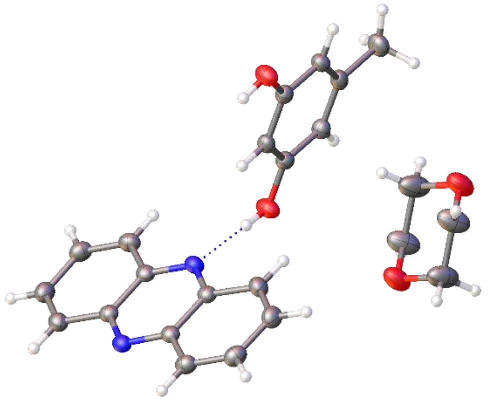

Empirical formula	$C_{21}H_{20}N_2O_3$
Formula weight	348.39
Temperature/K	150.11(10)
Crystal system	triclinic
Space group	P-1
a/Å	7.4821(4)
b/Å	9.4572(4)
c/Å	13.6078(7)
α/°	83.084(4)
β/°	78.243(5)
γ/°	68.600(5)
Volume/Å^3	876.57(8)
Z	2
ρ_{calc}g/cm^3	1.320
μ/mm^{-1}	0.721
F(000)	368.0
Crystal size/mm^3	0.21 × 0.16 × 0.067
Radiation	Cu Kα (λ = 1.54184)
2Θ range for data collection/°	6.644 to 148.942
Index ranges	$-9 \le h \le 6$, $-11 \le k \le 11$, $-16 \le l \le 16$
Reflections collected	8381
Independent reflections	3365 [R_{int} = 0.0228, R_{sigma} = 0.0288]
Data/restraints/parameters	3365/186/242
Goodness-of-fit on F^2	1.072
Final R indexes [I>=2σ (I)]	R_1 = 0.0464, wR_2 = 0.1318
Final R indexes [all data]	R_1 = 0.0526, wR_2 = 0.1376
Largest diff. peak/hole / e Å^{-3}	0.32/-0.25

CCDC Deposition Number: 2372093

nicotinamide quinol (2:0.5)

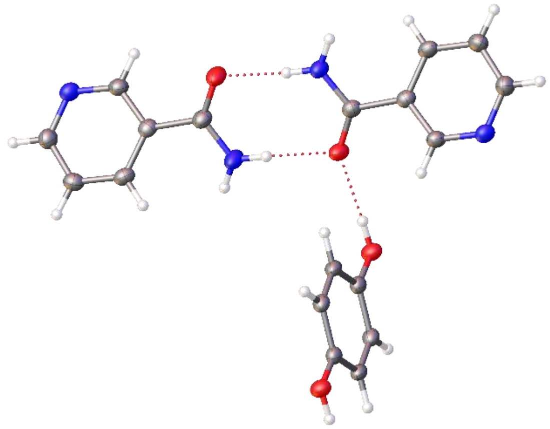

Empirical formula	$C_{15}H_{15}N_4O_3$
Formula weight	299.31
Temperature/K	149.99(10)
Crystal system	monoclinic
Space group	$P2_1/c$
a/Å	7.24400(10)
b/Å	11.3576(2)
c/Å	17.2682(3)
α/°	90
β/°	97.939(2)
γ/°	90
Volume/Å^3	1407.11(4)
Z	4
ρ_{calc}g/cm^3	1.413
μ/mm^{-1}	0.842
F(000)	628.0
Crystal size/mm^3	0.33 × 0.08 × 0.03
Radiation	Cu Kα (λ = 1.54184)
2Θ range for data collection/°	9.348 to 154.388
Index ranges	-8 ≤ h ≤ 5, -14 ≤ k ≤ 13, -21 ≤ l ≤ 21
Reflections collected	14707
Independent reflections	2841 [R_{int} = 0.0294, R_{sigma} = 0.0231]
Data/restraints/parameters	2841/138/214
Goodness-of-fit on F^2	1.043
Final R indexes [I>=2σ (I)]	R_1 = 0.0336, wR_2 = 0.0908
Final R indexes [all data]	R_1 = 0.0374, wR_2 = 0.0932
Largest diff. peak/hole / e Å^{-3}	0.21/-0.20

CCDC Deposition Number: 2372094

3-hydroxy-2-naphthoic acid: 1,2-bis(4-pyridyl)ethane (1:0.5)

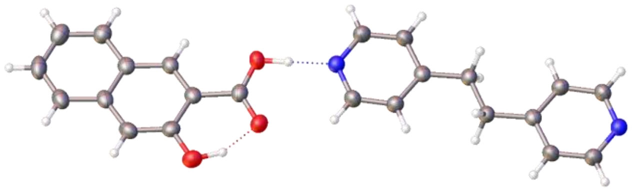

Empirical formula	$C_{17}H_{14}NO_3$
Formula weight	280.29
Temperature/K	149.99(10)
Crystal system	monoclinic
Space group	$P2_1/c$
a/Å	10.6221(4)
b/Å	11.3226(3)
c/Å	11.5995(5)
α/°	90
β/°	107.861(4)
γ/°	90
Volume/Å^3	1327.83(9)
Z	4
ρ_{calc}g/cm^3	1.402
μ/mm^{-1}	0.790
F(000)	588.0
Crystal size/mm^3	0.15 × 0.1 × 0.077
Radiation	Cu Kα (λ = 1.54184)
2Θ range for data collection/°	11.194 to 152.754
Index ranges	-12 ≤ h ≤ 13, -13 ≤ k ≤ 13, -13 ≤ l ≤ 13
Reflections collected	13885
Independent reflections	2640 [R_{int} = 0.0350, R_{sigma} = 0.0247]
Data/restraints/parameters	2640/154/196
Goodness-of-fit on F^2	1.159
Final R indexes [I>=2σ (I)]	R_1 = 0.0532, wR_2 = 0.1249
Final R indexes [all data]	R_1 = 0.0623, wR_2 = 0.1294
Largest diff. peak/hole / e Å^{-3}	0.25/-0.19

CCDC Deposition Number: 2372095

methyl gallate: [2,2′-bipyridine]-4,4-diyldimethanol (1:0.5)

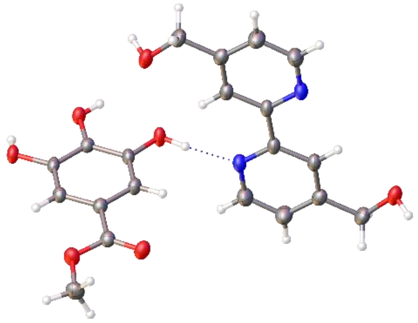

Empirical formula	$C_{28}H_{28}N_2O_{12}$
Formula weight	584.52
Temperature/K	149.99(10)
Crystal system	monoclinic
Space group	C2/c
a/Å	16.4668(6)
b/Å	4.6038(2)
c/Å	34.3069(11)
α/°	90
β/°	93.870(4)
γ/°	90
Volume/Å^3	2594.87(17)
Z	4
ρ_{calc}g/cm^3	1.496
μ/mm^{-1}	1.007
F(000)	1224.0
Crystal size/mm^3	0.33 × 0.044 × 0.012
Radiation	Cu Kα (λ = 1.54184)
2Θ range for data collection/°	5.164 to 152.61
Index ranges	-20 ≤ h ≤ 20, -5 ≤ k ≤ 5, -26 ≤ l ≤ 40
Reflections collected	14640
Independent reflections	2624 [R_{int} = 0.0602, R_{sigma} = 0.0456]
Data/restraints/parameters	2624/144/203
Goodness-of-fit on F^2	1.062
Final R indexes [I>=2σ (I)]	R_1 = 0.0455, wR_2 = 0.1115
Final R indexes [all data]	R_1 = 0.0613, wR_2 = 0.1207
Largest diff. peak/hole / e Å^{-3}	0.17/-0.22

CCDC Deposition Number: 2372096

3,5-dinitrobenzoic acid: orcinol: H_2O (4:4:4)

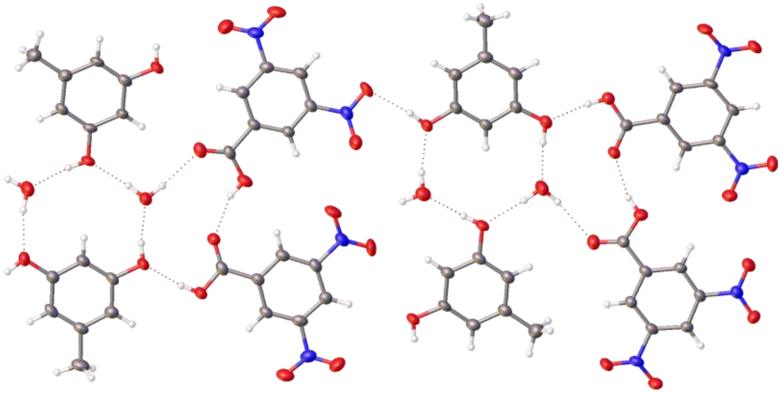

Empirical formula	$C_{14}H_{14}N_2O_9$
Formula weight	354.27
Temperature/K	150.15
Crystal system	triclinic
Space group	P1
a/Å	7.9379(2)
b/Å	14.1423(4)
c/Å	14.6111(3)
α/°	74.495(2)
β/°	76.612(2)
γ/°	79.312(2)
Volume/Å^3	1524.18(7)
Z	4
ρ_{calc}g/cm^3	1.544
μ/mm^{-1}	1.142
F(000)	736.0
Crystal size/mm^3	0.08 × 0.038 × 0.028
Radiation	CuKα (λ = 1.54184)
2Θ range for data collection/°	6.396 to 153.01
Index ranges	-10 ≤ h ≤ 9, -17 ≤ k ≤ 17, -10 ≤ l ≤ 17
Reflections collected	16067
Independent reflections	7971 [R_{int} = 0.0291, R_{sigma} = 0.0370]
Data/restraints/parameters	7971/687/929
Goodness-of-fit on F^2	1.042
Final R indexes [I>=2σ (I)]	R_1 = 0.0413, wR_2 = 0.1117
Final R indexes [all data]	R_1 = 0.0469, wR_2 = 0.1164
Largest diff. peak/hole / e Å^{-3}	0.24/-0.27
Flack parameter	0.36(18)

CCDC Deposition Number: 2372097

4,4'-bipyridine: propyl gallate (1:1)

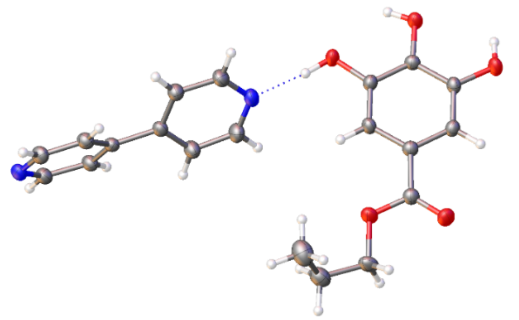

Empirical formula	$C_{20}H_{20}N_2O_5$
Formula weight	368.38
Temperature/K	149.99(10)
Crystal system	monoclinic
Space group	$P2_1/n$
a/Å	7.39890(10)
b/Å	15.08310(10)
c/Å	16.3866(2)
α/°	90
β/°	91.8720(10)
γ/°	90
Volume/Å^3	1827.74(4)
Z	4
ρ_{calc}g/cm^3	1.339
μ/mm^{-1}	0.805
F(000)	776.0
Crystal size/mm^3	0.15 × 0.078 × 0.046
Radiation	Cu Kα (λ = 1.54184)
2Θ range for data collection/°	7.968 to 154.042
Index ranges	$-7 \le h \le 9$, $-19 \le k \le 18$, $-20 \le l \le 20$
Reflections collected	18782
Independent reflections	3678 [R_{int} = 0.0193, R_{sigma} = 0.0161]
Data/restraints/parameters	3678/192/254
Goodness-of-fit on F^2	1.045
Final R indexes [I>=2σ (I)]	R_1 = 0.0355, wR_2 = 0.0972
Final R indexes [all data]	R_1 = 0.0382, wR_2 = 0.0993
Largest diff. peak/hole / e Å^{-3}	0.22/-0.32

CCDC Deposition Number: 2428848

4,4'-bipyridine: 2-bromoresorcinol (3:2)

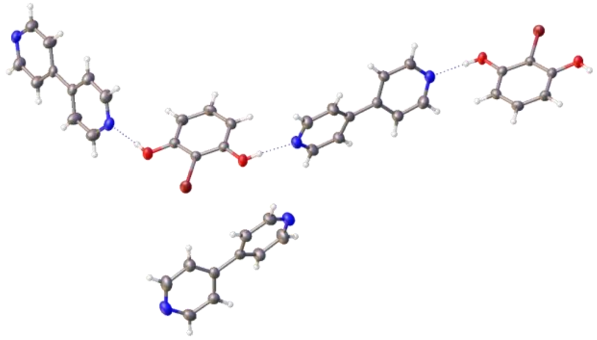

Empirical formula	$C_{42}H_{34}Br_2N_6O_4$
Formula weight	846.57
Temperature/K	150.00(10)
Crystal system	triclinic
Space group	P-1
a/Å	7.65110(10)
b/Å	15.0607(3)
c/Å	18.0470(4)
α/°	111.013(2)
β/°	90.607(2)
γ/°	104.172(2)
Volume/Å^3	1871.04(7)
Z	2
ρ_{calc}g/cm^3	1.503
μ/mm^{-1}	3.170
F(000)	860.0
Crystal size/mm^3	0.13 × 0.08 × 0.02
Radiation	Cu Kα (λ = 1.54184)
2Θ range for data collection/°	5.276 to 154.882
Index ranges	-9 ≤ h ≤ 6, -18 ≤ k ≤ 19, -22 ≤ l ≤ 22
Reflections collected	23062
Independent reflections	7415 [R_{int} = 0.0359, R_{sigma} = 0.0362]
Data/restraints/parameters	7415/381/491
Goodness-of-fit on F^2	1.051
Final R indexes [I>=2σ (I)]	R_1 = 0.0344, wR_2 = 0.0865
Final R indexes [all data]	R_1 = 0.0455, wR_2 = 0.0924
Largest diff. peak/hole / e Å^{-3}	0.30/-0.50

S5.5 Previously Known Ternary Co-Crystals from Ternary Co-Crystal Screening

CCDC Deposition Number: 2372098

toluic acid: isonicotinamide: 3,5-dinitrobenzoic acid (1:1:1)

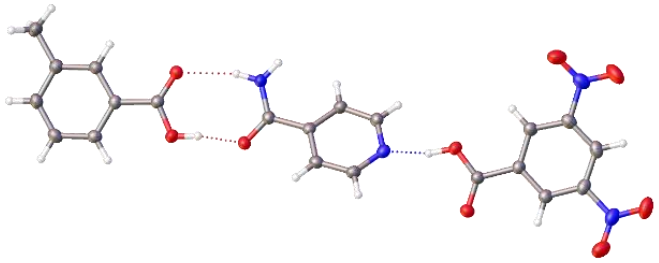

Empirical formula	$C_{21}H_{18}N_4O_9$
Formula weight	470.39
Temperature/K	150.15
Crystal system	triclinic
Space group	P-1
a/Å	7.0561(3)
b/Å	8.7283(3)
c/Å	16.8685(4)
α/°	94.314(2)
β/°	91.082(3)
γ/°	95.194(3)
Volume/Å^3	1031.35(6)
Z	2
ρ_{calc}g/cm^3	1.515
μ/mm^{-1}	1.033
F(000)	488.0
Crystal size/mm^3	0.15 × 0.11 × 0.02
Radiation	Cu Kα (λ = 1.54184)
2Θ range for data collection/°	5.256 to 147.182
Index ranges	$-8 \le h \le 8$, $-10 \le k \le 9$, $-20 \le l \le 20$
Reflections collected	8850
Independent reflections	3959 [R_{int} = 0.0205, R_{sigma} = 0.0278]
Data/restraints/parameters	3959/238/320
Goodness-of-fit on F^2	1.042
Final R indexes [I>=2σ (I)]	R_1 = 0.0430, wR_2 = 0.1183
Final R indexes [all data]	R_1 = 0.0473, wR_2 = 0.1217
Largest diff. peak/hole / e Å^{-3}	0.22/-0.40

CCDC Deposition Number: 2372099

4,4'-bipyridine: orcinol: phenazine (1:1:0.5)

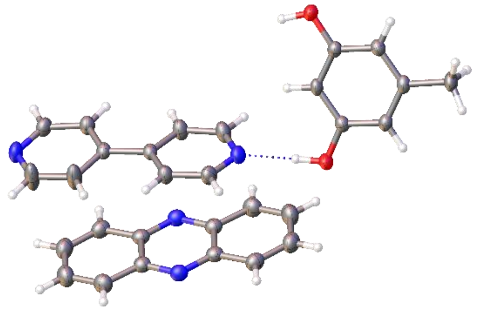

Empirical formula	$C_{46}H_{40}N_6O_4$
Formula weight	740.84
Temperature/K	149.97(10)
Crystal system	triclinic
Space group	P-1
a/Å	9.3979(3)
b/Å	10.2114(3)
c/Å	11.1495(4)
α/°	69.252(3)
β/°	86.066(3)
γ/°	69.495(3)
Volume/Å^3	935.30(6)
Z	1
ρ_{calc}g/cm^3	1.315
μ/mm^{-1}	0.686
F(000)	390.0
Crystal size/mm^3	0.14 × 0.088 × 0.068
Radiation	Cu Kα (λ = 1.54184)
2Θ range for data collection/°	8.498 to 148.792
Index ranges	-11 ≤ h ≤ 11, -12 ≤ k ≤ 11, -13 ≤ l ≤ 13
Reflections collected	8840
Independent reflections	3616 [R_{int} = 0.0233, R_{sigma} = 0.0280]
Data/restraints/parameters	3616/183/260
Goodness-of-fit on F^2	1.061
Final R indexes [I>=2σ (I)]	R_1 = 0.0410, wR_2 = 0.1104
Final R indexes [all data]	R_1 = 0.0475, wR_2 = 0.1156
Largest diff. peak/hole / e Å^{-3}	0.16/-0.28

CCDC Deposition Number: 2372100

nicotinamide: fumaric acid: isoniazid (1:1:1)

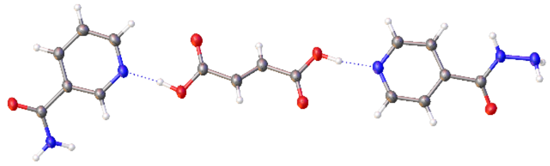

Empirical formula	$C_{16}H_{17}N_5O_6$
Formula weight	375.34
Temperature/K	150
Crystal system	monoclinic
Space group	$P2_1/n$
a/Å	5.0709(2)
b/Å	17.2524(7)
c/Å	19.1542(6)
α/°	90
β/°	97.574(3)
γ/°	90
Volume/Å^3	1661.09(11)
Z	4
ρ_{calc}g/cm^3	1.501
μ/mm^{-1}	0.997
F(000)	784.0
Crystal size/mm^3	0.17 × 0.13 × 0.038
Radiation	Cu Kα (λ = 1.54184)
2Θ range for data collection/°	6.922 to 154.918
Index ranges	-4 ≤ h ≤ 6, -19 ≤ k ≤ 21, -22 ≤ l ≤ 24
Reflections collected	10559
Independent reflections	3289 [R_{int} = 0.0399, R_{sigma} = 0.0445]
Data/restraints/parameters	3289/173/265
Goodness-of-fit on F^2	1.023
Final R indexes [I>=2σ (I)]	R_1 = 0.0419, wR_2 = 0.1014
Final R indexes [all data]	R_1 = 0.0612, wR_2 = 0.1115
Largest diff. peak/hole / e Å^{-3}	0.23/-0.23

CCDC Deposition Number: 2372101

tetramethylpyrazine: 2,2'-bipyridine: 2-chlororesorcinol (1:0.5:0.5)

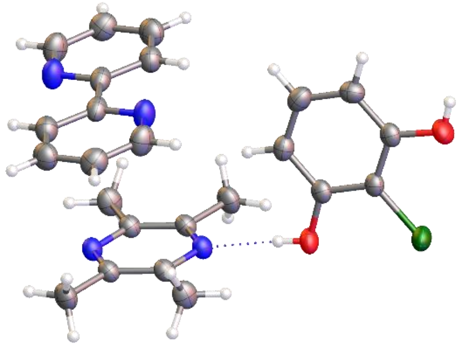

Empirical formula	$C_{30}H_{30}Cl_2N_4O_4$
Formula weight	581.48
Temperature/K	149.99(10)
Crystal system	monoclinic
Space group	$P2_1/n$
a/Å	7.8520(3)
b/Å	20.7471(8)
c/Å	8.5304(4)
α/°	90
β/°	90.078(4)
γ/°	90
Volume/Å^3	1389.65(10)
Z	2
ρ_{calc}g/cm^3	1.390
μ/mm^{-1}	2.462
F(000)	608.0
Crystal size/mm^3	0.099 × 0.082 × 0.021
Radiation	Cu Kα (λ = 1.54184)
2Θ range for data collection/°	8.524 to 153.604
Index ranges	-8 ≤ h ≤ 9, -26 ≤ k ≤ 24, -10 ≤ l ≤ 10
Reflections collected	17283
Independent reflections	2823 [R_{int} = 0.0440, R_{sigma} = 0.0288]
Data/restraints/parameters	2823/117/189
Goodness-of-fit on F^2	1.051
Final R indexes [I>=2σ (I)]	R_1 = 0.0401, wR_2 = 0.1066
Final R indexes [all data]	R_1 = 0.0501, wR_2 = 0.1123
Largest diff. peak/hole / e Å^{-3}	0.34/-0.42

S5.6 New Ternary Co-Crystals from Ternary Co-Crystal Screening

CCDC Deposition Number: 2372102

tetramethylpyrazine: 2,2'-bipyridine: 2-chlororesorcinol (1:0.5:1)

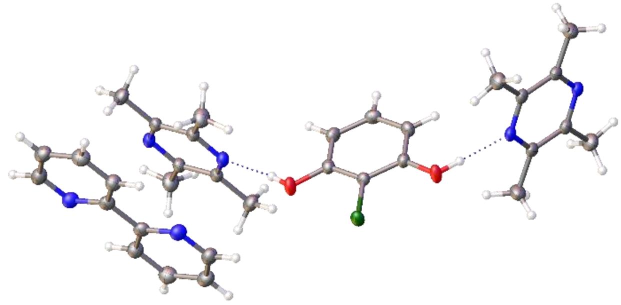

Empirical formula	$C_{38}H_{42}Cl_2N_6O_4$
Formula weight	717.67
Temperature/K	150.00(10)
Crystal system	triclinic
Space group	P-1
a/Å	7.3947(3)
b/Å	8.0144(3)
c/Å	15.5176(5)
α/°	98.267(3)
β/°	90.707(3)
γ/°	100.188(3)
Volume/Å^3	895.06(6)
Z	1
ρ_{calc}g/cm^3	1.331
μ/mm^{-1}	2.031
F(000)	378.0
Crystal size/mm^3	0.18 × 0.051 × 0.011
Radiation	Cu Kα (λ = 1.54184)
2Θ range for data collection/°	5.76 to 153.244
Index ranges	-9 ≤ h ≤ 9, -10 ≤ k ≤ 9, -11 ≤ l ≤ 18
Reflections collected	7798
Independent reflections	3502 [R_{int} = 0.0302, R_{sigma} = 0.0403]
Data/restraints/parameters	3502/138/236
Goodness-of-fit on F^2	1.042
Final R indexes [I>=2σ (I)]	R_1 = 0.0357, wR_2 = 0.0907
Final R indexes [all data]	R_1 = 0.0458, wR_2 = 0.0952
Largest diff. peak/hole / e Å^{-3}	0.31/-0.23

CCDC Deposition Number: 2372103

4,4’-bipyridine: methyl gallate: 2-chlororesorcinol: H_2O (3:2:1:2)

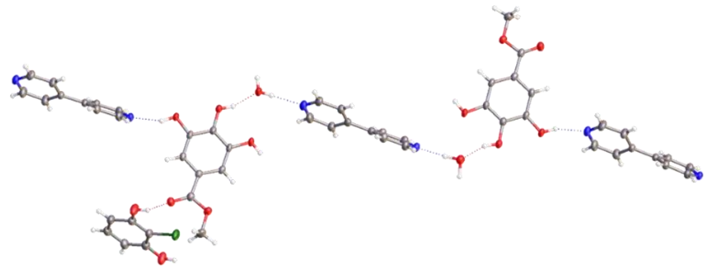

Empirical formula	$C_{52}H_{49}ClN_6O_{14}$
Formula weight	1017.42
Temperature/K	150.00(10)
Crystal system	monoclinic
Space group	Cc
a/Å	9.72670(10)
b/Å	16.8039(2)
c/Å	29.2741(3)
α/°	90
β/°	95.9490(10)
γ/°	90
Volume/Å^3	4758.98(9)
Z	4
ρ_{calc}g/cm^3	1.420
μ/mm^{-1}	1.365
F(000)	2128.0
Crystal size/mm^3	0.22 × 0.19 × 0.045
Radiation	Cu Kα (λ = 1.54184)
2Θ range for data collection/°	6.07 to 149.124
Index ranges	-11 ≤ h ≤ 12, -20 ≤ k ≤ 20, -35 ≤ l ≤ 34
Reflections collected	35270
Independent reflections	8034 [R_{int} = 0.0251, R_{sigma} = 0.0181]
Data/restraints/parameters	8034/516/708
Goodness-of-fit on F^2	1.060
Final R indexes [I>=2σ (I)]	R_1 = 0.0262, wR_2 = 0.0730
Final R indexes [all data]	R_1 = 0.0268, wR_2 = 0.0733
Largest diff. peak/hole / e Å^{-3}	0.14/-0.25
Flack parameter	0.009(4)

CCDC Deposition Number: 2372104

caffeine: 3,5-dinitrobenzoic acid: 2-methylresorcinol: H_2O (1:1:2:1)

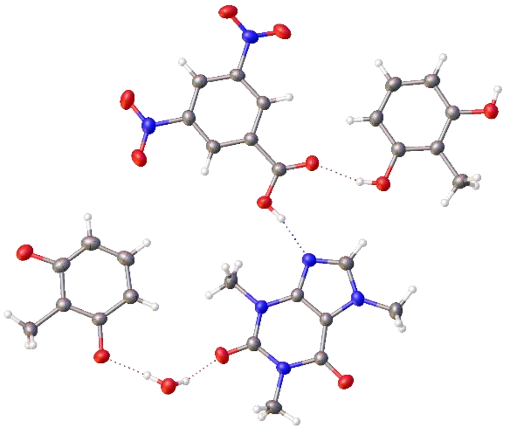

Empirical formula	$C_{29}H_{32}N_6O_{13}$
Formula weight	672.60
Temperature/K	150.0(1)
Crystal system	triclinic
Space group	P-1
a/Å	9.58200(10)
b/Å	12.3827(2)
c/Å	13.8822(2)
α/°	97.9610(10)
β/°	101.5130(10)
γ/°	104.9690(10)
Volume/Å^3	1527.48(4)
Z	2
ρ_{calc}g/cm^3	1.462
μ/mm^{-1}	0.997
F(000)	704.0
Crystal size/mm^3	0.22 × 0.16 × 0.025
Radiation	Cu Kα (λ = 1.54184)
2Θ range for data collection/°	6.632 to 153.076
Index ranges	-11 ≤ h ≤ 11, -15 ≤ k ≤ 15, -15 ≤ l ≤ 17
Reflections collected	29437
Independent reflections	5988 [R_{int} = 0.0239, R_{sigma} = 0.0191]
Data/restraints/parameters	5988/414/517
Goodness-of-fit on F^2	1.039
Final R indexes [I>=2σ (I)]	R_1 = 0.0411, wR_2 = 0.1164
Final R indexes [all data]	R_1 = 0.0465, wR_2 = 0.1207
Largest diff. peak/hole / e Å^{-3}	0.51/-0.23

CCDC Deposition Number: 2372105

nicotinamide: 3,5-dinitrobenzoic acid: glutaric acid (1:1:1)

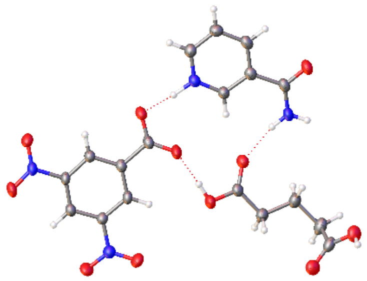

Empirical formula	$C_{18}H_{18}N_4O_{11}$
Formula weight	466.36
Temperature/K	149.99(10)
Crystal system	triclinic
Space group	P-1
a/Å	8.9531(3)
b/Å	10.8195(5)
c/Å	11.2748(4)
α/°	77.198(4)
β/°	69.904(4)
γ/°	84.497(4)
Volume/Å^3	999.95(7)
Z	2
ρ_{calc}g/cm^3	1.549
μ/mm^{-1}	1.133
F(000)	484.0
Crystal size/mm^3	0.16 × 0.064 × 0.035
Radiation	Cu Kα (λ = 1.54184)
2Θ range for data collection/°	8.382 to 154.036
Index ranges	$-11 \le h \le 9$, $-12 \le k \le 13$, $-13 \le l \le 14$
Reflections collected	9860
Independent reflections	3918 [R_{int} = 0.0405, R_{sigma} = 0.0487]
Data/restraints/parameters	3918/221/314
Goodness-of-fit on F^2	1.036
Final R indexes [I>=2σ (I)]	R_1 = 0.0498, wR_2 = 0.1263
Final R indexes [all data]	R_1 = 0.0667, wR_2 = 0.1352
Largest diff. peak/hole / e Å^{-3}	0.22/-0.31

CCDC Deposition Number: 2372106

nicotinamide: 3,5-dinitrobenzoic acid: tetramethylpyrazine: H_2O (1:1:0.5:1)

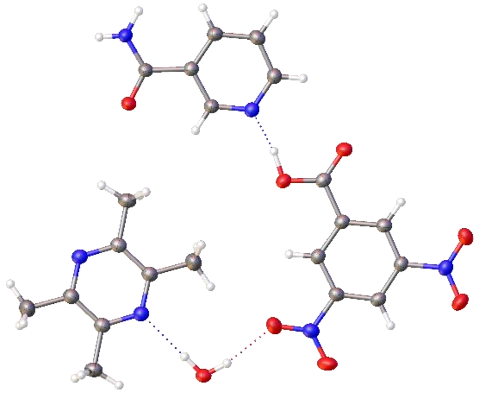

Empirical formula	$C_{17}H_{18}N_5O_8$
Formula weight	420.36
Temperature/K	149.99(10)
Crystal system	triclinic
Space group	P-1
a/Å	8.3854(3)
b/Å	9.0036(3)
c/Å	14.0849(4)
α/°	71.684(3)
β/°	88.310(3)
γ/°	70.014(3)
Volume/Å^3	945.10(6)
Z	2
ρ_{calc}g/cm^3	1.477
μ/mm^{-1}	1.024
F(000)	438.0
Crystal size/mm^3	0.39 × 0.1 × 0.07
Radiation	Cu Kα (λ = 1.54184)
2Θ range for data collection/°	6.636 to 154.352
Index ranges	-10 ≤ h ≤ 9, -11 ≤ k ≤ 11, -17 ≤ l ≤ 13
Reflections collected	9132
Independent reflections	3703 [R_{int} = 0.0281, R_{sigma} = 0.0351]
Data/restraints/parameters	3703/190/288
Goodness-of-fit on F^2	1.033
Final R indexes [I>=2σ (I)]	R_1 = 0.0389, wR_2 = 0.1054
Final R indexes [all data]	R_1 = 0.0447, wR_2 = 0.1106
Largest diff. peak/hole / e Å^{-3}	0.20/-0.35

CCDC Deposition Number: 2372107

nicotinamide: quinol: benzoic acid (1:0.5:1)

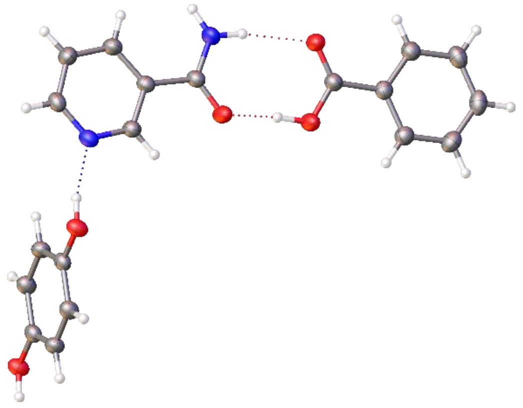

Empirical formula	$C_{16}H_{15}N_2O_4$
Formula weight	299.30
Temperature/K	150.01(10)
Crystal system	triclinic
Space group	P-1
a/Å	6.2129(2)
b/Å	7.0141(2)
c/Å	17.4372(4)
α/°	92.402(2)
β/°	100.028(2)
γ/°	106.882(3)
Volume/Å^3	712.56(4)
Z	2
ρ_{calc}g/cm^3	1.395
μ/mm^{-1}	0.845
F(000)	314.0
Crystal size/mm^3	0.3 × 0.17 × 0.039
Radiation	Cu Kα (λ = 1.54184)
2Θ range for data collection/°	5.172 to 133.2
Index ranges	$-6 \le h \le 7$, $-8 \le k \le 8$, $-20 \le l \le 20$
Reflections collected	5883
Independent reflections	2512 [R_{int} = 0.0240, R_{sigma} = 0.0314]
Data/restraints/parameters	2512/138/211
Goodness-of-fit on F^2	1.070
Final R indexes [I>=2σ (I)]	R_1 = 0.0362, wR_2 = 0.0997
Final R indexes [all data]	R_1 = 0.0401, wR_2 = 0.1032
Largest diff. peak/hole / e Å^{-3}	0.16/-0.22

S5.7 New Ternary Co-Crystals from Quaternary Co-Crystal Screening

CCDC Deposition Number: 2372108

4,4'-bipyridine: methyl gallate: 2-chlororesorcinol (3:1:1)

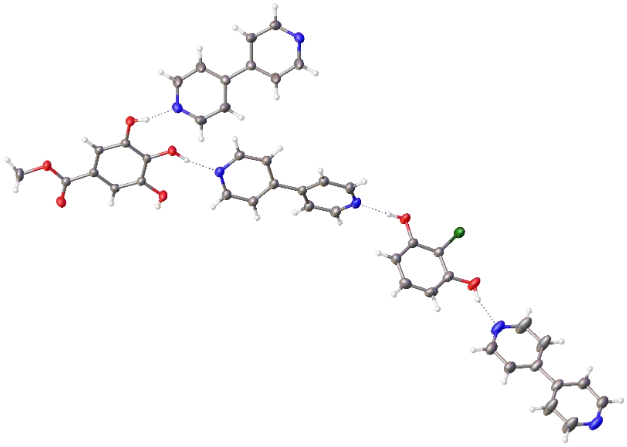

Empirical formula	$C_{34}H_{29}ClN_4O_7$
Formula weight	641.06
Temperature/K	150.00(10)
Crystal system	triclinic
Space group	P-1
a/Å	7.9386(2)
b/Å	12.6176(3)
c/Å	16.5093(4)
α/°	101.847(2)
β/°	101.908(2)
γ/°	102.993(2)
Volume/Å^3	1520.73(7)
Z	2
ρ_{calc}g/cm^3	1.400
μ/mm^{-1}	1.596
F(000)	668.0
Crystal size/mm^3	0.24 × 0.065 × 0.026
Radiation	Cu Kα (λ = 1.54184)
2Θ range for data collection/°	5.672 to 149.084
Index ranges	-9 ≤ h ≤ 9, -15 ≤ k ≤ 15, -20 ≤ l ≤ 20
Reflections collected	16736
Independent reflections	5944 [R_{int} = 0.0360, R_{sigma} = 0.0433]
Data/restraints/parameters	5944/312/431
Goodness-of-fit on F^2	1.046
Final R indexes [I>=2σ (I)]	R_1 = 0.0381, wR_2 = 0.0956
Final R indexes [all data]	R_1 = 0.0465, wR_2 = 0.1004
Largest diff. peak/hole / e Å^{-3}	0.20/-0.30

CCDC Deposition Number: 2372109

caffeine: 2-methylresorcinol: oxalic acid (1:1:0.5)

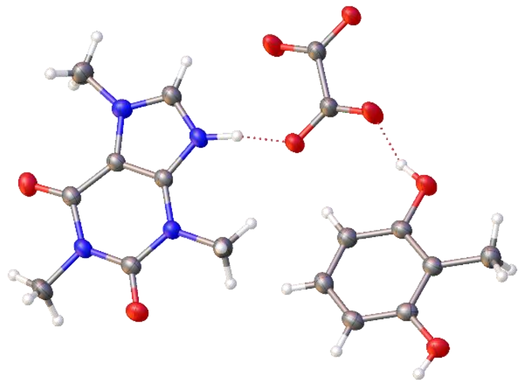

Empirical formula	$C_{16}H_{19}N_4O_6$
Formula weight	363.35
Temperature/K	149.99(10)
Crystal system	monoclinic
Space group	$P2_1/c$
a/Å	15.9751(3)
b/Å	6.70350(10)
c/Å	16.3552(3)
α/°	90
β/°	106.448(2)
γ/°	90
Volume/Å^3	1679.79(5)
Z	4
ρ_{calc}g/cm^3	1.437
μ/mm^{-1}	0.945
F(000)	764.0
Crystal size/mm^3	0.14 × 0.076 × 0.024
Radiation	Cu Kα (λ = 1.54184)
2Θ range for data collection/°	5.768 to 152.282
Index ranges	-19 ≤ h ≤ 18, -7 ≤ k ≤ 8, -20 ≤ l ≤ 19
Reflections collected	19020
Independent reflections	3294 [R_{int} = 0.0413, R_{sigma} = 0.0303]
Data/restraints/parameters	3294/186/248
Goodness-of-fit on F^2	1.041
Final R indexes [I>=2σ (I)]	R_1 = 0.0445, wR_2 = 0.1229
Final R indexes [all data]	R_1 = 0.0527, wR_2 = 0.1300
Largest diff. peak/hole / e Å^{-3}	0.29/-0.25

S5.8 Previously Known Quaternary Co-Crystals from Quaternary Co-Crystal Screening

CCDC Deposition Number: 2372110

2-chlororesorcinol: tetramethylpyrazine: 2,2'-bithiophene: 1,2-bis(4-pyridyl)ethane (1:0.5:0.5:0.5)

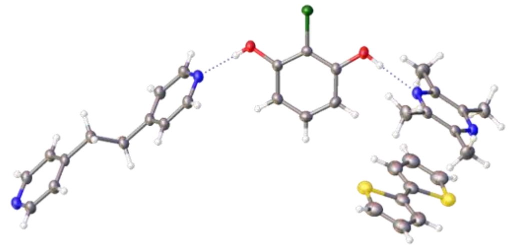

Empirical formula	$C_{40}H_{40}Cl_2N_4O_4S_2$
Formula weight	775.78
Temperature/K	149.99(10)
Crystal system	triclinic
Space group	P-1
a/Å	7.48440(10)
b/Å	8.56800(10)
c/Å	14.7525(2)
α/°	98.4620(10)
β/°	96.8260(10)
γ/°	94.1810(10)
Volume/Å^3	925.15(2)
Z	1
ρ_{calc}g/cm^3	1.392
μ/mm^{-1}	3.021
F(000)	406.0
Crystal size/mm^3	0.34 × 0.27 × 0.033
Radiation	Cu Kα (λ = 1.54184)
2Θ range for data collection/°	6.11 to 153.614
Index ranges	$-9 \le h \le 9$, $-10 \le k \le 10$, $-18 \le l \le 17$
Reflections collected	20909
Independent reflections	3618 [R_{int} = 0.0245, R_{sigma} = 0.0161]
Data/restraints/parameters	3618/169/261
Goodness-of-fit on F^2	1.069
Final R indexes [I>=2σ (I)]	R_1 = 0.0314, wR_2 = 0.0834
Final R indexes [all data]	R_1 = 0.0326, wR_2 = 0.0842
Largest diff. peak/hole / e Å^{-3}	0.33/-0.27

CCDC Deposition Number: 2372111

tetramethylpyrazine: phenazine: resorcinol: pyrene (1:1:2:2)

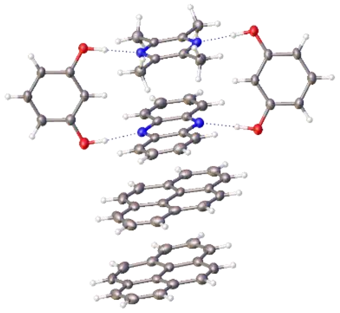

Empirical formula	$C_{64}H_{52}N_4O_4$
Formula weight	941.09
Temperature/K	150.00(10)
Crystal system	monoclinic
Space group	$P2_1/c$
a/Å	17.3302(2)
b/Å	14.5941(2)
c/Å	18.2582(2)
α/°	90
β/°	90.1780(10)
γ/°	90
Volume/Å^3	4617.82(10)
Z	4
ρ_{calc}g/cm^3	1.354
μ/mm^{-1}	0.667
F(000)	1984.0
Crystal size/mm^3	0.12 × 0.12 × 0.076
Radiation	Cu Kα (λ = 1.54184)
2Θ range for data collection/°	5.1 to 153.628
Index ranges	-20 ≤ h ≤ 21, -17 ≤ k ≤ 18, -22 ≤ l ≤ 22
Reflections collected	68989
Independent reflections	9281 [R_{int} = 0.0406, R_{sigma} = 0.0259]
Data/restraints/parameters	9281/576/665
Goodness-of-fit on F^2	1.044
Final R indexes [I>=2σ (I)]	R_1 = 0.0387, wR_2 = 0.1091
Final R indexes [all data]	R_1 = 0.0463, wR_2 = 0.1145
Largest diff. peak/hole / e Å^{-3}	0.18/-0.20

CCDC Deposition Number: 2372112

2-chlororesorcinol: tetramethylpyrazine: 2,2′-bipyridine: 1,2-bis(4-pyridyl)ethane (1:0.5:0.5:0.5)

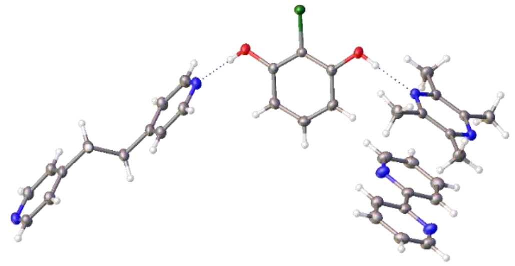

Empirical formula	$C_{42}H_{42}Cl_2N_6O_4$
Formula weight	765.71
Temperature/K	149.98(10)
Crystal system	triclinic
Space group	P-1
a/Å	7.4491(2)
b/Å	8.7828(2)
c/Å	14.3891(3)
α/°	98.447(2)
β/°	96.170(2)
γ/°	92.494(2)
Volume/Å^3	924.10(4)
Z	1
ρ_{calc}g/cm^3	1.376
μ/mm^{-1}	2.006
F(000)	402.0
Crystal size/mm^3	0.16 × 0.091 × 0.017
Radiation	Cu Kα (λ = 1.54184)
2Θ range for data collection/°	6.252 to 154.42
Index ranges	-9 ≤ h ≤ 8, -10 ≤ k ≤ 10, -8 ≤ l ≤ 17
Reflections collected	9323
Independent reflections	3616 [R_{int} = 0.0293, R_{sigma} = 0.0328]
Data/restraints/parameters	3616/162/252
Goodness-of-fit on F^2	1.054
Final R indexes [I>=2σ (I)]	R_1 = 0.0321, wR_2 = 0.0777
Final R indexes [all data]	R_1 = 0.0376, wR_2 = 0.0805
Largest diff. peak/hole / e Å^{-3}	0.22/-0.24

CCDC Deposition Number: 2372113

2-bromoresorcinol: tetramethylpyrazine: 2,2'-bipyridine: 1,2-bis(4-pyridyl)ethane (1:0.5:0.5:0.5)

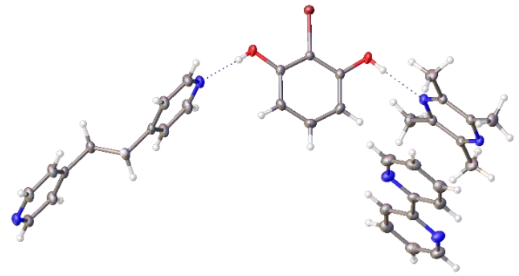

Empirical formula	$C_{42}H_{42}Br_2N_6O_4$
Formula weight	854.63
Temperature/K	150.00(10)
Crystal system	triclinic
Space group	P-1
a/Å	7.5285(2)
b/Å	8.8116(2)
c/Å	14.4497(3)
α/°	97.681(2)
β/°	96.463(2)
γ/°	91.933(2)
Volume/Å^3	942.77(4)
Z	1
ρ_{calc}g/cm^3	1.505
μ/mm^{-1}	3.146
F(000)	438.0
Crystal size/mm^3	0.27 × 0.19 × 0.17
Radiation	Cu Kα (λ = 1.54184)
2Θ range for data collection/°	6.216 to 154.472
Index ranges	-9 ≤ h ≤ 9, -10 ≤ k ≤ 10, -17 ≤ l ≤ 17
Reflections collected	21747
Independent reflections	3717 [R_{int} = 0.0327, R_{sigma} = 0.0170]
Data/restraints/parameters	3717/162/252
Goodness-of-fit on F^2	1.080
Final R indexes [I>=2σ (I)]	R_1 = 0.0231, wR_2 = 0.0599
Final R indexes [all data]	R_1 = 0.0234, wR_2 = 0.0601
Largest diff. peak/hole / e Å^{-3}	0.34/-0.48

S5.9 New Quaternary Co-Crystals from Quaternary Co-Crystal Screening

CCDC Deposition Number: 2372114

2-chlororesorcinol: tetramethylpyrazine: 2,2'-bipyridine: 4,4'-bipyridine (1:0.5:0.5:0.5)

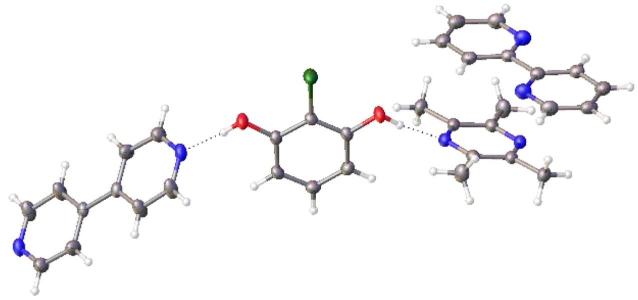

Empirical formula	$C_{20}H_{19}ClN_3O_2$
Formula weight	368.83
Temperature/K	149.95(11)
Crystal system	triclinic
Space group	P-1
a/Å	7.3897(5)
b/Å	9.2056(7)
c/Å	13.3842(8)
α/°	90.180(5)
β/°	100.544(5)
γ/°	94.752(6)
Volume/Å^3	891.88(11)
Z	2
ρ_{calc}g/cm^3	1.373
μ/mm^{-1}	2.058
F(000)	386.0
Crystal size/mm^3	0.19 × 0.077 × 0.052
Radiation	Cu Kα (λ = 1.54184)
2Θ range for data collection/°	6.718 to 154.742
Index ranges	-9 ≤ h ≤ 7, -11 ≤ k ≤ 11, -16 ≤ l ≤ 16
Reflections collected	8822
Independent reflections	3475 [R_{int} = 0.0298, R_{sigma} = 0.0348]
Data/restraints/parameters	3475/153/243
Goodness-of-fit on F^2	1.082
Final R indexes [I>=2σ (I)]	R_1 = 0.0373, wR_2 = 0.0992
Final R indexes [all data]	R_1 = 0.0418, wR_2 = 0.1022
Largest diff. peak/hole / e Å^{-3}	0.27/-0.33

CCDC Deposition Number: 2428849

2-bromoresorcinol: tetramethylpyrazine: 2,2'-bipyridine: 4,4'-bipyridine (1:0.5:0.5:0.5)

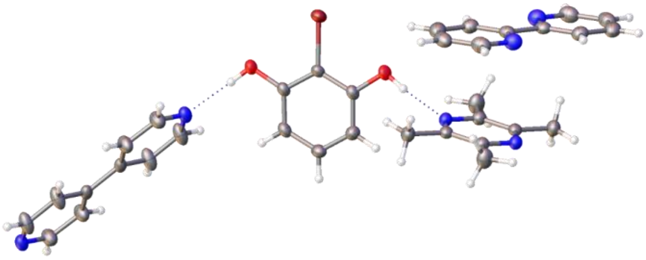

Empirical formula	$C_{40}H_{38}Br_2N_6O_4$
Formula weight	826.58
Temperature/K	150.00(10)
Crystal system	triclinic
Space group	P-1
a/Å	7.5417(3)
b/Å	9.1395(4)
c/Å	13.4898(7)
α/°	89.988(4)
β/°	78.127(4)
γ/°	85.258(3)
Volume/Å^3	906.67(7)
Z	1
ρ_{calc}g/cm^3	1.514
μ/mm^{-1}	3.251
F(000)	422.0
Crystal size/mm^3	0.14 × 0.08 × 0.04
Radiation	Cu Kα (λ = 1.54184)
2Θ range for data collection/°	6.696 to 153.896
Index ranges	-9 ≤ h ≤ 8, -11 ≤ k ≤ 11, -16 ≤ l ≤ 16
Reflections collected	9876
Independent reflections	3509 [R_{int} = 0.0306, R_{sigma} = 0.0325]
Data/restraints/parameters	3509/153/239
Goodness-of-fit on F^2	1.053
Final R indexes [I>=2σ (I)]	R_1 = 0.0332, wR_2 = 0.0854
Final R indexes [all data]	R_1 = 0.0375, wR_2 = 0.0882
Largest diff. peak/hole / e Å^{-3}	0.35/-0.47

CCDC Deposition Number: 2428850

2-methylresorcinol: tetramethylpyrazine: 2,2’-bipyridine: 4,4’-bipyridine (1:0.5:0.5:0.5)

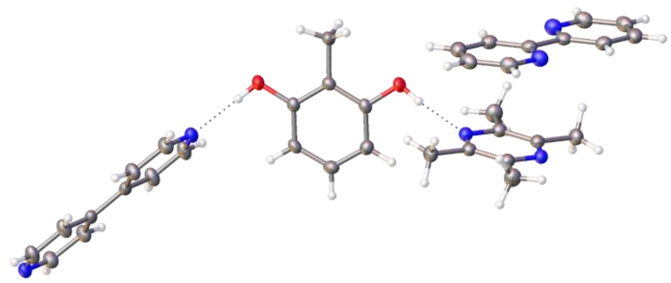

Empirical formula	$C_{42}H_{44}N_6O_4$
Formula weight	696.83
Temperature/K	150.00(10)
Crystal system	triclinic
Space group	P-1
a/Å	7.3943(3)
b/Å	9.0892(4)
c/Å	13.4707(5)
α/°	89.892(3)
β/°	80.258(3)
γ/°	84.912(3)
Volume/Å^3	888.69(6)
Z	1
ρ_{calc}g/cm^3	1.302
μ/mm^{-1}	0.682
F(000)	370.0
Crystal size/mm^3	0.13 × 0.08 × 0.03
Radiation	Cu Kα (λ = 1.54184)
2Θ range for data collection/°	6.658 to 155.014
Index ranges	-9 ≤ h ≤ 9, -11 ≤ k ≤ 9, -14 ≤ l ≤ 16
Reflections collected	8805
Independent reflections	3493 [R_{int} = 0.0283, R_{sigma} = 0.0354]
Data/restraints/parameters	3493/156/240
Goodness-of-fit on F^2	1.067
Final R indexes [I>=2σ (I)]	R_1 = 0.0397, wR_2 = 0.1032
Final R indexes [all data]	R_1 = 0.0483, wR_2 = 0.1086
Largest diff. peak/hole / e Å^{-3}	0.17/-0.19

CCDC Deposition Number: 2428851

orcinol: tetramethylpyrazine: 2,2'-bipyridine: 4,4'-bipyridine (1:0.5:0.5:0.5)

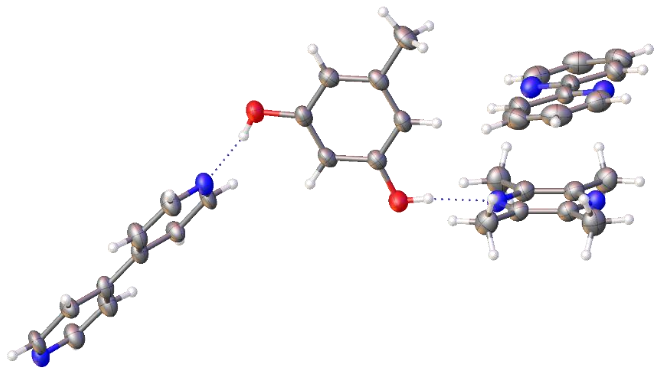

Empirical formula	$C_{42}H_{44}N_6O_4$
Formula weight	696.83
Temperature/K	150.00(10)
Crystal system	triclinic
Space group	P-1
a/Å	7.8893(2)
b/Å	9.2195(4)
c/Å	13.1011(4)
α/°	85.101(3)
β/°	81.385(3)
γ/°	75.206(3)
Volume/Å^3	909.82(6)
Z	1
ρ_{calc}g/cm^3	1.272
μ/mm^{-1}	0.666
F(000)	370.0
Crystal size/mm^3	0.16 × 0.09 × 0.06
Radiation	Cu Kα (λ = 1.54184)
2Θ range for data collection/°	6.832 to 154.372
Index ranges	-9 ≤ h ≤ 9, -10 ≤ k ≤ 9, -15 ≤ l ≤ 16
Reflections collected	9862
Independent reflections	3518 [R_{int} = 0.0218, R_{sigma} = 0.0235]
Data/restraints/parameters	3518/282/337
Goodness-of-fit on F^2	1.050
Final R indexes [I>=2σ (I)]	R_1 = 0.0386, wR_2 = 0.1015
Final R indexes [all data]	R_1 = 0.0439, wR_2 = 0.1060
Largest diff. peak/hole / e Å^{-3}	0.21/-020

S6. References

1. CrysAlisPRO, Oxford Diffraction /Agilent Technologies UK Ltd, Yarnton, England.
2. J. Cosier, A. M. Glazer, A Nitrogen-Gas-Stream Cryostat for General X-ray Diffraction Studies, *J. App. Cryst.,* 1986, **19**, 105–107.
3. G. M. Sheldrick, Crystal Structure Refinement with SHELXL*, Acta Cryst*., 2015, C**71**, 3–8.
4. G. M. Sheldrick, SHELXT-Integrated Space-Group and Crystal-Structure Determination, *Acta Cryst.,* 2015, A**71**, 3–8.
5. O. V. Dolomanov, L. J. Bourhis, R. J. Gildea, J. A. K. Howard, H. Puschmann, OLEX2: A Complete Structure Solution, Refinement and Analysis Program, *J. App. Cryst.,* 2009, **42**, 339–341.
